# Culture-Independent Exploration of the Hypersaline Ecosystem Indicates the Environment-Specific Microbiome Evolution

**DOI:** 10.3389/fmicb.2021.686549

**Published:** 2021-10-28

**Authors:** Priyanka Mehta, Monika Yadav, Vasim Ahmed, Khushboo Goyal, Rajesh Pandey, Nar Singh Chauhan

**Affiliations:** ^1^Genomics and Molecular Medicine, INtegrative GENomics of HOst-PathogEn (INGEN-HOPE) Laboratory, CSIR-Institute of Genomics and Integrative Biology (CSIR-IGIB), New Delhi, India; ^2^Department of Biochemistry, Maharshi Dayanand University, Rohtak, India; ^3^Academy of Scientific and Innovative Research (AcSIR), Ghaziabad, India

**Keywords:** hypersaline lake, extremophiles, multi-omics analysis, microbiome, microbiome physiology, stress response and adaptation, metagenome

## Abstract

Sambhar Salt Lake, situated in the state of Rajasthan, India is a unique temperate hypersaline ecosystem. Exploration of the salt lake microbiome will enable us to understand microbiome functioning in nutrient-deprived extreme conditions, as well as enrich our understanding of the environment-specific microbiome evolution. The current study has been designed to explore the Sambhar Salt Lake microbiome with a culture-independent multi-omics approach to define its metagenomic features and prevalent metabolic functionaries. The rRNA feature and protein feature-based phylogenetic reconstruction synchronously (*R* = 0.908) indicated the dominance of the archaea (Euryarchaeota) and bacteria (Firmicutes, Proteobacteria, Bacteroidetes, and Actinobacteria). Metabolic reconstruction identified selective enrichment of the protein features associated with energy harvesting and stress tolerance (osmotic, oxidative, metal/metalloid, heat/cold, antibiotic, and desiccation). Metabolites identified with metabolome analysis confirmed physiological adaptation of the lake microbiome within a hypersaline and nutrient-deprived environment. Comparative metagenomics of the 212 metagenomes representing freshwater, alkaline, and saline ecosystem microbiome indicated the selective enrichment of the microbial groups and genetic features. The current study elucidates microbiome functioning within the nutrient-deprived harsh ecosystems. In summary, the current study harnessing the strength of multi-omics and comparative metagenomics indicates the environment-specific microbiome evolution.

## Introduction

Sambhar Lake is the largest inland, hypersaline lake (salt content ∼30 g/L), situated at the semi-arid Aravalli schists of Rajasthan (longitude 75°05′E, latitude 26°58′N), India. It covers approximately 230 sq. km area with an average depth of 1 m (Yadav and Sarin, 2009). Due to the high evaporation rate, there is excessive deposition of salts (sodium chloride, sodium carbonate, etc.) and metals and metalloids (cobalt, iron, zinc, copper, chromium, lead, arsenic, etc.) (Pathak and Cherekar, 2015). The lake has a mass bloom of halophilic algae, green, and purple-sulfur photosynthetic bacteria, halophilic archaea, etc. (Shukal and Rahaman, 2006). Thus, the hypersaline environment provides a unique opportunity to study the functional attributes of the microbes present in such a harsh environment (Oren, 2010). Microbes living in such environments should adapt themselves to tolerate such an extreme extracellular osmolarity (Ahmed et al., 2018). Various halophilic microorganisms like representatives of the genera *Bacillus, Salinicoccus*, *Marinobacter, Virgibacillus, Halobacillus, Geomicrobium, Chromohalobacter, Oceanobacillus, Halomonas, Staphylococcus*, and *Euhalothece*, were isolated from Sambhar Lake (Upasani, 2008; Bhatt et al., 2016; Sangeeta et al., 2016) and characterized for industrial applications (Singh and Jha, 2016). However, most of these studies were performed in isolation with the culture-dependent approach (Singh and Jha, 2016). They failed to explain microbial adaptation strategies (osmotolerance, oxidative stress tolerance, and metal/metalloid stress tolerance mechanisms), energy generation, and channelization processes in the salt lake microbiome. The evolution of such strategies by microbes is of utmost importance for their survival in such an extreme environment (Wood, 2015). Exploration of the salt lake microbiome will enrich the understanding of microbiome functioning in nutrient-deprived extreme conditions (Naghoni et al., 2017). Metagenomics and metabolomics are the pioneer culture-independent tools to explore the untapped microbial world to unveil microbiome genetic composition and functional metabolic pathways (Ahmed et al., 2018; Yadav et al., 2020, 2021). Metagenomic exploration of the freshwater, alkaline, and saline water ecosystem has identified unique inhabitants and their unique genetic machinery (Poli et al., 2017). A comparative analysis of these metagenomes could extend knowledge about ecosystem-specific microbiome evolution (Gao et al., 2011). Hereby, the current study has been proposed to explore the Sambhar Salt Lake microbiome with a multi-omics approach to understand the microbiome functioning in such an extreme environment. The microbiome comparison will define the ecosystem-specific microbiome composition and its metabolic functionaries. The current study will enrich our understanding of the microbiome evolution in the hypersaline environment. All this information could be harnessed for various biotechnology applications.

## Methodology

### Hypersaline Lake Water Sample Collection and Metagenomic DNA Isolation

The hypersaline lake water samples were collected at the depth of 1 m from the five different locations of the salt lake, Sambhar, Rajasthan (longitude 75°05′E, latitude 26°58′N) in sterile containers in April 2015 (temperature 38°C). Samples were transported at room temperature and processed immediately for the extraction of metagenomic DNA and total metabolites. Microbial cells from the 1-L water sample were harvested as a pellet after centrifugation at 14,000 revs min^–1^ for 10 min. The microbial pellet was processed for DNA isolation following the alkaline lysis method (Ahmed et al., 2018) (Supplementary Method 1). The metagenomic DNA isolated from the replicates were pooled before the shotgun sequencing.

### Metagenome Sequencing and Sequence Analysis

Qualitative and quantitative analysis of the metagenomic DNA was performed with agarose gel [0.8% (w/v)] electrophoresis and Qubit^TM^ dsDNA High Sensitivity Assay Kit using a Qubit 2.0 fluorometer (Thermo Scientific, MA, United States). The sequencing library was made using Illumina Nextera XT DNA Library Prep Kit (FC-131-1096) as per reference guide (15031942 v05). Initially, 250 ng of metagenomic DNA was subjected to transposome-mediated tagmentation. The tagmented DNA was then amplified followed by purification using Agencourt AMPure XP beads (A63881). Nextera XT Index Kit v2 Set A (FC-131-2001) indexes were used for indexing the samples. The quality and quantity of the sequencing library was checked using Agilent 2100 Bioanalyzer with a high sensitivity DNA chip and the Qubit dsDNA HS Assay kit, respectively. A loading concentration of 10 pM was prepared by denaturing and diluting the libraries in accordance with the MiSeq System Denature and Dilute Libraries Guide (Illumina, Document no. 15039740 v10). Sequencing was performed on the MiSeq system, using the MiSeq Reagent Kit v3 (300 cycles) at 2 × 101 bp read length. Analysis of the metagenomic dataset was performed following standard methodology (Carlos et al., 2014; Karimi et al., 2017).

The sequence dataset (Supplementary Table 1) was uploaded into the Metagenome Rapid Annotation using Subsystem Technology (MG-RAST) server 4.0.3^1^ (Meyer et al., 2008) for quality filtering (reads with Phred score > 15 were trimmed for removing adapter contamination and screened to remove host genomic DNA sequences) (Yadav et al., 2020). The quality-filtered sequences were also used to perform the genome assembly using MEGAHIT assembler (Li et al., 2015) (Supplementary Method 2). The assembled dataset was also uploaded into the Metagenome Rapid Annotation using Subsystem Technology (MG-RAST) server 4.0.3 for downstream processing (Meyer et al., 2008). The quality-filtered sequences were used for 16S and 18S rRNA feature identification by searching the similarity (cutoff% >70%) of metagenome sequences with ribosomal sequences from the M5nr database (Wilke et al., 2012). Potential ribosomal RNA features were clustered based on their similarity (percentage >97%), and a representative sequence was checked for its homologs using the BLAT algorithm in the Ribosomal Database Project (RDP) (*e*-value < 10^–5^, sequence similarity >60%, and word size >15 bp). Putative protein features were identified with FragGeneScan 1.3.1 (Rho et al., 2010). Predicted protein features were clustered (90% identity) and processed for their similarity using BLAT algorithm against the M5NR protein database, RefSeq database, and Subsystems database (*e*-value < 10^–5^, minimum identity >60%). Taxonomic and functional affiliation of protein features was predicated with their feature abundance profile, data source abundance profile, and lowest common ancestor (LCA) abundance profile (Yadav et al., 2020).

### Identification and Mapping of Features Associated With Adaptive Physiology

Functional annotation of predicted protein features was performed by searching homologs in the Subsystems database (Overbeek et al., 2005) using stringent search parameters (*e*-value < 10^–5^, minimum identity >60%, word size >15). Identified protein features were manually curated from the annotated protein features and mapped onto the metabolic pathway. Phylogenetic characterization of the protein features was performed by searching their homologs in RefSeq non-redundant protein database using KAIJU webserver 1.8 (Sequence similarity >75% and match size >11, *e*–value <0.01) (Menzel et al., 2016).

### Metabolic Profiling of Sambhar Salt Lake Microbiome

The Sambhar Lake water was centrifuged at 14,000 revs min^–1^ for 10 min to collect microbial pellet. The microbial pellet was quenched with 60% aqueous methanol solution (−48°C) and processed for metabolite extraction (Yadav et al., 2021). High-performance liquid chromatography coupled to quadrupole-time of flight mass spectrometry (HPLC/Q-TOF MS), possessing an Exion LC system integrated with X-500 QTOF (SCIEX Technology, United States) was used to obtain the metabolic profiles in the filtered supernatant. Both negative and positive modes of electrospray ionization (ESI) were used to capture the metabolic profile. LC-MS spectra were analyzed with an XCMS server (Yadav et al., 2020).

### Comparative Metagenome Analysis

Metagenomic datasets from freshwater, saline water, and alkaline water microbiome were compared to check their similarity and uniqueness at phylogenetic and metabolic levels. A total of 212 metagenomic datasets representing freshwater (131), saline water (67), and alkaline water (14) ecosystems (Supplementary Table 2) were extracted from the MG-RAST server to identify rRNA and protein features (Meyer et al., 2008). Predicted rRNA features were clustered and checked for their homologs in the Ribosomal Database Project (RDP) (*e*-value < 10^–5^, sequence similarity >60%, and word size >15 bp). Predicted protein features were clustered and searched for their homologs in the RefSeq database (O’Leary et al., 2016). Functional annotation of the predicted protein features was performed after searching homologs (*e*-value < 10^–5^, minimum identity >60%, and word size >15) in the Subsystems database. The stress response features like oxidative stress, osmotic stress, and resistance to antibiotics/toxic compounds were also extracted from the Subsystem database for all the ecosystems. The row length normalized data were used for principal component analysis (PCA) plots using PAST v4.03 software (Hammer et al., 2001). Statistical significance between ecosystems was measured using PERMANOVA (for all subsystem features at levels 1 and 2, the stress responses protein features, and phylogenetic data at phylum and class level of taxonomic hierarchy). Ternary plots were plotted for the normalized abundance profile data using the Ternary Plot tool^2^. While comparing the Subsystem features and stress responses, a non-parametric Kruskal–Wallis test for equal medians was performed along with Mann–Whitney pairwise comparisons test considering *p*-value < 0.05 as significant. The figures were modified using the Inkscape 1.0.1 software.

## Results

### Sambhar Salt Lake Metagenome

Sambhar Lake water metagenomic DNA was sequenced with the MiSeq system (Illumina, United States) using paired-end sequencing that generated 55,718,072 high-quality sequences (6,025,375,597 base pairs) (Supplementary Table 1). Analysis of the quality filtered dataset identified 7,034 ribosomal RNA features. Even a total of 3,025,041 functional categories were identified in the metagenome dataset (Supplementary Table 1). Rarefaction curve analysis indicated that the curve reached toward attaining a plateau (Supplementary Figure 1). This analysis indicated the sufficiency of the current metagenomic data to provide a holistic overview of the salt lake microbiome.

### Phylogenetic Characterization of Sambhar Salt Lake Metagenome

Ribosomal features shared their homology with Archaea (37.49%) and Bacteria (55.66%), as well as with Eukaryota domains, representing a total of 18 different phyla (Supplementary Table 3). Among these 18 phyla, the majority of the sequences were affiliated with Euryarchaeota (36.6%), Firmicutes (20.18%), Proteobacteria (14.90%), Bacteroidetes (3.50%), and Actinobacteria (2.6%). The phylogenetic affiliation of rRNA features at the class level of taxonomic hierarchy indicated the relative abundance of Halobacteria (36.59%), followed by Clostridia (14.46%), Gammaproteobacteria (10.13%), Chlorophyceae (5.87%), Bacilli (4.6%), Alphaproteobacteria (2.59%), Actinobacteria (2.59%), Sphingobacteria (1.676%), and Flavobacteria (1.35%) (Supplementary Table 3). *Halorubrum* and *Halanaerobium* represented 1/3 of the total microbial members of the Sambhar Lake microbiome (Supplementary Table 3).

Phylogenetic affiliation of the protein features within the RefSeq database indicated taxonomic affiliation within four domains [Archaea (54.84%), bacteria (44.14%), Eukaryota (0.95%), and viruses (0.05%)] representing 62 different phyla (Supplementary Table 4). The majority of the protein features were affiliated with Euryarchaeota (53.76%), Proteobacteria (19.14%), Firmicutes (12.15%), Actinobacteria (1.35%), and Cyanobacteria (1%). Phylogenetic analysis of the assembled dataset also provided a similar representation. A good correlation (*R* = 0.953) was observed among the phylogeny outcomes of the protein features identified in the raw reads and assembled datasets. It indicates the phylogenetic diversity representation is free from the dataset-derived bias.

Even a good positive correlation (*R* = 0.908) was observed among ribosomal features and protein features-based microbial diversity analysis. This highlighted the significant concordance among the microbial diversity observed with these features.

### Metabolic Characterization of Sambhar Salt Lake Metagenome

Sambhar Salt Lake is a unique temperate hypersaline ecosystem where inhabiting microbes successfully survive in harsh conditions (Upasani, 2008; Bhatt et al., 2016; Sangeeta et al., 2016). Microbial communities in harsh ecosystems harbor a plethora of information to elucidate their physiological functions to enrich our understanding of ecosystem functioning in hypersaline temperate ecosystems (Naghoni et al., 2017). SEED and Subsystem technology was employed for the annotation of the identified protein features (Overbeek et al., 2014) and a total of 3,025,041 protein families were observed in the metagenomic dataset. These protein families were clustered into 28 categories of Subsystem hierarchy (Supplementary Table 5). Among observed functional categories, ∼49% of the total protein features were associated with biomolecule metabolism, while 11.05% protein features were associated with secondary metabolism, elemental metabolism, and pigment metabolism (Supplementary Table 5). The 5.39% of total protein features were also found associated with stress resistance, virulence, and genetic transformations (Supplementary Table 5). The percentage of protein features for stress resistance, virulence, and genetic transformation in the current metagenomic dataset was higher as compared to other ecosystems (Yadav et al., 2020). A higher percentage of these protein features might be extending physiological flexibility to the salt lake microbes to thrive in the hypersaline environment.

Subsystem-based clustering of the protein features identified from raw reads and assembled dataset have a good correlation (*R* = 0.988). This outcome indicated that the analysis outcome is free from the bias generally introduced due to the nature of datasets used for the study.

### Stress Response Physiology of the Sambhar Salt Lake Microbiome

**FIGURE 1:**
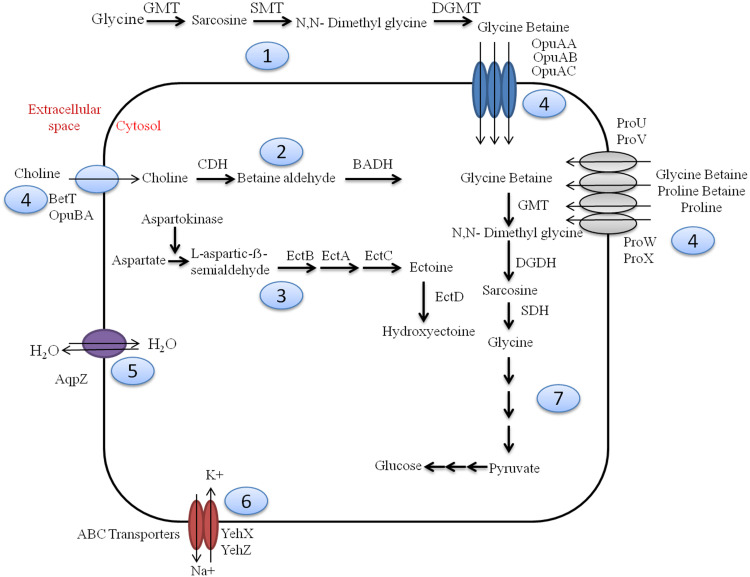
Overview of osmotic stress tolerance mechanisms prevalent within Sambhar hypersaline lake microbiome. Sambhar salt lake microbiome has genes clusters encoding proteins for the biosynthesis of osmolytes like Glycine Betaine from glycine (1) & choline (2), Ectoine and Hydroxyectoine (3); transport of various osmolytes (proline, glycine betaine, choline, etc.) (4), water (5), ions (6); and metabolism of glycine betaine to generate energy-rich metabolic substrate (7). GMT, glycine *N*-methyltransferase; SMT, sarcosine *N*-methyltransferase; DGMT, dimethylglycine *N*-methyltransferase; DGDH, dimethylglycine dehydrogenase; SDH, sarcosine dehydrogenase; CDH, choline dehydrogenase; BADH, betaine aldehyde dehydrogenase; EctABCD, ectoine biosynthetic enzymes.

Sambhar Lake water microbiome elucidated the presence of 122,855 protein features whose homologs were characterized to play a significant role in stress responses such as acid stress (1%), cold shock (1.1%), desiccation stress (0.02%), detoxification (1.4%), heat shock (19.9%), osmotic stress (18.6%), oxidative stress (39.7%), and periplasmic stress (2.8%). Homologs of the osmotic stress response protein features have been characterized for synthesis and uptake of various osmolytes (Betaine, Choline, Ectoine, and osmoregulator periplasmic glucans), while several protein features were identified to be involved in osmoregulation through the synthesis of ABC transporter proteins, osmotic stress cluster proteins, etc. (Table 1 and Supplementary Figure 2). These metabolic features either independently or synchronously might be allowing the salt lake microbes to thrive in saline conditions (Figure 1). Salt is a well-established factor to induce oxidative stress within living cells (AbdElgawad et al., 2016). Hereby, to survive and adapt to the hypersaline conditions, the microbes should equip themselves with oxidative stress response machinery (Ma et al., 2020). Metagenomic analysis of the Sambhar hypersaline lake microbiome has identified a 39.7% of the total stress response protein features whose homologs have been characterized for maintaining reduction–oxidation balance within microbial cells (Table 2). Analysis of metagenome also identified features known to overcome acid stress, temperature stress, desiccation stress, periplasmic stress (Supplementary Table 6), antibiotic stress (Table 3), and metal and metalloid stress (Table 4). Phylogenetic affiliation of these adaptive features indicated their distribution among all microbial groups of the Sambhar Salt Lake microbiome. The presence of these features indicated microbial survival strategies to overcome environmental stresses of the hypersaline environment.

**TABLE 1:** Metagenomic features associated with osmotic stress response physiology in Sambhar Salt Lake microbiome.

Sr. no.	Osmotic stress response physiology	Identified protein features	Phylogenetic afflation (% abundance)
(1)	Betaine biosynthesis from glycine	Glycine *N*-methyltransferase (GMT), sarcosine *N*-methyltransferase (SMT), dimethylglycine *N*-methyltransferase (DGMT)	Proteobacteria (73%), Actinobacteria (7%), Cyanobacteria (2%), Firmicutes (1.15%), and Planctomycetes (1.15%)

(2)	Choline and Betaine Metabolism	Glycine betaine ABC transport system, choline ABC transport system, betaine aldehyde dehydrogenase (BADH) and Choline dehydrogenase (CDH), glycine betaine demethylase subunit A (GbcA), glycine betaine demethylase subunit B (GbcB), sarcosine oxidase alpha subunit, sarcosine oxidase beta subunit and sarcosine oxidase delta subunit	Proteobacteria (56%), Firmicutes (15%), Euryarchaeota (7%), Actinobacteria (2%), Bacteroidetes (2%), and others (>1% belongs to Balneolaeota, Eukaryota, Haloplasmatales, Spirochaetes, and Verrucomicrobia)

(3)	Ectoine and Hydroxyectoine Biosynthesis	Aspartokinase; L-2,4-diaminobutyric acid acetyltransferase (EctA), diaminobutyrate-pyruvate aminotransferase (EctB), L-ectoine synthase (EctC), regulatory protein (EctR), and ectoine hydroxylase (EctD)	Proteobacteria (83%), Chloroflexi (4.38%), Actinobacteria (1%), and others (<1% Firmicutes, Euryarchaeota)

(4)	Biosynthesis of osmoregulated periplasmic glucans (OPGs)	Glucans biosynthesis glucosyl transferase H, cyclic beta-1,2-glucan synthase, glucans biosynthesis protein G precursor, glucans biosynthesis protein D precursor, phosphoglycerol transferase I, Beta-(1– > 2) glucan export ATP-binding/permease protein NdvA	Proteobacteria (68%), FCB groups (3.53%), Firmicutes (1%), Bacteroidetes (3%), and others microbial groups (Euryarchaeota, Balneolaeota, Acidobacteria, Actinobacteria, Planctomycetes, and Nitrospirae)

(5)	Osmoprotectant ABC transporters	Osmoprotectant ABC transporter inner membrane protein YehW, osmoprotectant ABC transporter ATP-binding subunit YehX, and osmoprotectant ABC transporter binding protein YehZ	Proteobacteria (80%), Actinobacteria (1.1%), Euryarchaeota (1%), and other microbial groups (Firmicutes, Thermotogae, etc.)

(6)	Osmotic stress cluster proteins	Membrane protein precursor, Aquaporin Z, and Propanediol diffusion facilitator	Proteobacteria (7%), FCB groups (67%), Gemmatimonadetes (25.60%), Bacteroidetes (25%), Candidate division Zixibacteria (12.80%), Balneolaeota (1.2%), and other microbial groups (Firmicutes, Eukaryota, Euryarchaeota, and Nitrospinae/Tectomicrobia)

**TABLE 2:** Metagenomic features associated with oxidative stress response physiology in Sambhar Salt Lake microbiome.

Sr. no.	Oxidative stress response features	Identified protein features	Phylogenetic afflation (% abundance)
(1)	Glutathione biosynthesis	5-oxoprolinase, gamma-glutamyl cyclotransferase, gamma-glutamyltranspeptidase, glutamate-cysteine ligase, glutamate-cysteine ligase archaeal, glutathione biosynthesis bifunctional protein, glutathione synthetase, glutathione *S*-transferase	Proteobacteria (50%), Euryarchaeota (26.7%), Unclassified Bacteria (8.6%), Unclassified Archaea (4.6%), Actinobacteria (1.8%), Firmicutes (1.7%), Bacteroidetes/Chlorobi group (0.9%), Cyanobacteria/Melainabacteria group (0.8%), Balneolaeota (0.4%), Terrabacteria group (0.25%), Opisthokonta (0.25%), Planctomycetes (0.12%), Nitrospinae/Tectomicrobia group (0.1%), Verrucomicrobia (0.03%), Deinococcus-Thermus (0.03%), Rhodophyta (0.03%), Viridiplantae (0.02%), Alveolata (0.02%), FCB group (0.02%), Nitrospirae (0.01%), Spirochaetes (0.01%), Acidobacteria (0.01%), Chloroflexi (0.01%), and Haloplasmatales (0.01%).

(2)	Glutathione redox and Non-redox reactions	Glutathione *S*-transferase, zeta hydroxyacylglutathione hydrolase, lactoylglutathionelyase, phytochelatin synthase, SAM-dependent methyltransferase 2, hydroxyacylglutathione hydrolase, glutaredoxin, glutaredoxin 3, glutathione peroxidase, glutathione reductase	Actinobacteria (0.45%), Bacteroidetes (1.32%), Candidatus Nanohaloarchaeota (0.15%), Cyanobacteria/Melainabacteria group (0.78%), Euryarchaeota (38.4%), Firmicutes (3.57%), Proteobacteria (53.47%), Environmental samples (1.75%), and Armatimonadetes (0.09%).

(3)	Rubrerythrin metabolism	Alkyl hydroperoxide reductase subunit C-like protein, Fe-S oxidoreductase-like protein in Rubrerythrin cluster, Probable peroxiredoxin, rubredoxin, rubredoxin-NAD(+) reductase, rubrerythrin, superoxide reductase, rubredoxin-oxygen oxidoreductase	Euryarchaeota (44.5%), Firmicutes (15.5%), Proteobacteria (8%), Unclassified Bacteria (4.4%), Bacteroidetes/Chlorobi group (3.3%), Actinobacteria (1.9%), Unclassified Archaea (1.9%), Chloroflexi (1.3%), Fornicata (0.7%), Candidatus Omnitrophica (0.5%), Cyanobacteria/Melainabacteria group (0.5%), Terrabacteria group (0.4%), Thermotogae (0.4%), Spirochaetes (0.3%), Tenericutes (0.2%), Planctomycetes (0.1%), Opisthokonta (0.1%), TACK group (0.1%), Acidobacteria (0.03%), Aquificae (0.03%), Deinococcus-Thermus (0.03%), Viridiplantae (0.03%), and Stramenopiles (0.03%)

(4)	Glutaredoxin metabolism	Glutaredoxin, glutaredoxin 1, glutaredoxin 3 (grx2), glutaredoxin-like protein (NrdH), glutaredoxin-related protein, probable monothiol glutaredoxin (GrlA)	Proteobacteria (42.6%), Euryarchaeota (38.2%), Unclassified Archaea (5.9%), Firmicutes (4.4%), Cyanobacteria/Melainabacteria group (2.9%), Actinobacteria (0.7%), Terrabacteria group (0.7%), and Unclassified Bacteria (0.7%)

(5)	Glutathionyl spermidine and trypanothione metabolism	Glutathionyl spermidine amidohydrolase, glutathionyl spermidine synthase, uncharacterized GST-like protein (yghU)	Proteobacteria (100%)

(6)	Mycothiol metabolism	Acetyl-CoA: Cys-GlcN-Ins acetyltransferase, mycothiol synthase (MshD), formaldehyde dehydrogenase (MscR), L-cysteine:1D-myo-inosityl 2-amino-2-deoxy-alpha-D-glucopyranoside ligase (MshC), mycothiol *S*-conjugate amidase (Mca), *N*-acetyl-1-D-myo-inosityl-2-amino-2-deoxy-alpha-D-glucopyranoside deacetylase (MshB), NADPH-dependent mycothiol reductase (Mtr), *S*-nitrosomycothiol reductase (MscR)	Actinobacteria (38.1%), Euryarchaeota (14.3%), Bacteroidetes/Chlorobi group (9.5%), Unclassified Bacteria (4.8%), Proteobacteria (4.8%), DPANN group (4.8%), and Unclassified Archaea (4.8%)

(7)	CoA disulfide thiol-disulfide redox system	CoA-disulfide reductase. Polysulfide binding and transferase domain	Euryarchaeota (61.6%), Proteobacteria (11.5%), Firmicutes (9.6%), Bacteroidetes/Chlorobi group (5.6%), Unclassified Archaea (4.6%), Unclassified Bacteria (1.6%), Actinobacteria (0.9%), Terrabacteria group (0.5%), Fusobacteria (0.1%), Spirochaetes (0.02%), Candidatus Latescibacteria (0.02%), DPANN group (0.02%), and Opisthokonta (0.02%)

(8)	Reactive oxygen species metabolism	Catalase, cytochrome c551 peroxidase. Manganese superoxide dismutase, peroxidase, superoxide dismutase [Cu-Zn] precursor, superoxide dismutase [Fe]	Proteobacteria (39%), Euryarchaeota (30%), Unclassified Bacteria (9.6%), Bacteroidetes/Chlorobi group (4.7%), Actinobacteria (2.9%), Firmicutes (2.5%), Gemmatimonadetes (0.9%), Unclassified Archaea (0.4%), Balneolaeota (0.4%), Viridiplantae (0.2%), Cyanobacteria/Melainabacteria group (0.2%), Deinococcus-Thermus (0.1%), Planctomycetes (0.1%), Chloroflexi (0.1%), Opisthokonta (0.1%), Terrabacteria group (0.1%), Verrucomicrobia (0.03%), FCB group (0.03%), Aquificae (0.01%), Candidatus Aegiribacteria (0.01%), Spirochaetes (0.01%), and TACK group (0.01%)

(9)	Proteins regulating cellular response	Aerobic respiration control protein arcA and arcB, DNA protection during starvation protein, manganese superoxide dismutase, Peroxidase, peroxide stress regulator, RNA polymerase sigma factor, redox-sensitive transcriptional activator (SoxR), superoxide dismutase [Cu-Zn], superoxide dismutase [Mn]	Euryarchaeota (36%), Proteobacteria (23.3%), Unclassified Bacteria (8.7%), Firmicutes (10.9%), Bacteroidetes/Chlorobi group (3.5%), Actinobacteria (2.2%), Unclassified Archaea (2%), Bacteria candidate phyla (0.9%), Spirochaetes (0.8%), Terrabacteria group (0.6%), Deinococcus-Thermus (0.5%), Chloroflexi (0.4%), Balneolaeota (0.4%), Tenericutes (0.4%), DPANN group (0.35%), Cyanobacteria/Melainabacteria group (0.2%), Opisthokonta (0.1%), FCB group (0.1%), Thermotogae (0.1%), Elusimicrobia (0.05%), Candidatus Omnitrophica (0.04%), Planctomycetes (0.04%), FCB group candidate division Zixibacteria (0.03%), Viridiplantae (0.03%), Armatimonadetes (0.01%), Chlamydiae (0.01%), Nitrospirae (0.01%), Nitrospinae/Tectomicrobia group (0.01%), FCB group Candidatus Cloacimonetes (0.01%), and Parabasalia (0.01%)

(10)	Regulation of nuclear processes	C-terminal binding protein 2, glyceraldehyde-3-phosphate dehydrogenase (GAPDH), NAD-dependent deacetylase, NAD-dependent glyceraldehyde-3-phosphate dehydrogenase, NAD-dependent protein deacetylase of SIR2 family, NADPH-dependent glyceraldehyde-3-phosphate dehydrogenase, nicotinate phosphoribosyltransferase, poly [ADP-ribose] polymerase-1	Euryarchaeota (30.8%), Proteobacteria (23.3%), Unclassified Bacteria (10.6%), Firmicutes (7.9%), Unclassified Archaea (6.3%), Bacteroidetes/Chlorobi group (2.7%), Actinobacteria (2.6%), Spirochaetes (0.8%), Cyanobacteria/Melainabacteria group (0.5%), Bacteria candidate phyla (0.5%), Balneolaeota (0.5%), Terrabacteria group (0.3%), Planctomycetes (0.24%), TACK group (0.24%), Candidatus Kryptonia (0.2%), Thermotogae (0.2%), Candidatus Cloacimonetes (0.1%), Opisthokonta (0.1%), Synergistetes (0.08%), Viridiplantae (0.08%), Verrucomicrobia (0.05%), Candidatus Marinimicrobia (0.03%), FCB group (0.03%), Tenericutes (0.03%), Deinococcus-Thermus (0.03%), Nitrospinae/Tectomicrobia group (0.03%), and Unclassified Eukaryota (0.03%)

**TABLE 3:** Metagenomic features associated with antibiotic stress response physiology in Sambhar Salt Lake microbiome.

Antibiotic	Identified protein features	Phylogenetic afflation (% abundance)
**Methicillin**	FemC, HmrA protein. Penicillin-binding protein 1A/1B (PBP1), UDP-*N*-acetylmuramoylalanyl-D-glutamate-2,6-diaminopimelate ligase, UDP-*N*-acetylmuramoylalanyl-D-glutamyl-2,6-diaminopimelate–D-alanyl-D-alanine ligase, Undecaprenyl-phosphate *N*-acetylglucosaminyl 1-phosphate transferase	Proteobacteria (20.2%), Firmicutes (18.5%), Unclassified bacteria (16.7%), Bacteroidetes/Chlorobi group (13.7%), Actinobacteria (2.04%), Terrabacteria group (1.8%), Acidobacteria (1%), Bacteria candidate phyla (0.8%), Stramenopiles (0.6%), Cyanobacteria/Melainabacteria group (0.6%), Planctomycetes (0.5%), Balneolaeota (0.3%), Candidatus Kapabacteria (0.2%), Armatimonadetes (0.15%), Verrucomicrobia (0.15%), Chloroflexi (0.12%), Viridiplantae (0.1%), Candidatus Omnitrophica (0.06%), Nitrospinae/Tectomicrobia group (0.06%), Tenericutes (0.06%), Elusimicrobia (0.03%), Nitrospirae (0.03%), Chlamydiae (0.03%), Deinococcus-Thermus (0.03%), and Opisthokonta (0.03%)

**Fluoroquinolones**	DNA gyrase subunit A and subunit B, topoisomerase IV subunit A and subunit B	Euryarchaeota (33.5%), Proteobacteria (29%), Firmicutes (12%), Unclassified Archaea (6.8%), Unclassified Bacteria (5%), Bacteroidetes/Chlorobi group (2.3%), Terrabacteria group (0.7%), Bacteria candidate phyla (0.6%), Actinobacteria (0.5%), Spirochaetes (0.4%), Balneolaeota (0.3%), Tenericutes (0.3%), Planctomycetes (0.3%), Chloroflexi (0.2%), Cyanobacteria/Melainabacteria group (0.2%), Deinococcus-Thermus (0.2%), Thermotogae (0.1%), Deferribacteres (0.1%), Lentisphaerae (0.1%), Fusobacteria (0.1%), Nitrospinae/Tectomicrobia group (0.1%), Chlamydiae (0.1%), Gemmatimonadetes (0.1%), DPANN group (0.1%), Acidobacteria (0.05%), FCB group candidate division Zixibacteria (0.04%), Nitrospirae (0.04%), Fibrobacteres (0.03%), PVC group (0.03%), Opisthokonta (0.03%), Candidatus Omnitrophica (0.03%), Verrucomicrobia (0.03%), Synergistetes (0.02%), FCB group (0.02%), Elusimicrobia (0.02%), FCB group Candidatus Hydrogenedentes (0.01%), Armatimonadetes (0.01%), Rhodophyta (0.01%), Alveolata (0.01%), and others

**Beta lactams**	Beta-lactamase, cephalosporinase, beta-lactamase class C and other penicillin binding proteins, metal-dependent hydrolases of the beta-lactamase superfamily I and superfamily II	Proteobacteria (31.7%), Bacteroidetes/Chlorobi group (12%), Unclassified Bacteria (10.3%), Euryarchaeota (5.6%), Actinobacteria (4.2%), Firmicutes (3.5%), Balneolaeota (2.7%), Terrabacteria group (2.4%), Acidobacteria (1.3%), Cyanobacteria/Melainabacteria group (0.9%), Gemmatimonadetes (0.7%), Planctomycetes (0.6%), FCB group (0.5%), Bacteria candidate phyla (0.3%), Spirochaetes (0.2%), Opisthokonta (0.1%), Deinococcus-Thermus (0.1%), FCB group Candidatus Kryptonia (0.05%)

**Erythromycin**	Dimethyladenosine transferase	Proteobacteria (68.2%), Unclassified Bacteria (8.2%), Bacteroidetes/Chlorobi group (6.8%), Firmicutes (3.2%), Euryarchaeota (3.2%), Bacteria candidate phyla (2.7%), Actinobacteria (1.8%), Terrabacteria group (0.45%), and Stramenopiles (0.45%)

**Fosfomycin**	Fosfomycin resistance protein (FosA)	Unclassified organisms (66.7%) and Firmicutes (33.4%)
**Vancomycin**	Vancomycin B-type resistance protein (VanW), Vancomycin response regulator (VanR), Sensor histidine kinase VanS, Vancomycin B-type resistance protein (VanX)	Firmicutes (61.5%), Unclassified Bacteria (5.3%), Terrabacteria group (2%), Proteobacteria (2%), Actinobacteria (1.4%), and Balneolaeota (1%)

**d-cysteine**	Cystine ABC transporter, ATP-binding protein. Cystine ABC transporter, permease protein. D-cysteine desulfhydrase, L-cystine ABC transporter, periplasmic cystine-binding protein	Firmicutes (27.3%), Proteobacteria (22.7%), Euryarchaeota (22.7%), Unclassified Bacteria (11.4%), and Actinobacteria (9.1%)

**Multidrug resistance efflux pumps**	Acriflavin resistance protein, macrolide export ATP-binding/permease protein (MacB). Macrolide-specific efflux protein (MacA), membrane fusion protein of RND family multidrug efflux pump, multi antimicrobial extrusion protein [Na(+)/drug antiporter], multidrug efflux RND transporter (MexD), multidrug efflux pump component (MtrF). RND efflux system, inner membrane transporter (CmeB), RND efflux system, membrane fusion protein (CmeA)	Proteobacteria (42.1%), Bacteroidetes/Chlorobi group (12.8%), Firmicutes (11%), Unclassified Bacteria (10%), Balneolaeota (2.5%), Euryarchaeota (1%), FCB group (0.5%), FCB group Candidatus Latescibacteria (0.5%), Actinobacteria (0.4%), Cyanobacteria/Melainabacteria group (0.3%), Opisthokonta (0.3%), Spirochaetes (0.2%), Bacteria candidate phyla (0.2%), Armatimonadetes (0.1%), Fusobacteria (0.1%), Acidobacteria (0.1%), Unclassified Archaea (0.1%), Gemmatimonadetes (0.1%), Planctomycetes (0.1%), Terrabacteria group (0.1%), FCB group Candidatus Cloacimonetes (0.1%), Tenericutes (0.1%), Elusimicrobia (0.1%), Chloroflexi (0.05%), Verrucomicrobia (0.05%), Nitrospinae/Tectomicrobia group (0.04%), Nitrospirae (0.04%), Alveolata (0.04%), Fibrobacteres (0.02%), FCB group candidate division Zixibacteria (0.02%), Candidatus Kapabacteria (0.01%), Thermotogae (0.01%), Synergistetes (0.01%), PVC group Candidatus Omnitrophica (0.01%), FCB group Candidatus Aegiribacteria (0.01%), Aquificae (0.01%), Calditrichaeota (0.01%), Chrysiogenetes (0.01%), PVC group (0.01), Haptophyceae (0.01%), and DPANN group (0.01%)

**TABLE 4:** Metagenomic features associated with metal and metalloids stress response physiology in Sambhar Salt Lake microbiome.

Metal and metalloid	Identified protein features	Phylogenetic afflation (% abundance)
**Arsenic**	Arsenical pump-driving ATPase, arsenical-resistance protein ACR3, arsenate reductase, arsenical resistance operon *trans*-acting repressor (ArsD), arsenical resistance operon repressor, arsenic efflux pump protein, arsenic resistance protein (ArsH)	Euryarchaeota (66.2%), Proteobacteria (8.7%), Firmicutes (5.5%), Unclassified Archaea (4%), Unclassified Bacteria (2.9%), Actinobacteria (1.5%), Bacteroidetes/Chlorobi group (0.7%), Chrysiogenetes (0.5%), Acidobacteria (0.4%), Thermotogae (0.2%), Cyanobacteria/Melainabacteria group (0.2%), Verrucomicrobia (0.1%), Terrabacteria group (0.1%), Haptophyceae (0.1%), Planctomycetes (0.1%), Opisthokonta (0.1%), Nitrospirae (0.03%), Chloroflexi (0.03%), Bacteria candidate phyla (0.03%), Viridiplantae (0.02%), Spirochaetes (0.02%), Bacterial viruses (0.02%), Deinococcus-Thermus (0.01%), Haloplasmatales (0.01%), Lentisphaerae (0.01%), Tenericutes (0.01%), Asgard group (0.01%), TACK group (0.004%), and Amoebozoa (0.004%)

**Copper**	CopG protein, copper chaperone, copper resistance protein B&D, copper tolerance protein, copper-binding periplasmic protein, copper-sensing two-component system response regulator (CusR), copper-translocating P-type ATPase, Cu(I)-responsive transcriptional regulator, cytochrome c hemelyase subunit CcmF and CcmH, heavy metal-(Cd/Co/Hg/Pb/Zn)-translocating P-type ATPase, multicopper oxidase, multidrug resistance transporter, (Bcr/CflA), Blue copper oxidase (CueO), copper homeostasis protein CutC, CutE, and CutF, magnesium and cobalt efflux protein (CorC), mprotein, suppressor for copper-sensitivity (ScsB), periplasmic divalent cation tolerance protein (cutA)	Euryarchaeota (34.8%), Proteobacteria (18%), Firmicutes (9.5%), Unclassified Bacteria (7.2%), Bacteroidetes/Chlorobi group (3.9%), Unclassified Archaea (2.9%), Actinobacteria (2.1%), Bacteria candidate phyla (1.4%), Deinococcus-Thermus (0.45%), Cyanobacteria/Melainabacteria group (0.4%), Terrabacteria group (0.35%), Balneolaeota (0.3%), Opisthokonta (0.3%), Chloroflexi (0.3%), Spirochaetes (0.2%), DPANN group (0.2%), Armatimonadetes (0.2%), Viridiplantae (0.1%), Acidobacteria (0.1%), Candidatus Kapabacteria (0.05%), Haloplasmatales (0.03%), Fibrobacteres (0.03%), Planctomycetes (0.03%), Nitrospirae (0.03%), Gemmatimonadetes (0.03%), Stramenopiles (0.03%), Thermotogae (0.02%), Fusobacteria (0.02%), Synergistetes (0.01%), Verrucomicrobia (0.01%), and Chlamydiae (0.01%)

**Cobalt-zinc-cadmium**	Cadmium-transporting ATPase, cation efflux system protein CusA and CusB, Cd(II)/Pb(II)-responsive transcriptional regulator, cobalt-zinc-cadmium resistance protein (CzcD), cobalt/zinc/cadmium efflux RND transporter, membrane fusion protein (CzcB), copper sensory histidine kinase (CusS), DNA-binding heavy metal response regulator, heavy metal RND efflux outer membrane protein (CzcC). Heavy metal resistance transcriptional regulator (HmrR), heavy metal sensor histidine kinase. Probable Co/Zn/Cd efflux system membrane fusion protein, probable cadmium-transporting ATPase, transcriptional regulator, (MerR).	Bacteroidetes/Chlorobi group (34.31%), Bacteroidetes/Chlorobi group (23.9%), Proteobacteria (14%), Euryarchaeota (11.6%), Unclassified bacteria (11.5%), Balneolaeota (7%), Firmicutes (5.8%), Cyanobacteria/Melainabacteria group (1.3%), Planctomycetes (0.7%), FCB group Candidatus Cloacimonetes (0.6%), Bacteria candidate phyla (0.5%), Actinobacteria (0.5%), Gemmatimonadetes (0.5%), FCB group (0.5%), Chloroflexi (0.43%), Spirochaetes (0.4%), Elusimicrobia (0.4%), Nitrospirae (0.3%), Terrabacteria group (0.3%), Lentisphaerae (0.2%), Acidobacteria (0.2%), Viridiplantae (0.2%), Verrucomicrobia (0.2%), Thermodesulfobacteria (0.15%), PVC group Candidatus Omnitrophica (0.1%), Nitrospinae/Tectomicrobia group (0.1%), Unclassified Archaea (0.1%), Opisthokonta (0.1%), Euglenozoa (0.04%), Fusobacteria (0.02%), Thermotogae (0.02%), Chlamydiae (0.015%), FCB group Candidatus Marinimicrobia (0.015%), FCB group Candidatus Kryptonia (0.015%), Deferribacteres (0.015%), Aquificae (0.01%), TACK group (0.01%), and Rhodophyta (0.01%)

**Cadmium**	Cadmium efflux system accessory protein, cadmium-transporting ATPase	Firmicutes (58.5%), Euryarchaeota (17.7%), Unclassified Bacteria (7.2%), Actinobacteria (3%), Proteobacteria (1.7%), Terrabacteria group (1.4%), Cyanobacteria/Melainabacteria group (1.1%), Chloroflexi (0.7%), Bacteroidetes/Chlorobi group (0.7%), Deferribacteres (0.7%), Fusobacteria (0.2%), Asgard group (0.2%), Opisthokonta (0.2%), Thermotogae (0.1%), Tenericutes (0.1%), Planctomycetes (0.1%), and PVC group Candidatus Omnitrophica (0.1%)

**Mercury**	Mercuric ion reductase, PF00070 family, FAD-dependent NAD(P)-disulfide oxidoreductase, mercuric ion reductase, mercuric resistance operon regulatory protein, mercuric transport protein (MerT)	Proteobacteria (48.4%), Unclassified Bacteria (12.9%), Actinobacteria (10.1%), Opisthokonta (4.7%), Alveolata (4.4%), Bacteroidetes/Chlorobi group (3.8%), Euryarchaeota (2.5%), Cyanobacteria/Melainabacteria group (1.9%), Chloroflexi (1.25%), Spirochaetes (0.9%), Terrabacteria group (0.6%), Acidobacteria (0.6%), Firmicutes (0.6%), and Balneolaeota (0.3%)

### Energy Harvesting and Utilization by the Sambhar Salt Lake Microbiome

Saline lake water microbiome was identified to possess metabolic machinery to harvest solar energy either for the generation of energy-rich molecules like ATP and NADH through cyclic and non-cyclic photo-phosphorylation or for the production of carbohydrates through carbon fixation by the Calvin–Benson cycle. Salt lake microbiome showed the abundance of protein features whose homologs have been characterized for electron transport and photophosphorylation (Photosystems I, II, and photosynthetic reaction center), light-harvesting complexes, Bacteriorhodopsin, and Proteorhodopsin (Supplementary Table 7). Carbon fixation was another prominent energy fixation process (0.68%) in salt lake microbes. It includes protein features involved in the CO_2_ uptake through carboxysome (14.6%), CO_2_ fixation through the Calvin–Benson cycle (43.3%), and Photorespiration (oxidative C_2_ cycle) (42%) (Supplementary Table 7). These were probably the major energy influx mechanisms in the salt lake microbiome for successful survival and adaptation in nutrient-deprived harsh environments. Sambhar Salt Lake microbes also enriched themselves with robust machinery to utilize photosynthetic outcomes to meet their energy demands (Supplementary Table 7). The presence of a diverse array of protein features for energy harvesting and utilization defines energy cycling within the microbiome.

### Cellular Interaction and Communication Among Sambhar Salt Lake Microbes

The presence of the quorum sensing-associated protein features within the metagenome indicates a mechanism for microbial communication and biofilm formation. The homologs of these features were characterized for autoinducer-2 synthesis (82.4%), quorum sensing in *Yersinia* (8.89%), symbiotic colonization and sigma-dependent biofilm-forming gene cluster (5.10%), biofilm adhesin biosynthesis (0.83%), autoinducer 2 (AI-2) transport and processing (lsrACDBFGE operon) (1.4%), quorum sensing in *Vibrio* (0.46%), quorum sensing regulation in *Pseudomonas* (0.26%), acyl homoserine Lactone (*AHL*) autoinducer quorum sensing (0.35%), biofilm formation in *Staphylococcus* (0.11%), and protein *YjgK* cluster linked to biofilm formation (0.04%).

### Assessment of the Lake Microbiome Functioning

Metabolomic profiling of the Sambhar Lake microbiome identified 11,265 and 797 statistically significant features (*p* < 0.01) after the analysis of the metabolic profile captured with positive and negative ESI mode (Supplementary Table 8). These differentially abundant metabolic features were mapped onto the metabolic pathways associated with carbohydrate metabolism, nucleotide metabolism, fatty acid synthesis, and energy utilization. Additionally, metabolic features were mapped onto the adaptive pathways to overcome osmotic stress, oxidative stress, antibiotic stress, and metal/metalloid toxicity (Supplementary Table 8). Captured metabolite profile also highlights enrichment of various metabolic pathways (Supplementary Table 9). The presence of these metabolites indicates the functioning of protein features associated with adaptive physiology, as well as microbiome functioning in this hostile environment.

### Comparative Metagenomic Analysis

**FIGURE 2:**
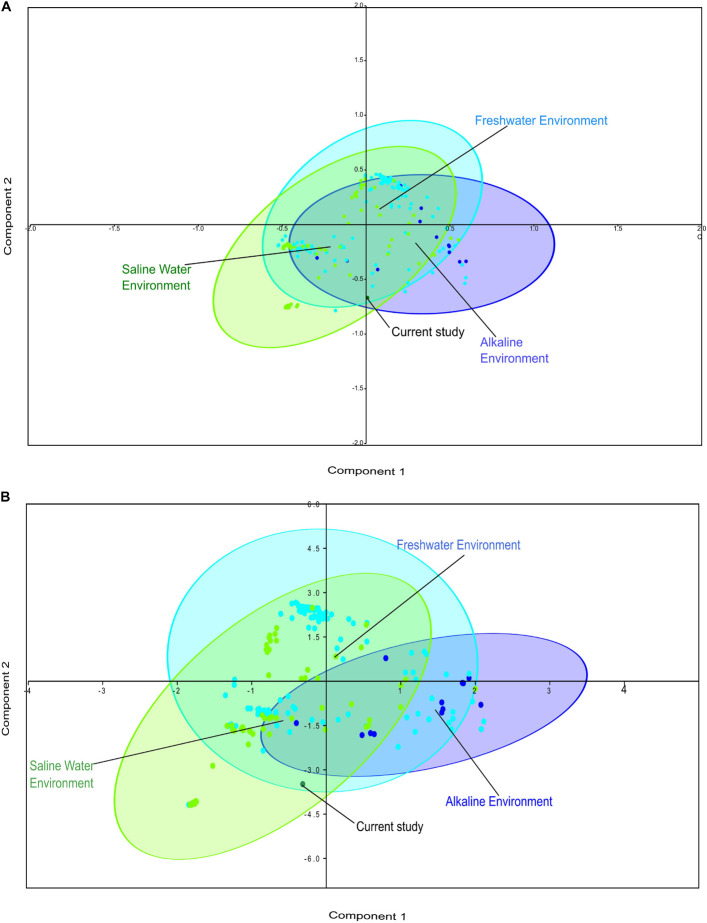
Principal component analysis (PCA) of the diverse metagenomes with rRNA-based taxonomic features. Principal component analysis showing the clustering of the freshwater, alkaline water, and saline water metagenomes with taxonomic features identified at the phylum level of taxonomic hierarchy **(A)** and class level of taxonomic hierarchy **(B)**.

**FIGURE 3:**
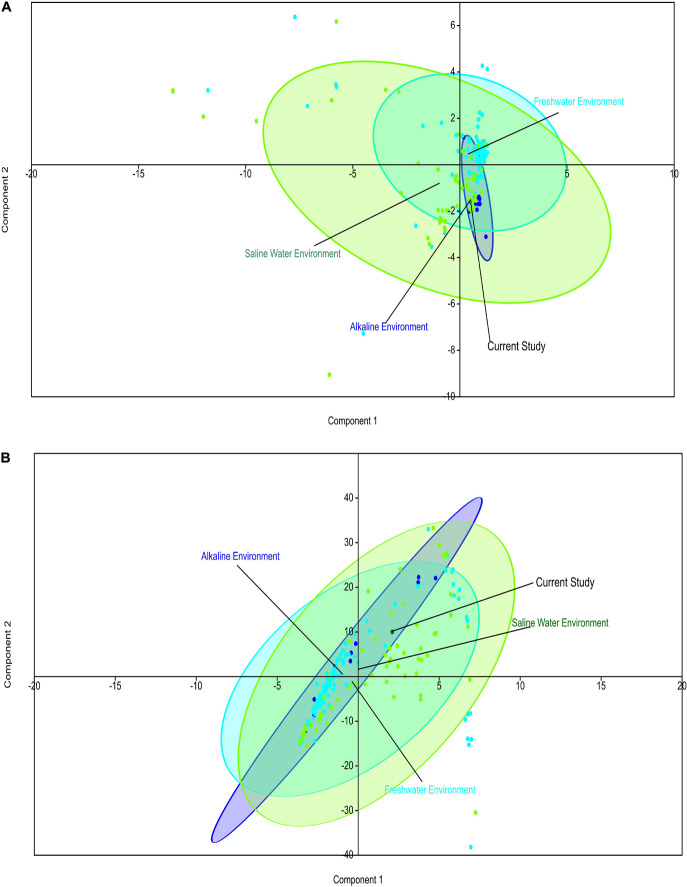
Principal component analysis (PCA) of the diverse metagenomes with subsystem annotated protein features. Principal component analysis showing the clustering of the freshwater, alkaline water, and saline water metagenomes with subsystem database annotated metabolic at the hierarchy level L1 **(A)** and hierarchy level L2 **(B)**.

Comparative metagenomics of diverse metagenomes representing the saline, freshwater, and alkaline ecosystems identified uniqueness and similarities at the taxonomy and microbiome physiological functions level. PCA of the diverse metagenomes based on taxonomic features (phylum and class level of taxonomic hierarchy) (Figures 2A,B), as well as metabolic features annotated with the Subsystem database (hierarchy level L1 and L2) (Figures 3A,B), indicated the differential clustering of the freshwater, alkaline water, and saline water metagenomes (Figures 2A,B). Additionally, the current metagenome sample lay within the centroid of the saline metagenomes.

**FIGURE 4:**
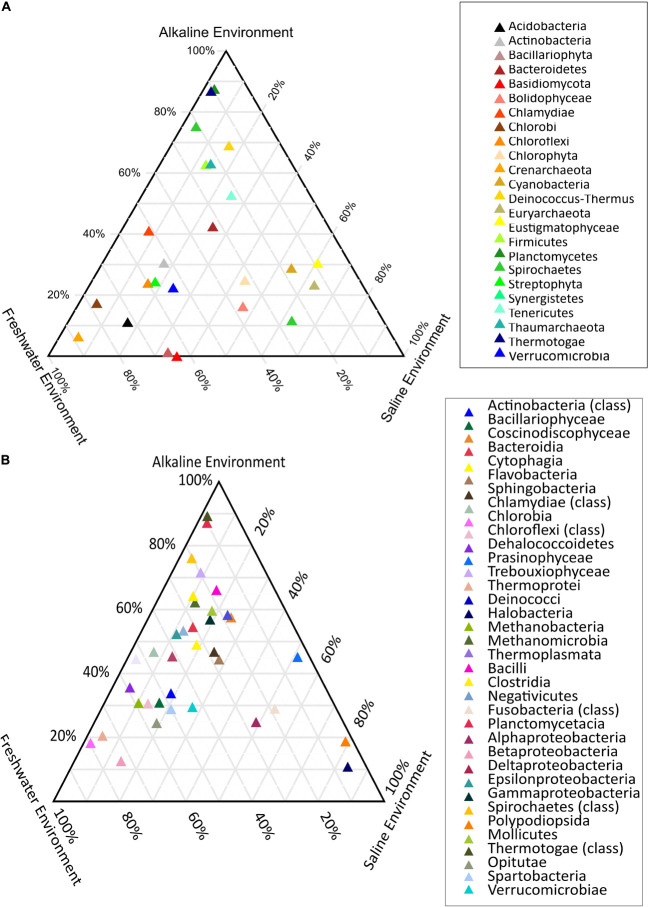
Ternary plots showcasing the differential microbiome composition using rRNA features phylogenetically affiliated at phylum **(A)** and class level **(B)** of the taxonomic hierarchy.

PERMANOVA analysis of the taxonomic (rRNA) features identified in the studied metagenomes indicated that saline, freshwater, and alkaline ecosystems harbor significantly different microbial members (*p* < 0.01). Phylogenetic affiliation of the rRNA features at the phylum level of taxonomic classification and their PERMANOVA analysis indicated that saline microbiome composition was significantly different from freshwater (*p* = 0.0003), as well as from the alkaline environment (*p* = 0.0024) (Supplementary Table 10). Even the microbiome composition of the alkaline ecosystems was significantly different (*p* = 0.0084) from the freshwater ecosystems (Supplementary Table 10). PERMANOVA analysis with rRNA features classified at the class level taxonomy has made similar observations (Supplementary Table 11). A statistically significant difference (*p* < 0.01) was observed in the microbiome composition of the studied ecosystems (Supplementary Table 11). An attempt was made to showcase microbiome compositional differences among the studied ecosystems using the ternary plots with phylogenetically affiliated rRNA features at phylum and class level of taxonomic hierarchy (Figures 4A,B). The plot showed an abundance (>70%) of Thermotogae, Planctomyces, and Spirochetes among the alkaline ecosystem, while Euryarchaeota, *Eustigmatophyceae*, and Cyanobacteria were abundant in the saline ecosystem (Figure 4A). Crenarchaeota, Chlorobi, and Acidobacteria were selectively abundant in the freshwater ecosystem (Figure 4A). Ternary plots with phylogenetically affiliated rRNA features at the class level of taxonomic hierarchy indicated that Thermotogae, Planctomycetia, Spirochetes, and Trebouxiophyceae were abundant (>70%) in the alkaline metagenomes, while Halobacteria, Fusobacteria, Coscinodiscophyceae, and Deltaproteobacteria showed an abundance within the saline ecosystem. Chlorobia, Chloroflexi, and Betaproteobacteria were selectively abundant in the freshwater ecosystem (Figure 4B). Identified saline ecosystems-specific microbes represented similar microbial groups identified with rRNA feature and protein feature-based phylogenetic characterization of Sambhar Salt Lake microbiome.

Phylogenetic affiliation of the protein features at the class level of taxonomic classification and their PERMANOVA analysis indicated that microbiome composition of all studied ecosystems (saline, freshwater, and alkaline environment) was significantly different (*p* = 0.0003) from each other (Supplementary Table 12). Phylogenetic affiliation of the protein features at the phylum level of taxonomic classification and their PERMANOVA analysis indicated that alkaline microbiome composition was significantly different from the freshwater environment (*p* = 0.009), as well as from the saline environment (*p* = 0.0003) (Supplementary Table 12). Surprisingly, the microbiome composition of the freshwater ecosystems was not statistically different (*p* = 0.258) from the saline ecosystems (Supplementary Table 12). An attempt was made to showcase microbiome compositional differences among the studied ecosystems using the Ternary plots with phylogenetically affiliated protein features at phylum and class level of taxonomic hierarchy (Supplementary Figures 3, 4). The plot showed an abundance (>70%) of Thermotogae, Tenericutes, and Bacillariophyta among the alkaline ecosystem, however, no such differentially abundant microbial phyla were observed for freshwater and salt environment (Supplementary Figure 3). Ternary plots with phylogenetically affiliated protein features at the class level of taxonomic hierarchy indicated that Thermotogae, Planctomycetia, and Sordariomycetes, were abundant (>70%) in the alkaline metagenomes, while Deinococci, Deltaproteobacteria, and Dehalococcoidetes showed abundance within the saline ecosystem. Alphaproteobacteria, Betaproteobacteria, and Epsilonproteobacteria were selectively abundant in the freshwater ecosystem (Supplementary Figure 4). These results strengthen our view about ecosystem-specific microbiome enrichment.

**FIGURE 5:**
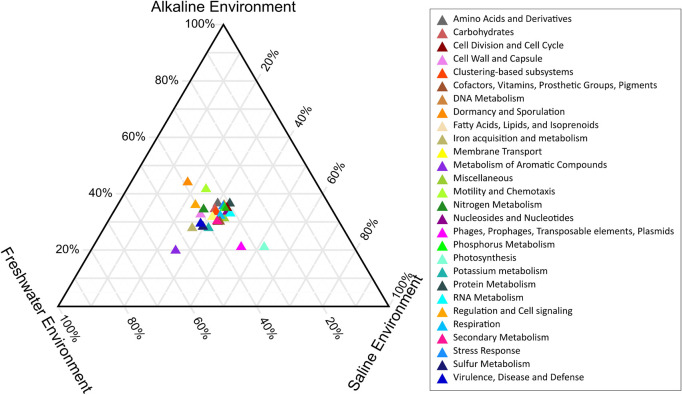
Ternary plot indicating the differentially abundant metabolic features of saline, freshwater, and alkaline ecosystem identified with subsystem annotated metabolic features at hierarchy level 1.

**FIGURE 6:**
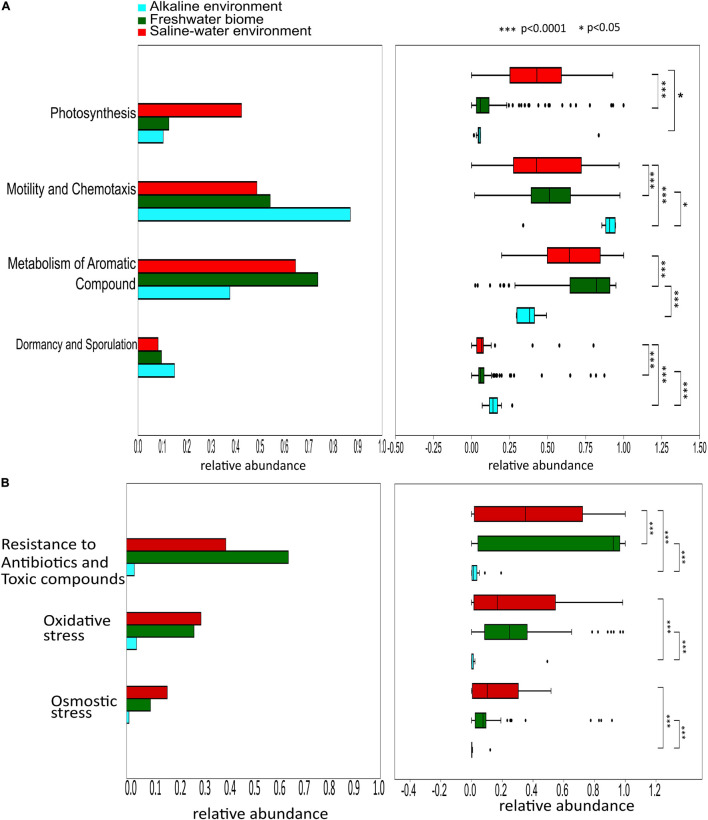
Differential abundance of the protein features associated Dormancy and Sporulation, Motility and Chemotaxis, Metabolism of aromatic compounds, and Photosynthesis (*p* < 0.05) among saline, freshwater, and alkaline metagenomes **(A)**. Comparative analysis shows metabolic features associated with the protection from osmotic stress, oxidative stress, antibiotic, and metal/metalloid toxicity among saline, freshwater, and alkaline ecosystems indicating the significantly diverse abundance (*p* < 0.05) of these metabolic features in these ecosystems **(B)**.

PERMANOVA analysis of the Subsystem annotated protein features of the saline, freshwater, and alkaline ecosystem metagenomes and indicated the presence of unique metabolic functionaries (*p* < 0.01). Clustering of the subsystem annotated metabolic feature at hierarchy level 1 and their PERMANOVA analysis indicated that saline metagenome was significantly different from freshwater (*p* = 0.0006), while no statistically significant difference was observed compared to the alkaline environment (*p* = 0.4869) (Supplementary Table 13). Surprisingly, alkaline and freshwater ecosystems seem to possess variable but not statistically significant (*p* = 0.3666) diverse metabolic functionaries (Supplementary Table 13). PERMANOVA analysis with subsystem annotated and clustered metabolic features at hierarchy level 2 made similar observations (Supplementary Table 14). A statistically significant difference (*p* = 0.048) was observed in the microbiome composition of the saline and freshwater ecosystems (Supplementary Table 14). Differentially abundant protein features of the saline, freshwater, and alkaline ecosystem were identified with ternary plots using the Subsystem annotated protein features at hierarchy level 1 (Figure 5). Protein features associated with osmotic stress, oxidative stress, resistance to antibiotics and toxic compounds, dormancy and sporulation, metabolism of aromatic compounds, and photosynthesis indicated a differential abundance (Supplementary Figures 5, 6). Even features associated with Dormancy and Sporulation, Motility and Chemotaxis, Metabolism of aromatic compounds, and Photosynthesis were found significantly different (*p* < 0.05) among these metagenomes (Figure 6A and Supplementary Table 15). Sambhar Salt Lake metagenome possesses several metabolic features associated with protection from osmotic stress, oxidative stress, antibiotic, and metal/metalloid toxicity (Tables 1–4). These features might be essential for microbial survivability in these stressed environments. Comparative analysis indicated the significantly diverse abundance (*p* < 0.01) of these protein features in these ecosystems (Figure 6B). Metabolic features associated with osmotic stress, oxidative stress, and photosynthesis were significantly abundant (*p* < 0.01) in the saline metagenomes, while Dormancy and Sporulation, Motility, and Chemotaxis-related metabolic features were significantly abundant (*p* < 0.01) in alkaline metagenomes (Figure 6A). Freshwater metagenome showed a significant abundance (*p* < 0.01) of metabolic features associated with the Metabolism of aromatic compounds, antibiotic, and metal/metalloid toxicity (Figure 6B). The presence of diverse microbiome composition and enrichment of differential metabolic functionaries in these ecosystems indicates environment-specific microbiome evolution.

## Discussion

Sambhar Lake is one of the largest semi-arid, temperate, and hypersaline inland ecosystems, making it a hostile environment for the growth of any life form (Yadav and Sarin, 2009). Survival of any living form in such a hostile environment possibly requires exhaustive physiological machinery to cope up with the various abiotic stresses (osmotic stress, metabolic oxidative stress, metal stress, heat and cold stress, and acid stress) and to meet energy demands (Chen et al., 2015). Microbes are one of the most robust life forms identified from all possible habitats on earth (Rampelotto, 2013), as well as in space (Fierer, 2008). Their possible habitats include harsh environments {acid mine drainage (Chen et al., 2015), hydrothermal vents (Anantharaman et al., 2016), space station (Mora et al., 2016), and facile environments [pond water (Kapardar et al., 2010), human gut (Chauhan et al., 2018), human lung (Gupta et al., 2021), and soil (Ahmed et al., 2018)]}. It seems possible due to their genetic flexibility to acquire novel genetic features to cope up with the physiological demands of the respective habitats. Likewise, Sambhar Lake is the habitat of many microbes [Eubacteria (Sangeeta et al., 2016), Archaea (Upasani, 2008), and Alga (Arun and Singh, 2014)]. Several halotolerant microbes were isolated (Gaur et al., 2015) and being employed for various biotechnological potentials (Singh and Jha, 2016). Most of these culture-based studies were primarily focused on the isolation of microbial cultures (Bhatt et al., 2016), while a few were focused on assessing its microbial diversity (Sharma et al., 2013). These studies were limited in their scope and were unable to answer basic questions related to microbiome adaptation, survival, and functioning in such an extreme environment. Hypersaline, semi-arid environment is a unique ecosystem, possibly very few around the globe (Brauner et al., 2013). The biotechnological and physiological potential associated with the hypersaline microbiome has attracted the attention of researchers around the globe (Enache et al., 2017). However, a majority of these studies were primarily focused on determining the phylogenetic affiliation of the residing microbes (Naghoni et al., 2017) and provide very limited information about the microbiome functioning (Sorokin and Kuenen, 2005). Metagenomics allows direct access to the genetic content of the microbiome to describe its composition and physiological functions (Ahmed et al., 2018; Yadav et al., 2021). Therefore, metagenomics was used to study the Sambhar Lake microbiome’s adaptation, survival, and physiological functions in such a harsh environment.

The rRNA features of the Sambhar Salt Lake metagenome described the predominance of Euryarchaeota, Firmicutes, Proteobacteria, Bacteroidetes, Actinobacteria, Cyanobacteria, and Chlorophyta lineages (Supplementary Tables 3, 4). Some of these identified microbial members were established as either halophilic or halotolerant (Sorokin and Kuenen, 2005; Upasani, 2008; Brauner et al., 2013; Arun and Singh, 2014; Sangeeta et al., 2016; Enache et al., 2017; Naghoni et al., 2017). The presence of such rRNA features in Sambhar Salt Lake is in synchronization with the earlier diversity studies (Sangeeta et al., 2016). The presence of the halophilic microorganisms in saline environments is well expected and similar phylotypes were recorded from various other saline environments (Naghoni et al., 2017). Additionally, comparative metagenomics indicates Halobacteria as the signature microbial group of the saline ecosystems. A correct representation of Sambhar Lake phylotypes is a must to develop an actual image of microbiome functioning (Reigstad and Kashyap, 2013). Accordingly, phylogenetic reconstruction of the lake microbiome was also performed with identified protein features. Phylogenetic assessment of protein features synchronously indicated the predominance of similar phylotypes describing the true representation of Sambhar Salt Lake microbiome genetic elements in the sequence metagenome dataset (Supplementary Table 4).

The metagenomic dataset has 6.95% of the total protein features that were associated with stress tolerance, virulence, sunlight harvesting, and cellular communications (Supplementary Table 5). Among the stress response protein features, osmotolerance, oxidative stress response, metal/metalloid stress response, and heat and cold response were predominant. The osmotolerance features were involved in biosynthesis and uptake of various osmolytes (glycine betaine, proline, ectoine, and hydroxyectoine), membrane transport (aquaporins and ABC transporters), and periplasmic proteins (Table 1 and Figure 1). These are well-described osmotolerance mechanisms identified within halophilic and halotolerant microbes (Das et al., 2015). The presence of these features in almost all microbial clades of the lake microbiome describes osmotic adaptability as an imperative parameter for successful survival in the hypersaline environment (Sorokin and Kuenen, 2005). Comparative metagenomics also decoded significant enrichment of osmotic stress tolerance protein features in the saline ecosystem (Figure 6B). As the acquisition of salt-tolerant mechanisms is essential for survival in these hostile environments (Ahmed et al., 2018), the saline ecosystem microbiomes might have enriched themselves with these features as a survival strategy.

Salt is a well-known factor to induce cytosolic oxidative stress and microbes have to counter it for their survival (Hasanuzzaman et al., 2020). This explains the presence of the diverse oxidative response features in almost every microbial group of the lake microbiome (Table 2). These features might allow them to counter metabolic oxidative stress-induced either by excess salt or some other physiological process (Hagemann, 2011). In addition to salts, Sambhar Lake has depositions of toxic metals/metalloids like copper, cadmium, arsenic, chromium, mercury, and zinc (Cherekar and Pathak, 2016). The presence of toxic metal resistance features against these metals/metalloids in the metagenome describes their successful survival and adaptation (Table 4). This is a semi-arid lake with annual rains, a drought cycle (summers), and huge temperature variability like chilling (4–8°C in winters and 40–45°C in summers) (Pathak and Cherekar, 2015). The microbes surviving in such an environment have to cope up with these unavoidable changes. Possibly due to this evolutionary pressure, salt lake microbes might have equipped themselves with temperature variations and desiccation response features (Supplementary Table 6). Additionally, an abundance of antibiotic resistance features in the lake metagenome was a surprise element (Table 3). Microbes are known to synthesize antibiotics to gain an advantage over their neighbors for the utilization of nutrients and growth advantage (Benveniste and Davies, 1973). To challenge the competitive advantage of antibiotic producers, native microbes have developed antibiotic resistance machinery (McEachran et al., 2015). Hereby, several antibiotic resistance genetic reservoirs have been identified from different environments (Fang et al., 2014). This could be a possible explanation for the presence of antibiotic resistance features in the Sambhar Lake metagenome.

As an overview, the abundance of biotic and abiotic stress response features in the Sambhar Lake metagenome decodes their survival strategy in the harsh environment, but the question about the mode of energy generation and channelization is yet to be answered. The presence of photosynthetic features for photophosphorylation, bacteriorhodopsin, proteorhodopsin, and carbon fixation highlights the source of energy for microbiome functioning (Supplementary Table 7). Sunlight harvesting with proteorhodopsin and bacteriorhodopsin was considered as the main route of energy channelization for microbiome functioning in the various nutrient-deprived ecosystems such as glaciers (Achberger et al., 2017) and sea (DeLong and Beja, 2010). Protein features for CO_2_ uptake and fixation indicated the utilization of photophosphorylation-derived energy to synthesize carbohydrates in the salt lake microbiome. Later on, these carbohydrates can be utilized by the lake microbes using protein features for carbohydrate metabolism. A similar process of energy harvesting and channelization by microorganisms has been established in the isolated studies (Chen et al., 2015). In addition to the abovementioned feature, several protein features were identified especially for the quorum sensing and chemotaxis with a possible role in the microbial communication and growth of biofilms, as described by various studies (Waters and Bassler, 2005). Merely the presence of protein features could not confirm their functions, hereby a translation approach like metabolomics could be used to decode functional metabolic pathways by metabolite profiling (Yadav et al., 2020, 2021). Identification of metabolites associated with adaptive physiology, as well as with general cell physiology confirms their functions in the lake microbiome (Supplementary Table 8).

Although the identified adaptive physiology is essential to survive in this hypersaline environment, are these adaptive physiology-associated protein features specific to the saline ecosystem? or invariably present in all aqueous microbiomes? It could only be assessed through comparative metagenome analysis of saline, alkaline, and freshwater ecosystem microbiomes. The comparative metagenomics indicated a unique microbiome composition and metagenome features in each ecosystem (Supplementary Tables 10–12). The results indicated a differential abundance of stress (oxidative and osmotic) response and energy harvesting features to the saline ecosystem microbiome, while several other features were associated with other ecosystem microbiomes (Figure 6 and Supplementary Table 15). Selective enrichment of specific microbial groups and metabolic features indicated environment-specific microbiome evolution to meet up the challenge posed by the environmental conditions. Holistically, the present study generates a model system to understand microbiome functioning in harsh terrestrial and extraterrestrial environments, as well as indicate the process of environment-specific microbiome evolution.

## Data Availability Statement

Sequence data generated in this study have been deposited at NCBI with an SRA submission ID SUB9945552 and Bio project accession ID PRJNA743411.

## Author Contributions

NC designed the study and experiments. NC, MY, and RP wrote the manuscript. MY, VA, and KG carried out the experiments. NC, MY, and PM analyzed the data. All authors edited the manuscript and approved the final draft of the manuscript.

## Conflict of Interest

The authors declare that the research was conducted in the absence of any commercial or financial relationships that could be construed as a potential conflict of interest.

## Publisher’s Note

All claims expressed in this article are solely those of the authors and do not necessarily represent those of their affiliated organizations, or those of the publisher, the editors and the reviewers. Any product that may be evaluated in this article, or claim that may be made by its manufacturer, is not guaranteed or endorsed by the publisher.

## Footnotes

1
https://www.mg-rast.org/

2
https://www.ternaryplot.com/
