# Supplementary material for: Culture-Independent Exploration of the Hypersaline Ecosystem Indicates the Environment-Specific Microbiome Evolution
Source: Front Microbiol. 2021 Oct 28;12:686549. doi: 10.3389/fmicb.2021.686549 (PMC8581802; doi:10.3389/fmicb.2021.686549)

Culture-independent exploration of the hypersaline ecosystem indicates the environment-specific microbiome evolution

Priyanka Mehta[2†], Monika Yadav[1†], Vasim Ahmed[1†], Khushboo Goyal[1], Rajesh Pandey[2#], and Nar Singh Chauhan[1*]

[1]Department of Biochemistry, Maharshi Dayanand University, Rohtak, Haryana, India

[2] Genomics and Molecular Medicine, INtegrative GENomics of HOst-PathogEn (INGEN-HOPE) laboratory, CSIR-Institute of Genomics and Integrative Biology (CSIR-IGIB), Delhi-110007, India.

[†] These authors contributed equally to this work.

*Corresponding author
Nar Singh Chauhan (nschauhan@mdurohtak.ac.in)

#Co- Corresponding author
Rajesh Pandey (rajeshp@igib.res.in)

Running Title: Muti-omics exploration of saline lake microbiome

Number of Words: 10404

Number of Figures: 6

Number of Tables: 4

(A) Supplementary method

Supplementary method SM1: Metagenomic DNA isolation

A 200mg of the pellet was suspended in 1.45 ml of lysis buffer containing 1.5 M of NaCl, 100 mM of ethylenediaminetetraacetic acid (EDTA), 100 mM of Na_3PO_4, 1% (w/v) cetyl trimethyl ammonium bromide (CTAB), and 100 mM of Tris-HCl (pH 8.0). Proteinase K (1 mg/g of the pellet) was added to the mixture followed by incubation at 37 °C for 1 h with constant shaking (50rpm). The reaction mixture was incubated again at 65°C for 2 h after adding SDS to a final concentration of 2%. An equal volume chloroform and isoamyl alcohol (24:1) was added to the lysate followed by its centrifugation at 8000g for 10 min at ambient room temperature. A 0.6 V (v/v) of isopropyl alcohol was added to the aqueous phase to precipitate the DNA. The DNA was pelleted by centrifugation at 10,000g for 20 min and washed with 1 ml of 70% ethanol. The pellet was air-dried and dissolved in 100µl of Tris-EDTA buffer, pH 8.0. DNA was further purified on 0.7% low melting agarose and high molecular weight DNA was collected (Ahmed et al., 2018).

Supplementary method SM2: Assembly and Binning

The filtered fastq files were used for de novo assembly profiling. The sequences were first assembled into contigs using default settings of the MegaHIT DNA assembler with default k-mer step size of 10 and a k-list of [21,29,39,59,79,99,119,141] was used (Li et al., 2015). The assembly statistics are available in Table SM1. Figure SM1 shows the cumulative length of the assembled contigs.

Table SM1: Metagenomic data assembly statistics

Assembler	contigs (>= 0 bp)	contigs (>= 1000 bp)	contigs (>= 10000 bp)	contigs (>= 50000 bp)	GC (%)	N50 (bp)	L50 (bp)
Megahit	223067	40941	661	6	55.52	1583	20768

Figure SM1: Cumulative length of the assembled contigs.

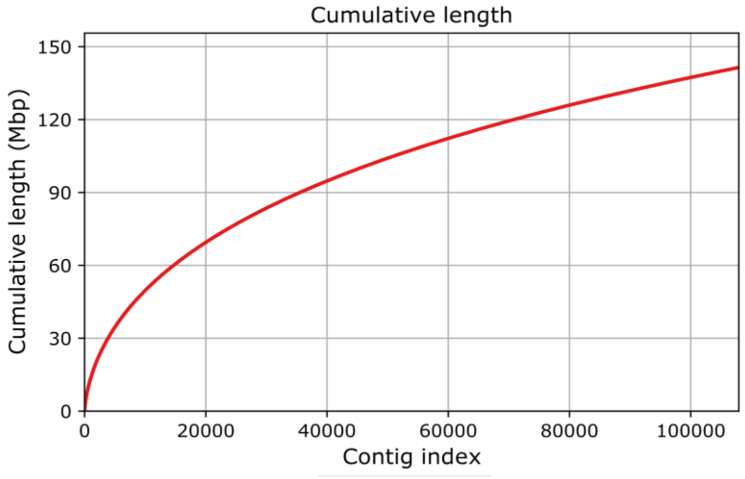

(B) Supplementary Tables

Supplementary Table S1. Statistics of the current metagenome dataset.

Metagenome dataset Characteristic	Value
**Total bp Count**	6,025,375,597 bp
**Average read length**	108 ± 27 bp
**Total Sequences Count**	55,718,072
**Mean GC percent**	58 ± 11 %
**Artificial Duplicate Reads: Sequence Count**	35,158,006
**Post QC: bp Count**	3,812,467,923 bp
**Post QC: Sequences Count**	35,158,006
**Post QC: Mean GC percent**	57 ± 11 %
**Processed: Predicted Protein Features**	17,592,824
**Processed: Predicted rRNA Features**	1,678,528
**Alignment: Identified Protein Features**	4,219,520
**Alignment: Identified rRNA Features**	7,034
**Annotation: Identified Functional Categories**	3,025,041

Supplementary Table S2. Details of the metagenome datasets used for comparative analysis.

MG Rast Accession ID	Nature of Dataset	Origin of the Metagenomic Dataset	Salt Content (%)	pH	Temperature (°C)
mgp12299	Saline	Mediterranean sea biome	38.6	7.8934 ± 0.0076	15.9
mgp13279	Freshwater	Lake Soyang	0.05	NA	25.64
mgp17799	Freshwater	Nanjing,Jiangsu, China	NA	6.4325	18.6
mgp17799	Freshwater	Nanjing,Jiangsu, China	NA	7.97425	16.575
mgp18165	Saline	Mediterranean Sea (SOLA station)	38.49	8.044	14.98 ± 2.33
mgp18165	Saline	Mediterranean Sea (SOLA station)	38.49	8.044	14.98 ± 2.33
mgp18165	Saline	Mediterranean Sea (SOLA station)	38.49	8.044	14.98 ± 2.33
mgp18165	Saline	Mediterranean Sea (SOLA station)	38.49	8.044	14.98 ± 2.33
mgp18165	Saline	Mediterranean Sea (SOLA station)	38.49	8.044	14.98 ± 2.33
mgp18165	Saline	Mediterranean Sea (SOLA station)	38.49	8.044	14.98 ± 2.33
mgp18165	Saline	Mediterranean Sea (SOLA station)	38.49	8.044	14.98 ± 2.33
mgp18165	Saline	Mediterranean Sea (SOLA station)	38.49	8.044	14.98 ± 2.33
mgp18165	Saline	Mediterranean Sea (SOLA station)	38.49	8.044	14.98 ± 2.33
mgp18165	Saline	Mediterranean Sea (SOLA station)	38.49	8.044	14.98 ± 2.33
mgp18165	Saline	Mediterranean Sea (SOLA station)	38.49	8.044	14.98 ± 2.33
mgp18165	Saline	Mediterranean Sea (SOLA station)	38.49	8.044	14.98 ± 2.33
mgp18455	Saline	Adriatic Sea, Mediterranean Sea	38-39	7.845-7.946	20.821
mgp18667	Freshwater	Geneva, Switzerland	NA	NA	14.2
mgp19215	Freshwater	Curua Una River, Brazil	NA	7.0 ± 0.8	28.5
mgp19358	Freshwater	Yangtze Estuary, Shanghai, China	0.07	7.9	30
mgp19358	Freshwater	Yangtze Estuary, Shanghai, China	0.07	7.9	30
mgp19358	Freshwater	Yangtze Estuary, Shanghai, China	0.07	7.9	30
mgp19367	Freshwater	Bagsvaerd, Denmark	NA	6.5±0.1	20
mgp19396	Freshwater	Geneva, Switzerland	NA	6.5±0.5	14.2
mgp19525	Freshwater	Grosser Stechlinsee, Germany	0.3	8.2	22.5
mgp19525	Freshwater	Grosser Stechlinsee, Germany	0.3	8.2	22.5
mgp19525	Freshwater	Grosser Stechlinsee, Germany	0.3	8.2	22.5
mgp21214	Freshwater	Iowa, USA	NA	8.2	2.05
mgp21252	Freshwater	Iowa, USA	NA	8.2	2
mgp21252	Freshwater	Iowa, USA	NA	8.2	2
mgp80824	Freshwater	Blagdon	NA	7.5-7.9	25
mgp80918	Alkaline	Chicago, USA	0.6	13	18.7
mgp83402	Freshwater	Ngenda, Kenya	NA	7.2-8.6	11.8
mgp83414	Freshwater	Karimoni, Kenya	NA	7.12-8.97	17.2
mgp83469	Freshwater	KAITHE, Kenya	0.15 ± 0.06	7.18-8.88	21.4
mgp83498	Freshwater	Ntima	0.25	NA	18.3
mgp83507	Freshwater	Municipality, Kenya	NA	6.5-7.5	21.58-23.05

mgp83543	Freshwater	Kianyau	0.3	8	27
mgp83614	Freshwater	Viçosa, MG	NA	4.3	16.6
mgp83614	Freshwater	Viçosa, MG	NA	4.3	16.6
mgp83635	Saline	Mid Atlantic	35-36.7	7.9	7
mgp83635	Saline	Mid Atlantic	35-36.7	7.9	7
mgp83635	Saline	Mid Atlantic	35-36.7	7.9	7
mgp83635	Saline	Mid Atlantic	35-36.7	7.9	7
mgp83635	Saline	Mid Atlantic	35-36.7	7.9	7
mgp83635	Saline	Mid Atlantic	35-36.7	7.9	7
mgp83635	Saline	Mid Atlantic	35-36.7	7.9	7
mgp83635	Saline	Mid Atlantic	35-36.7	7.9	7
mgp83716	Freshwater	Sensee canal_Ferin, France	NA	NA	14.7
mgp83716	Freshwater	Sensee canal_Ferin, France	NA	NA	14.7
mgp83716	Freshwater	Sensee canal_Ferin, France	NA	NA	14.7
mgp83716	Freshwater	Deûle River_Noyelles-Godault	NA	6.5-6.6	4
mgp83830	Freshwater	Everglades, FL	NA	6.6	4
mgp83830	Freshwater	Everglades, FL	NA	6.6	4
mgp83830	Freshwater	Everglades, FL	NA	6.6	4
mgp83830	Freshwater	Everglades, FL	NA	6.6	4
mgp83830	Freshwater	Everglades, FL	NA	6.6	4
mgp83830	Freshwater	Everglades, FL	NA	6.6	4
mgp83830	Freshwater	Everglades, FL	NA	6.6	4
mgp83830	Freshwater	Everglades, FL	NA	6.6	4
mgp83830	Freshwater	Everglades, FL	NA	6.6	4
mgp83830	Freshwater	Everglades, FL	NA	6.6	4
mgp83830	Freshwater	Everglades, FL	NA	6.6	4
mgp83830	Freshwater	Everglades, FL	NA	6.6	4
mgp83830	Freshwater	Everglades, FL	NA	6.6	4
mgp83830	Freshwater	Everglades, FL	NA	6.6	4
mgp83830	Freshwater	Everglades, FL	NA	6.6	4
mgp83830	Freshwater	Everglades, FL	NA	6.6	4
mgp83830	Freshwater	Everglades, FL	NA	6.6	4
mgp83830	Freshwater	Everglades, FL	NA	6.6	4
mgp83830	Freshwater	Everglades, FL	NA	6.6	4
mgp83830	Freshwater	Everglades, FL	NA	6.6	4
mgp83933	Freshwater	Kazan, Russia	NA	NA	23
mgp83955	Saline	Kulunda_steppe	85	7.63	0
mgp83955	Saline	Lake_tyrrel	25	4	20
mgp83955	Saline	Kulunda_steppe	85	7.63	0
mgp83955	Saline	Kutch_Desert	30.79	7.1-8.6	26.5
mgp83955	Saline	Lake_tyrrel	25	4	20

mgp83955	Saline	Lake_tyrrel	25	4	20
mgp83955	Saline	Isla_Cristina	21	8.2	11.6
mgp83955	Saline	Kutch_Desert	30.79	7.1-8.6	26.5
mgp83955	Saline	Kutch_Desert	30.79	7.1-8.6	26.5
mgp83955	Saline	Santa_Pola	34.2	7.45	14.8
mgp83955	Saline	Kutch_Desert	30.79	7.1-8.6	26.5
mgp83955	Saline	Deep_lake	27	6	0
mgp83955	Saline	Lake_tyrrel	25	4	20
mgp83955	Saline	Lake_tyrrel	25	4	20
mgp83955	Saline	Kutch_Desert	30.79	7.1-8.6	26.5
mgp83955	Saline	Kulunda_steppe	85	7.63	0
mgp83955	Saline	Lake_tyrrel	25	4	20
mgp83955	Saline	Albufera	38	7.69	16.4
mgp83955	Saline	Lake_tyrrel	25	4	20
mgp83955	Saline	Kulunda_steppe	85	7.63	0
mgp83955	Saline	British_Columbia	32	8.1	40
mgp83955	Saline	Lake_tyrrel	25	4	20
mgp83955	Saline	Mar_menor	38-51	8.4	16
mgp83955	Saline	Lost_hammer	25	5.96	0
mgp83955	Saline	Kulunda_steppe	85	7.63	0
mgp84231	Freshwater	Lake Taihu	0.015	8	3
mgp84493	Freshwater	St. Francis Bay	0.35	8	21.5
mgp84507	Freshwater	St. Francis Bay	0.35	8	21.5
mgp84911	Freshwater	Polyu CEE reactor	NA	8.2-8.8	23
mgp85286	Alkaline	Lonar	0.63	10.3	28
mgp85286	Alkaline	Lonar	0.63	10.3	28
mgp85286	Alkaline	Lonar	0.63	10.3	28
mgp85920	Freshwater	Shanghai, China	NA	6.5	25-32
mgp85920	Freshwater	Shanghai, China	NA	6.5	25-32
mgp85920	Freshwater	Shanghai, China	NA	6.5	25-32
mgp85945	Freshwater	Gillbach	NA	8.01-8.46	21
mgp85945	Freshwater	Gillbach	NA	8.01-8.47	21
mgp85945	Freshwater	Gillbach	NA	8.01-8.48	21
mgp85945	Freshwater	Gillbach	NA	8.01-8.49	21
mgp85945	Freshwater	Gillbach	NA	8.01-8.50	21
mgp85945	Freshwater	Gillbach	NA	8.01-8.51	21
mgp85945	Freshwater	Gillbach	NA	8.01-8.52	21
mgp86095	Freshwater	Gillbach	NA	8.01-8.53	21
mgp86095	Freshwater	Gillbach	NA	8.01-8.54	21
mgp86095	Freshwater	Gillbach	NA	8.01-8.55	21
mgp86098	Freshwater	Gillbach	NA	8.01-8.56	21

mgp86214	Freshwater	Cape Recife	NA	8-8.4	14
mgp86214	Freshwater	Cape Recife	NA	8-8.4	14
mgp86214	Freshwater	Cape Recife	NA	8-8.4	14
mgp86972	Freshwater	Lake Erie	0.05-0.6	8-8.2	9.5-14.6
mgp87175	Alkaline	Lonar	0.63	10.3	28
mgp87175	Alkaline	Lonar	0.63	10.3	28
mgp88711	Freshwater	Trunk River, MA	NA	7	7.8
mgp88711	Freshwater	Trunk River, MA	NA	7	7.8
mgp89658	Freshwater	Everglades, FL	NA	6.6	31
mgp89658	Freshwater	Everglades, FL	NA	6.6	31
mgp90129	Freshwater	Fujian	NA	NA	28-30
mgp90129	Freshwater	Fujian	NA	NA	28-30
mgp90129	Freshwater	Fujian	NA	NA	28-30
mgp90129	Freshwater	Fujian	NA	NA	28-30
mgp90129	Freshwater	Fujian	NA	NA	28-30
mgp90129	Freshwater	Fujian	NA	NA	28-30
mgp90129	Freshwater	Fujian	NA	NA	28-30
mgp90129	Freshwater	Fujian	NA	NA	28-30
mgp90129	Freshwater	Fujian	NA	NA	28-30
mgp90129	Freshwater	Fujian	NA	NA	28-30
mgp90129	Freshwater	Fujian	NA	NA	28-30
mgp90129	Freshwater	Fujian	NA	NA	28-30
mgp90129	Freshwater	Fujian	NA	NA	28-30
mgp90129	Freshwater	Fujian	NA	NA	28-30
mgp90129	Freshwater	Fujian	NA	NA	28-30
mgp90129	Freshwater	Fujian	NA	NA	28-30
mgp90129	Freshwater	Fujian	NA	NA	28-30
mgp90129	Freshwater	Fujian	NA	NA	28-30
mgp90129	Freshwater	Fujian	NA	NA	28-30
mgp90129	Freshwater	Fujian	NA	NA	28-30
mgp90129	Freshwater	Fujian	NA	NA	28-30
mgp90129	Freshwater	Fujian	NA	NA	28-30
mgp90129	Freshwater	Fujian	NA	NA	28-30
mgp90129	Freshwater	Fujian	NA	NA	28-30
mgp90129	Freshwater	Fujian	NA	NA	28-30
mgp90129	Freshwater	Fujian	NA	NA	28-30
mgp90129	Freshwater	Fujian	NA	NA	28-30
mgp90129	Freshwater	Fujian	NA	NA	28-30
mgp90129	Freshwater	Fujian	NA	NA	28-30
mgp90129	Freshwater	Fujian	NA	NA	28-30
mgp90129	Freshwater	Fujian	NA	NA	28-30

mgp90129	Freshwater	Fujian	NA	NA	28-30
mgp90129	Freshwater	Fujian	NA	NA	28-30
mgp90129	Freshwater	Fujian	NA	NA	28-30
mgp90129	Freshwater	Fujian	NA	NA	28-30
mgp90129	Freshwater	Fujian	NA	NA	28-30
mgp90129	Freshwater	Fujian	NA	NA	28-30
mgp90129	Freshwater	Fujian	NA	NA	28-30
mgp90129	Freshwater	Fujian	NA	NA	28-30
mgp90129	Freshwater	Fujian	NA	NA	28-30
mgp90129	Freshwater	Fujian	NA	NA	28-30
mgp90129	Freshwater	Fujian	NA	NA	28-30
mgp90129	Freshwater	Fujian	NA	NA	28-30
mgp90129	Freshwater	Fujian	NA	NA	28-30
mgp90129	Freshwater	Fujian	NA	NA	28-30
mgp90129	Freshwater	Fujian	NA	NA	28-30
mgp90129	Freshwater	Fujian	NA	NA	28-30
mgp90129	Freshwater	Fujian	NA	NA	28-30
mgp90129	Freshwater	Fujian	NA	NA	28-30
mgp90129	Freshwater	Fujian	NA	NA	28-30
mgp90129	Freshwater	Fujian	NA	NA	28-30
mgp90461	Saline	North Adriatic	37-38	8.1	22-30
mgp90461	Saline	North Adriatic	37-38	8.1	22-30
mgp90461	Saline	North Adriatic	37-38	8.1	22-30
mgp91252	Alkaline	Lonar, India	0.63	10.3	28
mgp91252	Alkaline	Lonar, India	0.63	10.3	28
mgp91252	Alkaline	Lonar, India	0.63	10.3	28
mgp91252	Alkaline	Lonar, India	0.63	10.3	28
mgp91252	Alkaline	Lonar, India	0.63	10.3	28
mgp91252	Alkaline	Lonar, India	0.63	10.3	28
mgp91252	Alkaline	Lonar, India	0.63	10.3	28
mgp91252	Alkaline	Lonar, India	0.63	10.3	28
mgp95366	Freshwater	Punggol	NA	7.5	31
mgp20413	Saline	South Atlantic Ocean	34.3–35.8	8.05	20
mgp20413	Saline	South Atlantic Ocean	34.3–35.8	8.05	20
mgp20413	Saline	Indian Ocean	32-37	8.07 ± 0.02	30
mgp20413	Saline	South Pacific Ocean	37	8.1	1.6
mgp20413	Saline	South Atlantic Ocean	34.3–35.8	8.05	20
mgp20413	Saline	Red Sea	36-41	8.1±0.02	31
mgp20413	Saline	Mediterranean Sea	38	8.49 ± 0.01	27.2
mgp20413	Saline	Southern Ocean	34.6	7	6
mgp20413	Saline	South Atlantic Ocean	34.3–35.8	8.05	20

mgp20413	Saline	North Atlantic Ocean	34.8-35	7.9	20
mgp20413	Saline	Red Sea	36-41	8.1±0.02	31
mgp20413	Saline	North Atlantic Ocean	34.8-35	7.9	20
mgp20413	Saline	Red Sea	36-41	8.1±0.02	31
mgp20413	Saline	South Atlantic Ocean	34.3–35.8	8.05	20
mgp20413	Saline	South Atlantic Ocean	34.3–35.8	8.05	20
mgp20413	Saline	South Atlantic Ocean	34.3–35.8	8.05	20
mgp80088	Freshwater	Shanghai, China	<1.0	6.5	8
mgp80088	Freshwater	Shanghai, China	<1.0	6.5	8
mgp80088	Freshwater	Shanghai, China	<1.0	6.5	8
Current study	Saline	Sambhar lake, Rajasthan, India	~30	7.4	38

Supplementary Table S3. Phylogenetic affiliation of the rRNA features (16S and 18S rRNA gene) identified in the current raw sequence dataset. The rRNA features (16S and 18S rRNA gene) were clustered in genera using a similarity cutoff of >97%.

Domain	Phylum	Class	Order	Family	Genus	Abundance(%)
Bacteria	Tenericutes	Mollicutes	*Acholeplasmatales*	*Acholeplasmataceae*	*Acholeplasma*	0.079986
Bacteria	Firmicutes	Negativicutes	*Selenomonadales*	*Acidaminococcaceae*	*Acidaminococcus*	0.383157
Bacteria	Proteobacteria	Gammaproteobacteria	*Acidithiobacillales*	*Acidithiobacillaceae*	*Acidithiobacillus*	0.014191
Bacteria	Proteobacteria	Gammaproteobacteria	*Pseudomonadales*	*Moraxellaceae*	*Acinetobacter*	0.312202
Bacteria	Actinobacteria	Actinobacteria (class)	*Actinomycetales*	*Nocardioidaceae*	*Aeromicrobium*	0.00129
Bacteria	Proteobacteria	Gammaproteobacteria	*Aeromonadales*	*Aeromonadaceae*	*Aeromonas*	0.012901
Bacteria	Firmicutes	Bacilli	*Bacillales*	*Alicyclobacillaceae*	*Alicyclobacillus*	0.00129
Bacteria	Firmicutes	Clostridia	*Clostridiales*	*Clostridiaceae*	*Alkaliphilus*	0.014191
Bacteria	Proteobacteria	Gammaproteobacteria	*Chromatiales*	*Ectothiorhodospiraceae*	*Alkalispirillum*	0.28511
Bacteria	Firmicutes	Bacilli	*Bacillales*	*Bacillaceae*	*Anaerobacillus*	0.126429
Bacteria	Tenericutes	Mollicutes	*Anaeroplasmatales*	*Anaeroplasmataceae*	*Anaeroplasma*	0.14191
Bacteria	Actinobacteria	Actinobacteria (class)	*Coriobacteriales*	*Coriobacteriaceae*	*Atopobium*	0.00645
Bacteria	Proteobacteria	Alphaproteobacteria	*Rhizobiales*	*Aurantimonadaceae*	*Aurantimonas*	0.00258
Bacteria	Firmicutes	Bacilli	*Bacillales*	*Bacillaceae*	*Bacillus*	3.994117
Bacteria	Proteobacteria	Deltaproteobacteria	*Bdellovibrionales*	*Bacteriovoracaceae*	*Bacteriovorax*	0.00129
Bacteria	Bacteroidetes	Bacteroidia	*Bacteroidales*	*Bacteroidaceae*	*Bacteroides*	0.00516
Bacteria	Spirochaetes	Spirochaetes (class)	*Spirochaetales*	*Spirochaetaceae*	*Borrelia*	0.13933
Bacteria	Actinobacteria	Actinobacteria (class)	*Actinomycetales*	*Dermabacteraceae*	*Brachybacterium*	0.010321
Bacteria	Proteobacteria	Alphaproteobacteria	*Rhizobiales*	*Bradyrhizobiaceae*	*Bradyrhizobium*	0.024512
Bacteria	Firmicutes	Bacilli	*Bacillales*	*Paenibacillaceae*	*Brevibacillus*	0.059344
Bacteria	Bacteroidetes	Bacteroidia	*Bacteroidales*	*Porphyromonadaceae*	*Butyricimonas*	0.056764
Bacteria	Bacteroidetes	Flavobacteria	*Flavobacteriales*	*Flavobacteriaceae*	*Capnocytophaga*	0.030962
Eukaryota	Chlorophyta	Chlorophyceae	*Chlamydomonadales*	*Chlamydomonadaceae*	*Chlamydomonas*	4.18505
Bacteria	Proteobacteria	Betaproteobacteria	*Neisseriales*	*Neisseriaceae*	*Chromobacterium*	0.019351
Bacteria	Firmicutes	Clostridia	*Clostridiales*	*Clostridiaceae*	*Clostridium*	0.452821
Bacteria	Proteobacteria	Gammaproteobacteria	*Oceanospirillales*	*Halomonadaceae*	*Cobetia*	0.007741
Bacteria	Proteobacteria	Betaproteobacteria	*Burkholderiales*	*Comamonadaceae*	*Comamonas*	0.00645
Bacteria	Proteobacteria	Gammaproteobacteria	*unclassified (derived from Gammaproteobacteria)*	*unclassified (derived from Gammaproteobacteria)*	*Congregibacter*	0.015481
Bacteria	Proteobacteria	Gammaproteobacteria	*Enterobacteriales*	*Enterobacteriaceae*	*Cronobacter*	0.00129
Eukaryota	unclassified (derived from Eukaryota)	Bangiophyceae	*Cyanidiales*	*Cyanidiaceae*	*Cyanidium*	0.025802
Bacteria	Bacteroidetes	Cytophagia	*Cytophagales*	*Cytophagaceae*	*Cytophaga*	0.030962
Bacteria	Proteobacteria	Betaproteobacteria	*Burkholderiales*	*Comamonadaceae*	*Delftia*	0.00645
Bacteria	Firmicutes	Clostridia	*Clostridiales*	*Peptococcaceae*	*Desulfitobacterium*	0.00387
Bacteria	Proteobacteria	Deltaproteobacteria	*Desulfobacterales*	*Desulfobacteraceae*	*Desulfobacterium*	0.00129
Bacteria	Proteobacteria	Deltaproteobacteria	*unclassified (derived from Deltaproteobacteria)*	*unclassified (derived from Deltaproteobacteria)*	*Desulfocaldus*	0.14062
Bacteria	Proteobacteria	Deltaproteobacteria	*Desulfovibrionales*	*Desulfohalobiaceae*	*Desulfonatronovibrio*	0.069665

Bacteria	Firmicutes	Clostridia	*Clostridiales*	*Peptococcaceae*	*Desulfonispora*	0.766313
Bacteria	Firmicutes	Clostridia	*Clostridiales*	*Peptococcaceae*	*Desulfotomaculum*	0.00129
Bacteria	Proteobacteria	Deltaproteobacteria	*Desulfovibrionales*	*Desulfovibrionaceae*	*Desulfovibrio*	0.00258
Bacteria	Actinobacteria	Actinobacteria (class)	*Actinomycetales*	*Dietziaceae*	*Dietzia*	0.00129
Bacteria	Bacteroidetes	Flavobacteria	*Flavobacteriales*	*Flavobacteriaceae*	*Dokdonia*	0.154811
Bacteria	Proteobacteria	Gammaproteobacteria	*Chromatiales*	*Ectothiorhodospiraceae*	*Ectothiorhodospira*	0.00387
Bacteria	Actinobacteria	Actinobacteria (class)	*Coriobacteriales*	*Coriobacteriaceae*	*Eggerthella*	0.387027
Bacteria	Bacteroidetes	Flavobacteria	*Flavobacteriales*	*Flavobacteriaceae*	*Elizabethkingia*	0.945636
Bacteria	Firmicutes	Bacilli	*Lactobacillales*	*Enterococcaceae*	*Enterococcus*	0.157391
Bacteria	Firmicutes	Bacilli	*Bacillales*	*unclassified (derived from Bacillales)*	*Exiguobacterium*	0.00645
Bacteria	Thermotogae	Thermotogae (class)	*Thermotogales*	*Thermotogaceae*	*Fervidobacterium*	0.109658
Bacteria	Bacteroidetes	Cytophagia	*Cytophagales*	*Cytophagaceae*	*Flexibacter*	0.181903
Bacteria	Bacteroidetes	Cytophagia	*Cytophagales*	*Flammeovirgaceae*	*Flexithrix*	0.046443
Bacteria	Cyanobacteria	unclassified (derived from Cyanobacteria)	*Oscillatoriales*	*unclassified (derived from Oscillatoriales)*	*Geitlerinema*	0.029672
Bacteria	Firmicutes	Bacilli	*Bacillales*	*Bacillaceae*	*Geobacillus*	0.012901
Bacteria	Bacteroidetes	Flavobacteria	*Flavobacteriales*	*Flavobacteriaceae*	*Gramella*	0.00516
Eukaryota	unclassified (derived from Eukaryota)	Cryptophyta	*Pyrenomonadales*	*Geminigeraceae*	*Guillardia*	0.443791
Archaea	Euryarchaeota	Halobacteria	*Halobacteriales*	*Halobacteriaceae*	*Haladaptatus*	0.012901
Archaea	Euryarchaeota	Halobacteria	*Halobacteriales*	*Halobacteriaceae*	*Halalkalicoccus*	0.123849
Bacteria	Firmicutes	Clostridia	*Halanaerobiales*	*Halanaerobiaceae*	*Halanaerobium*	12.51903
Archaea	Euryarchaeota	Halobacteria	*Halobacteriales*	*Halobacteriaceae*	*Haloarcula*	0.14578
Archaea	Euryarchaeota	Halobacteria	*Halobacteriales*	*Halobacteriaceae*	*Halobacterium*	2.204763
Archaea	Euryarchaeota	Halobacteria	*Halobacteriales*	*Halobacteriaceae*	*Halobiforma*	0.646335
Bacteria	Proteobacteria	Gammaproteobacteria	*Chromatiales*	*Chromatiaceae*	*Halochromatium*	0.00129
Archaea	Euryarchaeota	Halobacteria	*Halobacteriales*	*Halobacteriaceae*	*Halococcus*	0.224476
Archaea	Euryarchaeota	Halobacteria	*Halobacteriales*	*Halobacteriaceae*	*Haloferax*	3.976056
Archaea	Euryarchaeota	Halobacteria	*Halobacteriales*	*Halobacteriaceae*	*Halogeometricum*	0.051604
Archaea	Euryarchaeota	Halobacteria	*Halobacteriales*	*Halobacteriaceae*	*Halogranum*	0.011611
Archaea	Euryarchaeota	Halobacteria	*Halobacteriales*	*Halobacteriaceae*	*Halomicrobium*	0.052894
Bacteria	Proteobacteria	Gammaproteobacteria	*Oceanospirillales*	*Halomonadaceae*	*Halomonas*	1.451351
Archaea	Euryarchaeota	Halobacteria	*Halobacteriales*	*Halobacteriaceae*	*Halopiger*	0.096757
Archaea	Euryarchaeota	Halobacteria	*Halobacteriales*	*Halobacteriaceae*	*Haloplanus*	0.897902
Archaea	Euryarchaeota	Halobacteria	*Halobacteriales*	*Halobacteriaceae*	*Haloquadratum*	0.443791
Archaea	Euryarchaeota	Halobacteria	*Halobacteriales*	*Halobacteriaceae*	*Halorhabdus*	0.232216
Bacteria	Proteobacteria	Gammaproteobacteria	*Chromatiales*	*Ectothiorhodospiraceae*	*Halorhodospira*	0.415409
Archaea	Euryarchaeota	Halobacteria	*Halobacteriales*	*Halobacteriaceae*	*Halorubrum*	20.70465
Archaea	Euryarchaeota	Halobacteria	*Halobacteriales*	*Halobacteriaceae*	*Halosimplex*	0.445081
Archaea	Euryarchaeota	Halobacteria	*Halobacteriales*	*Halobacteriaceae*	*Halostagnicola*	0.079986
Archaea	Euryarchaeota	Halobacteria	*Halobacteriales*	*Halobacteriaceae*	*Haloterrigena*	1.691307
Bacteria	Firmicutes	Clostridia	*Halanaerobiales*	*Halanaerobiaceae*	*Halothermothrix*	0.586991
Bacteria	Proteobacteria	Gammaproteobacteria	*Chromatiales*	*Halothiobacillaceae*	*Halothiobacillus*	0.116108
Archaea	Euryarchaeota	Halobacteria	*Halobacteriales*	*Halobacteriaceae*	*Halovivax*	0.096757

Bacteria	Bacteroidetes	Cytophagia	*Cytophagales*	*Cytophagaceae*	*Hymenobacter*	0.037413
Bacteria	Bacteroidetes	Flavobacteria	*Flavobacteriales*	*Flavobacteriaceae*	*Kordia*	0.00258
Bacteria	Bacteroidetes	Flavobacteria	*Flavobacteriales*	*Flavobacteriaceae*	*Leeuwenhoekiella*	0.099337
Bacteria	Nitrospirae	Nitrospira (class)	*Nitrospirales*	*Nitrospiraceae*	*Leptospirillum*	0.065795
Bacteria	Fusobacteria	Fusobacteria (class)	*Fusobacteriales*	*Fusobacteriaceae*	*Leptotrichia*	0.00129
Bacteria	Proteobacteria	Gammaproteobacteria	*Chromatiales*	*Chromatiaceae*	*Marichromatium*	0.00645
Bacteria	Proteobacteria	Gammaproteobacteria	*Alteromonadales*	*Alteromonadaceae*	*Marinobacter*	0.330263
Bacteria	Proteobacteria	Gammaproteobacteria	*Oceanospirillales*	*Oceanospirillaceae*	*Marinospirillum*	0.019351
Bacteria	Proteobacteria	Alphaproteobacteria	*Rhizobiales*	*Aurantimonadaceae*	*Martelella*	0.185773
Bacteria	Firmicutes	Bacilli	*Lactobacillales*	*Enterococcaceae*	*Melissococcus*	0.109658
Archaea	Euryarchaeota	Methanobacteria	*Methanobacteriales*	*Methanobacteriaceae*	*Methanobacterium*	0.054184
Bacteria	Proteobacteria	Gammaproteobacteria	*Methylococcales*	*Methylococcaceae*	*Methylohalobius*	0.021932
Bacteria	Cyanobacteria	unclassified (derived from Cyanobacteria)	*Chroococcales*	*unclassified (derived from Chroococcales)*	*Microcystis*	0.00387
Bacteria	Firmicutes	Clostridia	*Thermoanaerobacterales*	*Thermoanaerobacteraceae*	*Moorella*	0.00129
Bacteria	Actinobacteria	Actinobacteria (class)	*Actinomycetales*	*Mycobacteriaceae*	*Mycobacterium*	0.162551
Bacteria	Bacteroidetes	Flavobacteria	*Flavobacteriales*	*Flavobacteriaceae*	*Myroides*	0.036123
Archaea	Euryarchaeota	Halobacteria	*Halobacteriales*	*Halobacteriaceae*	*Natrialba*	0.841138
Archaea	Euryarchaeota	Halobacteria	*Halobacteriales*	*Halobacteriaceae*	*Natrinema*	0.112238
Archaea	Euryarchaeota	Halobacteria	*Halobacteriales*	*Halobacteriaceae*	*Natronobacterium*	0.619243
Archaea	Euryarchaeota	Halobacteria	*Halobacteriales*	*Halobacteriaceae*	*Natronococcus*	0.034832
Archaea	Euryarchaeota	Halobacteria	*Halobacteriales*	*Halobacteriaceae*	*Natronolimnobius*	1.314601
Archaea	Euryarchaeota	Halobacteria	*Halobacteriales*	*Halobacteriaceae*	*Natronorubrum*	0.639884
Bacteria	Proteobacteria	Alphaproteobacteria	*Rhodobacterales*	*Rhodobacteraceae*	*Nereida*	0.215445
Bacteria	Proteobacteria	Gammaproteobacteria	*Chromatiales*	*Chromatiaceae*	*Nitrosococcus*	0.025802
Bacteria	Actinobacteria	Actinobacteria (class)	*Actinomycetales*	*Nocardioidaceae*	*Nocardioides*	0.00129
Bacteria	Actinobacteria	Actinobacteria (class)	*Actinomycetales*	*Nocardiopsaceae*	*Nocardiopsis*	0.526357
Bacteria	Bacteroidetes	Flavobacteria	*Flavobacteriales*	*Flavobacteriaceae*	*Ornithobacterium*	0.00258
Bacteria	Cyanobacteria	unclassified (derived from Cyanobacteria)	*Oscillatoriales*	*unclassified (derived from Oscillatoriales)*	*Oscillatoria*	0.054184
Eukaryota	Chlorophyta	Ulvophyceae	*Bryopsidales*	*Ostreobiaceae*	*Ostreobium*	0.319942
Bacteria	Firmicutes	Bacilli	*Bacillales*	*Paenibacillaceae*	*Paenibacillus*	0.094177
Bacteria	Bacteroidetes	Sphingobacteria	*Sphingobacteriales*	*Sphingobacteriaceae*	*Pedobacter*	0.030962
Eukaryota	Chlorophyta	Chlorophyceae	*Chlamydomonadales*	*Chlamydomonadaceae*	*Polytoma*	1.708079
Bacteria	Bacteroidetes	Bacteroidia	*Bacteroidales*	*Porphyromonadaceae*	*Porphyromonas*	0.063214
Bacteria	Cyanobacteria	unclassified (derived from Cyanobacteria)	*Prochlorales*	*Prochlorococcaceae*	*Prochlorococcus*	0.189643
Bacteria	Actinobacteria	Actinobacteria (class)	*Actinomycetales*	*Propionibacteriaceae*	*Propionibacterium*	0.007741
Bacteria	Proteobacteria	Gammaproteobacteria	*Pseudomonadales*	*Pseudomonadaceae*	*Pseudomonas*	0.00258
Bacteria	Actinobacteria	Actinobacteria (class)	*Actinomycetales*	*Pseudonocardiaceae*	*Pseudonocardia*	0.00129
Bacteria	Bacteroidetes	Flavobacteria	*Flavobacteriales*	*Flavobacteriaceae*	*Psychroflexus*	0.010321
Eukaryota	Bacteroidetes unclassified (derived from Eukaryota)	Florideophyceae	*Gelidiales*	*Gelidiaceae*	*Pterocladia*	0.14707
Bacteria	Firmicutes	Bacilli	*Bacillales*	*unclassified (derived from Bacillales)*	*Pullulanibacillus*	0.058054
Bacteria	Proteobacteria	Alphaproteobacteria	*Rhodobacterales*	*Rhodobacteraceae*	*Rhodobaca*	0.086436
Bacteria	Proteobacteria	Alphaproteobacteria	*Rhodobacterales*	*Rhodobacteraceae*	*Rhodobacter*	0.1432

Bacteria	Proteobacteria	Alphaproteobacteria	*Rhizobiales*	*Bradyrhizobiaceae*	*Rhodopseudomonas*	0.00129
Bacteria	Proteobacteria	Alphaproteobacteria	*Rhodobacterales*	*Rhodobacteraceae*	*Rhodovulum*	0.00387
Bacteria	Bacteroidetes	Flavobacteria	*Flavobacteriales*	*Flavobacteriaceae*	*Riemerella*	0.00129
Bacteria	Proteobacteria	Alphaproteobacteria	*Rhodobacterales*	*Rhodobacteraceae*	*Roseobacter*	0.00129
Bacteria	Firmicutes	Clostridia	*Clostridiales*	*Ruminococcaceae*	*Ruminococcus*	0.00129
Bacteria	Bacteroidetes	Sphingobacteria	*Sphingobacteriales*	*Rhodothermaceae*	*Salinibacter*	1.651315
Bacteria	Firmicutes	Bacilli	*Bacillales*	*Thermoactinomycetaceae*	*Seinonella*	0.00645
Bacteria	Proteobacteria	Gammaproteobacteria	*Alteromonadales*	*Shewanellaceae*	*Shewanella*	0.010321
Bacteria	Firmicutes	Clostridia	*Clostridiales*	*Clostridiales Family XI. Incertae Sedis*	*Soehngenia*	0.104497
Bacteria	Spirochaetes	Spirochaetes (class)	*Spirochaetales*	*Spirochaetaceae*	*Spirochaeta*	0.113528
Bacteria	Actinobacteria	Actinobacteria (class)	*Actinomycetales*	*Streptomycetaceae*	*Streptomyces*	0.165131
Bacteria	Firmicutes	Clostridia	*Clostridiales*	*Clostridiales Family XVIII. Incertae Sedis*	*Symbiobacterium*	0.018061
Bacteria	Cyanobacteria	unclassified (derived from Cyanobacteria)	*Chroococcales*	*unclassified (derived from Chroococcales)*	*Synechococcus*	0.239957
Bacteria	Proteobacteria	Deltaproteobacteria	*Syntrophobacterales*	*Syntrophobacteraceae*	*Syntrophobacter*	0.011611
Bacteria	Firmicutes	Clostridia	*Clostridiales*	*Syntrophomonadaceae*	*Syntrophomonas*	0.012901
Bacteria	Bacteroidetes	Flavobacteria	*Flavobacteriales*	*Flavobacteriaceae*	*Tenacibaculum*	0.030962
Bacteria	Bacteroidetes	Sphingobacteria	*Sphingobacteriales*	*unclassified (derived from Sphingobacteriales)*	*Terrimonas*	0.00129
Bacteria	Firmicutes	Clostridia	*Thermoanaerobacterales*	*Thermoanaerobacteraceae*	*Thermacetogenium*	0.010321
Bacteria	Actinobacteria	Actinobacteria (class)	*Actinomycetales*	*Pseudonocardiaceae*	*Thermobispora*	1.324922
Bacteria	Firmicutes	Clostridia	*Thermoanaerobacterales*	*Thermodesulfobiaceae*	*Thermodesulfobium*	0.00516
Bacteria	Actinobacteria	Actinobacteria (class)	*Thermoleophilales*	*Thermoleophilaceae*	*Thermoleophilum*	0.00387
Bacteria	Deinococcus-Thermus	Deinococci	*Thermales*	*Thermaceae*	*Thermus*	0.018061
Bacteria	Proteobacteria	Gammaproteobacteria	*Chromatiales*	*Halothiobacillaceae*	*Thioalkalibacter*	0.221895
Bacteria	Proteobacteria	Gammaproteobacteria	*Chromatiales*	*Ectothiorhodospiraceae*	*Thioalkalivibrio*	0.023222
Bacteria	Firmicutes	Clostridia	*Clostridiales*	*Clostridiales Family XI. Incertae Sedis*	*Tissierella*	0.025802
Bacteria	Proteobacteria	Alphaproteobacteria	*Rhodospirillales*	*Rhodospirillaceae*	*Tistrella*	0.00258
Bacteria	Spirochaetes	Spirochaetes (class)	*Spirochaetales*	*Spirochaetaceae*	*Treponema*	0.194804
Bacteria	Actinobacteria	Actinobacteria (class)	*Actinomycetales*	*unclassified (derived from Actinomycetales)*	*Tropheryma*	0.00645
Bacteria	Bacteroidetes	Flavobacteria	*Flavobacteriales*	*Flavobacteriaceae*	*Wautersiella*	0.033542
Bacteria	Proteobacteria	Alphaproteobacteria	*unclassified (derived from Alphaproteobacteria)*	*unclassified (derived from Alphaproteobacteria)*	*unclassified (derived from Alphaproteobacteria)*	1.804835
Archaea	unclassified (derived from Archaea)	unclassified (derived from Archaea)	*unclassified (derived from Archaea)*	*unclassified (derived from Archaea)*	*unclassified (derived from Archaea)*	0.839848
Bacteria	unclassified (derived from Bacteria)	unclassified (derived from Bacteria)	*unclassified (derived from Bacteria)*	*unclassified (derived from Bacteria)*	*unclassified (derived from Bacteria)*	12.70351
Bacteria	Bacteroidetes	unclassified (derived from Bacteroidetes)	*unclassified (derived from Bacteroidetes)*	*unclassified (derived from Bacteroidetes)*	*unclassified (derived from Bacteroidetes)*	0.049023
Bacteria	Proteobacteria	Betaproteobacteria	*unclassified (derived from Betaproteobacteria)*	*unclassified (derived from Betaproteobacteria)*	*unclassified (derived from Betaproteobacteria)*	0.029672
Bacteria	Cyanobacteria	unclassified (derived from Cyanobacteria)	*unclassified (derived from Cyanobacteria)*	*unclassified (derived from Cyanobacteria)*	*unclassified (derived from Cyanobacteria)*	0.00129
Bacteria	Proteobacteria	Deltaproteobacteria	*unclassified (derived from*	*unclassified (derived from Deltaproteobacteria)*	*unclassified (derived from*	0.065795

			Deltaproteobacteria)		*Deltaproteobacteria)*	
Bacteria	Proteobacteria	Epsilonproteobacteria	*unclassified (derived from Epsilonproteobacteria)*	*unclassified (derived from Epsilonproteobacteria)*	*unclassified (derived from Epsilonproteobacteria)*	0.012901
Bacteria	Firmicutes	unclassified (derived from Firmicutes)	*unclassified (derived from Firmicutes)*	*unclassified (derived from Firmicutes)*	*unclassified (derived from Firmicutes)*	0.740511
Bacteria	Bacteroidetes	Flavobacteria	*Flavobacteriales*	*Flavobacteriaceae*	*unclassified (derived from Flavobacteriaceae)*	0.007741
Bacteria	Proteobacteria	Gammaproteobacteria	*unclassified (derived from Gammaproteobacteria)*	*unclassified (derived from Gammaproteobacteria)*	*unclassified (derived from Gammaproteobacteria)*	6.152437
Archaea	Euryarchaeota	Halobacteria	*Halobacteriales*	*unclassified (derived from Halobacteriales)*	*unclassified (derived from Halobacteriales)*	1.047553
Bacteria	Cyanobacteria	unclassified (derived from Cyanobacteria)	*Oscillatoriales*	*unclassified (derived from Oscillatoriales)*	*unclassified (derived from Oscillatoriales)*	0.007741
Bacteria	Proteobacteria	unclassified (derived from Proteobacteria)	*unclassified (derived from Proteobacteria)*	*unclassified (derived from Proteobacteria)*	*unclassified (derived from Proteobacteria)*	1.835797
Bacteria	Proteobacteria	Alphaproteobacteria	*Rhodobacterales*	*Rhodobacteraceae*	*unclassified (derived from Rhodobacteraceae)*	0.13804
Bacteria	Proteobacteria	Gammaproteobacteria	*Salinisphaerales*	*Salinisphaeraceae*	*unclassified (derived from Salinisphaeraceae)*	0.708259
Bacteria	Synergistetes	unclassified (derived from Synergistetes)	*unclassified (derived from Synergistetes)*	*unclassified (derived from Synergistetes)*	*unclassified (derived from Synergistetes)*	0.011611
unclassified sequences	unclassified (derived from unclassified sequences)	unclassified (derived from unclassified sequences)	*unclassified (derived from unclassified sequences)*	*unclassified (derived from unclassified sequences)*	*unclassified (derived from unclassified sequences)*	0.054184

Supplementary Table S4. Phylogenetic affiliation of the protein features identified in the current datasets (raw and assembled). Protein features were clustered based on their similarity (>97%) for the calculation of relative abundance.

Domain	Phylum	Genus	Realtive Abundance (%) in assembled dataset	Abundance (%) in raw reads dataset
Archaea	Crenarchaeota	*Acidilobus*	0.001465014	0.001653151
Archaea	Euryarchaeota	*Aciduliprofundum*	0.011720109	0.011889509
Archaea	Crenarchaeota	*Aeropyrum*	0.012452616	0.013428856
Archaea	Euryarchaeota	*Archaeoglobus*	0.083505778	0.058944412
Archaea	Crenarchaeota	*Caldivirga*	0.003662534	0.003234426
Archaea	Korarchaeota	*Candidatus Korarchaeum*	0.007325068	0.00667849
Archaea	Thaumarchaeota	*Cenarchaeum*	0.006226308	0.001437522
Archaea	Crenarchaeota	*Desulfurococcus*	0.003662534	0.003318281
Archaea	Euryarchaeota	*Ferroglobus*	0.024905232	0.014608822
Archaea	Euryarchaeota	*Ferroplasma*	0.003662534	0.00277322
Archaea	Euryarchaeota	*Halalkalicoccus*	1.631292691	1.316285435
Archaea	Euryarchaeota	*Haloarcula*	7.255113813	6.407001789
Archaea	Euryarchaeota	*Halobacterium*	3.829179409	4.205951211
Archaea	Euryarchaeota	*Haloferax*	2.517992199	2.626461399
Archaea	Euryarchaeota	*Halogeometricum*	5.353892358	5.440914801
Archaea	Euryarchaeota	*Halomicrobium*	2.705147692	2.312135128
Archaea	Euryarchaeota	*Haloquadratum*	4.358415588	4.926563265
Archaea	Euryarchaeota	*Halorhabdus*	2.274433681	1.819651903
Archaea	Euryarchaeota	*Halorubrum*	7.560569158	11.26215013
Archaea	Euryarchaeota	*Haloterrigena*	4.438258831	3.051099552
Archaea	Crenarchaeota	*Hyperthermus*	0.007325068	0.00649281
Archaea	Crenarchaeota	*Ignicoccus*	0.005860055	0.005366751
Archaea	Crenarchaeota	*Ignisphaera*	0.004028788	0.00203649
Archaea	Crenarchaeota	*Metallosphaera*	0.005127548	0.004150846
Archaea	Euryarchaeota	*Methanobrevibacter*	0.03003278	0.024204285
Archaea	Euryarchaeota	*Methanocaldococcus*	0.113172304	0.060974912
Archaea	Euryarchaeota	*Methanocella*	0.010621349	0.00908634
Archaea	Euryarchaeota	*Methanococcoides*	0.050542971	0.035117476
Archaea	Euryarchaeota	*Methanococcus*	0.088633325	0.063820009
Archaea	Euryarchaeota	*Methanocorpusculum*	0.019045177	0.015477325
Archaea	Euryarchaeota	*Methanoculleus*	0.046514183	0.033733861
Archaea	Euryarchaeota	*Methanohalobium*	0.031864047	0.018076845
Archaea	Euryarchaeota	*Methanohalophilus*	0.025271485	0.017076569
Archaea	Euryarchaeota	*Methanoplanus*	0.012818869	0.009212123
Archaea	Euryarchaeota	*Methanopyrus*	0.043950409	0.028259296
Archaea	Euryarchaeota	*Methanoregula*	0.025271485	0.021604765
Archaea	Euryarchaeota	*Methanosaeta*	0.028934019	0.028834305
Archaea	Euryarchaeota	*Methanosarcina*	0.145768857	0.111384034

Archaea	Euryarchaeota	*Methanosphaera*	0.016847657	0.012362693
Archaea	Euryarchaeota	*Methanosphaerula*	0.021242698	0.015052058
Archaea	Euryarchaeota	*Methanospirillum*	0.044682916	0.02855279
Archaea	Euryarchaeota	*Methanothermobacter*	0.085337045	0.047707778
Archaea	Euryarchaeota	*Methanothermococcus*	0.005493801	0.003749538
Archaea	Euryarchaeota	*Methanothermus*	0.014283883	0.00890066
Archaea	Nanoarchaeota	*Nanoarchaeum*	0.013551376	0.006013636
Archaea	Euryarchaeota	*Natrialba*	3.955170583	3.261127577
Archaea	Euryarchaeota	*Natrinema*	0	9.58348E-05
Archaea	Euryarchaeota	*Natronobacterium*	0.001465014	0.000491154
Archaea	Euryarchaeota	*Natronomonas*	9.757357115	7.215374591
Archaea	Thaumarchaeota	*Nitrosopumilus*	0.008057575	0.006217285
Archaea	Euryarchaeota	*Picrophilus*	0.007325068	0.005696183
Archaea	Crenarchaeota	*Pyrobaculum*	0.025637739	0.014800492
Archaea	Euryarchaeota	*Pyrococcus*	0.110974783	0.075380085
Archaea	Crenarchaeota	*Staphylothermus*	0.009522589	0.008703001
Archaea	Crenarchaeota	*Sulfolobus*	0.03003278	0.019741975
Archaea	Euryarchaeota	*Thermococcus*	0.098522167	0.071792269
Archaea	Crenarchaeota	*Thermofilum*	0.014650136	0.007852467
Archaea	Euryarchaeota	*Thermoplasma*	0.010255096	0.011092882
Archaea	Crenarchaeota	*Thermoproteus*	0.001831267	0.001413564
Archaea	Crenarchaeota	*Thermosphaera*	0.001831267	0.002090397
Archaea	Crenarchaeota	*Vulcanisaeta*	0.004028788	0.002102377
Archaea	Euryarchaeota	*Unclassified Euryarchaeota*	0.053106745	0.036560989
Bacteria	Firmicutes	*Abiotrophia*	0.005127548	0.00612145
Bacteria	Cyanobacteria	*Acaryochloris*	0.040287875	0.032290349
Bacteria	Firmicutes	*Acetivibrio*	0.0322303	0.020263077
Bacteria	Proteobacteria	*Acetobacter*	0.005860055	0.006690469
Bacteria	Firmicutes	*Acetohalobium*	1.304960902	0.900835446
Bacteria	Tenericutes	*Acholeplasma*	0.061896826	0.013632505
Bacteria	Proteobacteria	*Achromobacter*	0.03003278	0.047126779
Bacteria	Firmicutes	*Acidaminococcus*	0.018678924	0.025324354
Bacteria	Actinobacteria	*Acidimicrobium*	0.007691322	0.007900384
Bacteria	Proteobacteria	*Acidiphilium*	0.030399033	0.04283817
Bacteria	Proteobacteria	*Acidithiobacillus*	0.032962807	0.074218088
Bacteria	Acidobacteria	*Acidobacterium*	0.034794074	0.038088356
Bacteria	Actinobacteria	*Acidothermus*	0.017946417	0.018412267
Bacteria	Proteobacteria	*Acidovorax*	0.07727947	0.106161036
Bacteria	Proteobacteria	*Acinetobacter*	0.064826854	0.136187287
Bacteria	Proteobacteria	*Actinobacillus*	0.024905232	0.031266114
Bacteria	Actinobacteria	*Actinomyces*	0.012818869	0.015890613
Bacteria	Actinobacteria	*Actinosynnema*	0.011353856	0.013806206
Bacteria	Firmicutes	*Aerococcus*	0.005860055	0.006738387
Bacteria	Actinobacteria	*Aeromicrobium*	0.003662534	0.003755527

Bacteria	Proteobacteria	*Aeromonas*	0.028934019	0.075044664
Bacteria	Proteobacteria	*Afipia*	0.006958815	0.004683927
Bacteria	Proteobacteria	*Aggregatibacter*	0.004028788	0.007648818
Bacteria	Proteobacteria	*Agrobacterium*	0.062995587	0.056081346
Bacteria	Proteobacteria	*Ahrensia*	0.006226308	0.014165586
Bacteria	Verrucomicrobia	*Akkermansia*	0.008057575	0.009954843
Bacteria	Proteobacteria	*Albidiferax*	0.039921622	0.048504405
Bacteria	Proteobacteria	*Alcanivorax*	0.085337045	0.151802375
Bacteria	Bacteroidetes	*Algoriphagus*	0.086435805	0.039669631
Bacteria	Proteobacteria	*Alicycliphilus*	0.007325068	0.01020641
Bacteria	Firmicutes	*Alicyclobacillus*	0.038822862	0.036045876
Bacteria	Proteobacteria	*Aliivibrio*	0.025271485	0.038884983
Bacteria	Bacteroidetes	*Alistipes*	0.01501639	0.008056116
Bacteria	Proteobacteria	*Alkalilimnicola*	0.534363726	1.47214881
Bacteria	Firmicutes	*Alkaliphilus*	0.499569652	0.389670419
Bacteria	Proteobacteria	*Allochromatium*	0.042851649	0.148094765
Bacteria	Proteobacteria	*Alteromonas*	0.032962807	0.044485331
Bacteria	Synergistetes	*Aminobacterium*	0.020510191	0.023725111
Bacteria	Synergistetes	*Aminomonas*	0.009888842	0.005965718
Bacteria	Firmicutes	*Ammonifex*	0.036259088	0.029816612
Bacteria	Actinobacteria	*Amycolatopsis*	0.012818869	0.013339011
Bacteria	Cyanobacteria	*Anabaena*	0.073250682	0.0519305
Bacteria	Synergistetes	*Anaerobaculum*	0.048711704	0.054140691
Bacteria	Firmicutes	*Anaerococcus*	0.019777684	0.034925807
Bacteria	Firmicutes	*Anaerofustis*	0.005860055	0.009325927
Bacteria	Proteobacteria	*Anaeromyxobacter*	0.122694893	0.107275116
Bacteria	Firmicutes	*Anaerostipes*	0.009522589	0.013920009
Bacteria	Firmicutes	*Anaerotruncus*	0.008057575	0.009727235
Bacteria	Proteobacteria	*Anaplasma*	0.001831267	0.002671396
Bacteria	Firmicutes	*Anoxybacillus*	0.042485396	0.030679126
Bacteria	Aquificae	*Aquifex*	0.034061567	0.032955203
Bacteria	Actinobacteria	*Arcanobacterium*	0.000732507	0.002156284
Bacteria	Proteobacteria	*Arcobacter*	0.017580164	0.017705485
Bacteria	Proteobacteria	*Aromatoleum*	0.059333053	0.08586202
Bacteria	Actinobacteria	*Arthrobacter*	0.043950409	0.042856139
Bacteria	Cyanobacteria	*Arthrospira*	0.04834545	0.039921197
Bacteria	Proteobacteria	*Asticcacaulis*	0.006226308	0.008289713
Bacteria	Actinobacteria	*Atopobium*	0.004395041	0.009499628
Bacteria	Proteobacteria	*Aurantimonas*	0.027469006	0.036207598
Bacteria	Proteobacteria	*Azoarcus*	0.042851649	0.069114883
Bacteria	Proteobacteria	*Azorhizobium*	0.034427821	0.029708798
Bacteria	Proteobacteria	*Azospirillum*	0.028934019	0.039963125
Bacteria	Proteobacteria	*Azotobacter*	0.052740491	0.055158936
Bacteria	Firmicutes	*Bacillus*	1.121467944	0.897762742

Bacteria	Bacteroidetes	*Bacteroides*	0.279451352	0.178372582
Bacteria	Proteobacteria	*Bartonella*	0.008057575	0.014351266
Bacteria	Proteobacteria	*Basfia*	0.008790082	0.011524139
Bacteria	Proteobacteria	*Bdellovibrio*	0.020876444	0.022880566
Bacteria	Proteobacteria	*Beggiatoa*	0.027102752	0.036920369
Bacteria	Proteobacteria	*Beijerinckia*	0.011353856	0.014572884
Bacteria	Proteobacteria	*Bermanella*	0.009888842	0.030068179
Bacteria	Actinobacteria	*Beutenbergia*	0.01391763	0.008990505
Bacteria	Proteobacteria	*Bibersteinia*	0	5.98968E-06
Bacteria	Actinobacteria	*Bifidobacterium*	0.019411431	0.021556848
Bacteria	Planctomycetes	*Blastopirellula*	0.025637739	0.021682631
Bacteria	Bacteroidetes	*Blattabacterium*	0.002930027	0.002845097
Bacteria	Firmicutes	*Blautia*	0.01391763	0.01557316
Bacteria	Proteobacteria	*Bordetella*	0.112439797	0.125597538
Bacteria	Spirochaetes	*Borrelia*	0.054205505	0.020820117
Bacteria	Actinobacteria	*Brachybacterium*	0.009888842	0.010242348
Bacteria	Spirochaetes	*Brachyspira*	0.031497793	0.022778742
Bacteria	Proteobacteria	*Bradyrhizobium*	0.119032358	0.107622517
Bacteria	Firmicutes	*Brevibacillus*	0.043217902	0.045833009
Bacteria	Actinobacteria	*Brevibacterium*	0.014650136	0.017106518
Bacteria	Proteobacteria	*Brevundimonas*	0.011720109	0.0142734
Bacteria	Proteobacteria	*Brucella*	0.027835259	0.048749981
Bacteria	Proteobacteria	*Buchnera*	0.00219752	0.007325375
Bacteria	Firmicutes	*Bulleidia*	0.002930027	0.00222816
Bacteria	Proteobacteria	*Burkholderia*	0.294833996	0.364771331
Bacteria	Firmicutes	*Butyrivibrio*	0.014283883	0.013081455
Bacteria	Firmicutes	*Caldanaerobacter*	0.23623345	0.189369629
Bacteria	Firmicutes	*Caldicellulosiruptor*	0.167377809	0.140469906
Bacteria	Deferribacteres	*Calditerrivibrio*	0.018678924	0.01187154
Bacteria	Proteobacteria	*Caminibacter*	0.002930027	0.005648265
Bacteria	Proteobacteria	*Campylobacter*	0.038822862	0.04450929
Bacteria	Proteobacteria	*Candidatus Accumulibacter*	0.031497793	0.043568911
Bacteria	Bacteroidetes	*Candidatus Amoebophilus*	0.01501639	0.009781143
Bacteria	Bacteroidetes	*Candidatus Azobacteroides*	0.005493801	0.002755251
Bacteria	Proteobacteria	*Candidatus Blochmannia*	0.000366253	0.003204477
Bacteria	Proteobacteria	*Candidatus Carsonella*	0	8.98452E-05
Bacteria	unclassified (derived from Bacteria)	*Candidatus Cloacamonas*	0.016847657	0.009966823
Bacteria	Firmicutes	*Candidatus Desulforudis*	0.06116432	0.054733669
Bacteria	Proteobacteria	*Candidatus Hamiltonella*	0.000732507	0.004474289
Bacteria	Proteobacteria	*Candidatus Hodgkinia*	0	5.98968E-06
Bacteria	Acidobacteria	*Candidatus Koribacter*	0.053472998	0.039430044
Bacteria	Proteobacteria	*Candidatus Liberibacter*	0.000732507	0.001102101
Bacteria	Proteobacteria	*Candidatus Pelagibacter*	0.013551376	0.024276161
Bacteria	Tenericutes	*Candidatus Phytoplasma*	0.015748897	0.005246957

Bacteria	Chlamydiae	*Candidatus Protochlamydia*	0.007325068	0.007511055
Bacteria	Proteobacteria	*Candidatus Puniceispirillum*	0.011720109	0.018412267
Bacteria	Proteobacteria	*Candidatus Regiella*	0.002563774	0.0037136
Bacteria	Proteobacteria	*Candidatus Riesia*	0.000366253	0.000700792
Bacteria	Acidobacteria	*Candidatus Solibacter*	0.083139524	0.07732673
Bacteria	Bacteroidetes	*Candidatus Sulcia*	0.002930027	0.00129976
Bacteria	Proteobacteria	*Candidatus Zinderia*	0	2.99484E-05
Bacteria	Bacteroidetes	*Capnocytophaga*	0.031864047	0.019059152
Bacteria	Firmicutes	*Carboxydothermus*	0.164447781	0.14095507
Bacteria	Proteobacteria	*Cardiobacterium*	0.006592561	0.018831545
Bacteria	Firmicutes	*Carnobacterium*	0.011353856	0.008032157
Bacteria	Firmicutes	*Catenibacterium*	0.009156335	0.005145133
Bacteria	Actinobacteria	*Catenulispora*	0.015382643	0.015267687
Bacteria	Firmicutes	*Catonella*	0.004028788	0.003719589
Bacteria	Proteobacteria	*Caulobacter*	0.041752889	0.049486712
Bacteria	Actinobacteria	*Cellulomonas*	0.005860055	0.010739491
Bacteria	Firmicutes	*Cellulosilyticum*	0.016481403	0.019771924
Bacteria	Proteobacteria	*Cellvibrio*	0.035160327	0.04803122
Bacteria	Proteobacteria	*Chelativorans*	0.057135532	0.05600947
Bacteria	Bacteroidetes	*Chitinophaga*	0.075448203	0.042209254
Bacteria	Chlamydiae	*Chlamydia*	0.00219752	0.004504237
Bacteria	Chlamydiae	*Chlamydophila*	0.004395041	0.007487096
Bacteria	Chlorobi	*Chlorobaculum*	0.068855641	0.061244448
Bacteria	Chlorobi	*Chlorobium*	0.199608109	0.151269293
Bacteria	Chloroflexi	*Chloroflexus*	0.11170729	0.081567422
Bacteria	Chlorobi	*Chloroherpeton*	0.088633325	0.0556441
Bacteria	Proteobacteria	*Chromobacterium*	0.03113154	0.054871431
Bacteria	Proteobacteria	*Chromohalobacter*	0.926987383	0.524042833
Bacteria	Bacteroidetes	*Chryseobacterium*	0.018678924	0.011170748
Bacteria	Verrucomicrobia	*Chthoniobacter*	0.020510191	0.017220321
Bacteria	Proteobacteria	*Citreicella*	0.038822862	0.034572416
Bacteria	Proteobacteria	*Citrobacter*	0.013551376	0.020275057
Bacteria	Proteobacteria	*Citromicrobium*	0.004028788	0.010469955
Bacteria	Actinobacteria	*Clavibacter*	0.012086363	0.01353667
Bacteria	Firmicutes	*Clostridium*	1.367590236	1.192083492
Bacteria	Proteobacteria	*Collimonas*	0	0.000137763
Bacteria	Actinobacteria	*Collinsella*	0.004761294	0.008451434
Bacteria	Proteobacteria	*Colwellia*	0.027835259	0.04265249
Bacteria	Proteobacteria	*Comamonas*	0.024172725	0.018454195
Bacteria	Actinobacteria	*Conexibacter*	0.042119142	0.036860472
Bacteria	Proteobacteria	*Congregibacter*	0.03113154	0.054961276
Bacteria	Firmicutes	*Coprobacillus*	0.006226308	0.005288885
Bacteria	Firmicutes	*Coprococcus*	0.008057575	0.01150018
Bacteria	Firmicutes	*Coprothermobacter*	0.020143938	0.019580254

Bacteria	Verrucomicrobia	*Coraliomargarita*	0.016847657	0.017951062
Bacteria	Actinobacteria	*Corynebacterium*	0.06336184	0.080639022
Bacteria	Proteobacteria	*Coxiella*	0.024905232	0.047180686
Bacteria	Bacteroidetes	*Croceibacter*	0.057868039	0.032511967
Bacteria	Cyanobacteria	*Crocosphaera*	0.024538979	0.018466174
Bacteria	Proteobacteria	*Cronobacter*	0.014283883	0.020844076
Bacteria	Actinobacteria	*Cryptobacterium*	0.004761294	0.004875597
Bacteria	Proteobacteria	*Cupriavidus*	0.10950977	0.116433332
Bacteria	Cyanobacteria	*Cyanobium*	0.008057575	0.00594176
Bacteria	Cyanobacteria	*Cyanothece*	0.242093504	0.171789927
Bacteria	Cyanobacteria	*Cylindrospermopsis*	0.013185123	0.011512159
Bacteria	Bacteroidetes	*Cytophaga*	0.127089934	0.059567338
Bacteria	Proteobacteria	*Dechloromonas*	0.035526581	0.054062825
Bacteria	Deferribacteres	*Deferribacter*	0.026370246	0.024204285
Bacteria	Chloroflexi	*Dehalococcoides*	0.04504917	0.033715892
Bacteria	Chloroflexi	*Dehalogenimonas*	0.014650136	0.014716636
Bacteria	Deinococcus-Thermus	*Deinococcus*	0.082407017	0.083993241
Bacteria	Proteobacteria	*Delftia*	0.017946417	0.031014548
Bacteria	Deferribacteres	*Denitrovibrio*	0.026370246	0.029930416
Bacteria	Actinobacteria	*Dermacoccus*	0.001831267	0.003587817
Bacteria	Proteobacteria	*Desulfarculus*	0.023440218	0.017717465
Bacteria	Proteobacteria	*Desulfatibacillum*	0.068123134	0.050355215
Bacteria	Firmicutes	*Desulfitobacterium*	0.149797645	0.15504878
Bacteria	Proteobacteria	*Desulfobacterium*	0.045781676	0.035728423
Bacteria	Proteobacteria	*Desulfococcus*	0.057868039	0.04895962
Bacteria	Proteobacteria	*Desulfohalobium*	0.101085941	0.060118388
Bacteria	Proteobacteria	*Desulfomicrobium*	0.090464592	0.04783955
Bacteria	Proteobacteria	*Desulfonatronospira*	0.513853535	0.267097668
Bacteria	Proteobacteria	*Desulfotalea*	0.044316663	0.036602916
Bacteria	Firmicutes	*Desulfotomaculum*	0.271760031	0.242839476
Bacteria	Proteobacteria	*Desulfovibrio*	0.405076272	0.254237831
Bacteria	Chrysiogenetes	*Desulfurispirillum*	0.030399033	0.026426455
Bacteria	Proteobacteria	*Desulfurivibrio*	0.06116432	0.049816144
Bacteria	Proteobacteria	*Desulfuromonas*	0.034061567	0.036435205
Bacteria	Firmicutes	*Dethiobacter*	0.102550955	0.086508905
Bacteria	Synergistetes	*Dethiosulfovibrio*	0.046514183	0.040202712
Bacteria	Firmicutes	*Dialister*	0.005860055	0.011116841
Bacteria	Proteobacteria	*Dichelobacter*	0.008790082	0.022161805
Bacteria	Proteobacteria	*Dickeya*	0.020510191	0.032763533
Bacteria	Dictyoglomi	*Dictyoglomus*	0.054938012	0.048636177
Bacteria	Proteobacteria	*Dinoroseobacter*	0.207665684	0.102100035
Bacteria	Bacteroidetes	*Dokdonia*	0.041386635	0.020688344
Bacteria	Firmicutes	*Dorea*	0.01391763	0.010973088
Bacteria	Bacteroidetes	*Dyadobacter*	0.074715696	0.036099783

Bacteria	Proteobacteria	*Edwardsiella*	0.008057575	0.022766762
Bacteria	Actinobacteria	*Eggerthella*	0.015748897	0.010368131
Bacteria	Proteobacteria	*Ehrlichia*	0.003662534	0.005450606
Bacteria	Proteobacteria	*Eikenella*	0.002930027	0.008373568
Bacteria	Elusimicrobia	*Elusimicrobium*	0.007325068	0.00648682
Bacteria	Proteobacteria	*Endoriftia*	0.009888842	0.021371168
Bacteria	Proteobacteria	*Enhydrobacter*	0.004028788	0.009373845
Bacteria	Proteobacteria	*Enterobacter*	0.019777684	0.027462669
Bacteria	Firmicutes	*Enterococcus*	0.047612943	0.050696626
Bacteria	Firmicutes	*Epulopiscium*	0.005860055	0.007217561
Bacteria	Firmicutes	*Eremococcus*	0.003662534	0.00222217
Bacteria	Proteobacteria	*Erwinia*	0.01501639	0.02002948
Bacteria	Firmicutes	*Erysipelothrix*	0.005860055	0.003557868
Bacteria	Proteobacteria	*Erythrobacter*	0.043584156	0.052325819
Bacteria	Proteobacteria	*Escherichia*	0.088267072	0.096942923
Bacteria	Firmicutes	*Ethanoligenens*	0.010621349	0.01297963
Bacteria	Firmicutes	*Eubacterium*	0.088267072	0.085808113
Bacteria	Firmicutes	*Exiguobacterium*	0.062995587	0.04989401
Bacteria	Firmicutes	*Faecalibacterium*	0.007325068	0.008463414
Bacteria	Proteobacteria	*Ferrimonas*	0.020143938	0.034560436
Bacteria	Thermotogae	*Fervidobacterium*	0.046514183	0.04023865
Bacteria	Fibrobacteres	*Fibrobacter*	0.01611515	0.010697563
Bacteria	Firmicutes	*Filifactor*	0.006592561	0.008325651
Bacteria	Firmicutes	*Finegoldia*	0.011720109	0.015866654
Bacteria	Bacteroidetes	*Flavobacterium*	0.154925193	0.07603296
Bacteria	Proteobacteria	*Fluoribacter*	0	5.98968E-06
Bacteria	Proteobacteria	*Francisella*	0.021608951	0.032997131
Bacteria	Actinobacteria	*Frankia*	0.082040764	0.074463665
Bacteria	Proteobacteria	*Fulvimarina*	0.014650136	0.020502664
Bacteria	Fusobacteria	*Fusobacterium*	0.106213489	0.102243787
Bacteria	Proteobacteria	*Gallionella*	0.009156335	0.013979906
Bacteria	Actinobacteria	*Gardnerella*	0.004761294	0.00241983
Bacteria	Firmicutes	*Gemella*	0.002930027	0.005133153
Bacteria	Planctomycetes	*Gemmata*	0.021975205	0.017591681
Bacteria	Gemmatimonadetes	*Gemmatimonas*	0.042119142	0.03987328
Bacteria	Firmicutes	*Geobacillus*	0.274323805	0.202852392
Bacteria	Proteobacteria	*Geobacter*	0.322303001	0.282167695
Bacteria	Actinobacteria	*Geodermatophilus*	0.020876444	0.020742252
Bacteria	Proteobacteria	*Glaciecola*	0.000366253	3.59381E-05
Bacteria	Cyanobacteria	*Gloeobacter*	0.044682916	0.047330428
Bacteria	Proteobacteria	*Gluconacetobacter*	0.013551376	0.014962213
Bacteria	Proteobacteria	*Gluconobacter*	0.006958815	0.009990781
Bacteria	Actinobacteria	*Gordonia*	0.005860055	0.007816529
Bacteria	Bacteroidetes	*Gramella*	0.201073122	0.099374732

Bacteria	Proteobacteria	*Granulibacter*	0.015382643	0.016525519
Bacteria	Firmicutes	*Granulicatella*	0.005493801	0.008145961
Bacteria	Proteobacteria	*Haemophilus*	0.028201513	0.044593145
Bacteria	Proteobacteria	*Hahella*	0.084238284	0.147513766
Bacteria	Firmicutes	*Halanaerobium*	1.53020675	3.70283631
Bacteria	Proteobacteria	*Haliangium*	0.056769279	0.054697731
Bacteria	Proteobacteria	*Halomonas*	0.95995019	0.472292024
Bacteria	Proteobacteria	*Halorhodospira*	0.388594869	0.957084503
Bacteria	Firmicutes	*Halothermothrix*	1.251121651	1.184452643
Bacteria	Proteobacteria	*Halothiobacillus*	0.023073965	0.069929479
Bacteria	Proteobacteria	*Helicobacter*	0.024538979	0.029451242
Bacteria	Firmicutes	*Heliobacterium*	0.073616936	0.065431232
Bacteria	Proteobacteria	*Herbaspirillum*	0.01391763	0.019855779
Bacteria	Proteobacteria	*Herminiimonas*	0.022707711	0.030343704
Bacteria	Chloroflexi	*Herpetosiphon*	0.058600546	0.039621714
Bacteria	Proteobacteria	*Hirschia*	0.009156335	0.007984239
Bacteria	Proteobacteria	*Histophilus*	0.009888842	0.014159597
Bacteria	Proteobacteria	*Hoeflea*	0.021608951	0.018855503
Bacteria	Firmicutes	*Holdemania*	0.023806472	0.011943416
Bacteria	Aquificae	*Hydrogenivirga*	0.011353856	0.015459356
Bacteria	Aquificae	*Hydrogenobacter*	0.010987602	0.008996495
Bacteria	Aquificae	*Hydrogenobaculum*	0.007325068	0.007822518
Bacteria	Proteobacteria	*Hyphomicrobium*	0.015382643	0.009056392
Bacteria	Proteobacteria	*Hyphomonas*	0.01721391	0.022718845
Bacteria	Proteobacteria	*Idiomarina*	0.06226308	0.115457014
Bacteria	Fusobacteria	*Ilyobacter*	0.052007984	0.037369595
Bacteria	Actinobacteria	*Intrasporangium*	0.005127548	0.007798559
Bacteria	Actinobacteria	*Janibacter*	0.006226308	0.007205581
Bacteria	Proteobacteria	*Jannaschia*	0.151628912	0.097517932
Bacteria	Proteobacteria	*Janthinobacterium*	0.03003278	0.032529936
Bacteria	Actinobacteria	*Jonesia*	0.008057575	0.006552707
Bacteria	Synergistetes	*Jonquetella*	0.007325068	0.005654255
Bacteria	Proteobacteria	*Kangiella*	0.021608951	0.049031496
Bacteria	Proteobacteria	*Ketogulonicigenium*	0.027835259	0.02447382
Bacteria	Actinobacteria	*Kineococcus*	0.023440218	0.016567447
Bacteria	Proteobacteria	*Kingella*	0.002563774	0.005133153
Bacteria	Proteobacteria	*Klebsiella*	0.03332906	0.037046152
Bacteria	Actinobacteria	*Kocuria*	0.006958815	0.006977974
Bacteria	Bacteroidetes	*Kordia*	0.029666526	0.016010407
Bacteria	Thermotogae	*Kosmotoga*	0.018312671	0.02466549
Bacteria	Actinobacteria	*Kribbella*	0.016847657	0.015058048
Bacteria	Chloroflexi	*Ktedonobacter*	0.048711704	0.031559608
Bacteria	Actinobacteria	*Kytococcus*	0.007691322	0.007546993
Bacteria	Proteobacteria	*Labrenzia*	0.049810464	0.044485331

Bacteria	Firmicutes	*Lactobacillus*	0.101452195	0.121931855
Bacteria	Firmicutes	*Lactococcus*	0.022341458	0.017148445
Bacteria	Proteobacteria	*Laribacter*	0.010255096	0.027372824
Bacteria	Proteobacteria	*Lawsonia*	0.010255096	0.01297963
Bacteria	Bacteroidetes	*Leadbetterella*	0.04614793	0.022760773
Bacteria	Bacteroidetes	*Leeuwenhoekiella*	0.059699306	0.03320078
Bacteria	Proteobacteria	*Legionella*	0.039189115	0.088359716
Bacteria	Actinobacteria	*Leifsonia*	0.003662534	0.006373016
Bacteria	Lentisphaerae	*Lentisphaera*	0.011720109	0.011554087
Bacteria	Cyanobacteria	*Leptolyngbya*	0	2.39587E-05
Bacteria	Spirochaetes	*Leptospira*	0.082040764	0.047593974
Bacteria	Nitrospirae	*Leptospirillum*	0	5.39071E-05
Bacteria	Proteobacteria	*Leptothrix*	0.011720109	0.026737918
Bacteria	Fusobacteria	*Leptotrichia*	0.021608951	0.032751554
Bacteria	Firmicutes	*Leuconostoc*	0.008057575	0.01223691
Bacteria	Proteobacteria	*Limnobacter*	0.004761294	0.02040084
Bacteria	Firmicutes	*Listeria*	0.077645723	0.06676693
Bacteria	Proteobacteria	*Loktanella*	0.06226308	0.056668334
Bacteria	Proteobacteria	*Lutiella*	0.016481403	0.026935578
Bacteria	Cyanobacteria	*Lyngbya*	0.034794074	0.027031412
Bacteria	Firmicutes	*Lysinibacillus*	0.028201513	0.025156644
Bacteria	Firmicutes	*Macrococcus*	0.008790082	0.010188441
Bacteria	Proteobacteria	*Magnetococcus*	0.070320655	0.080549177
Bacteria	Proteobacteria	*Magnetospirillum*	0.068123134	0.06786903
Bacteria	Proteobacteria	*Mannheimia*	0.003296281	0.005923791
Bacteria	Bacteroidetes	*Maribacter*	0.062629333	0.040310526
Bacteria	Proteobacteria	*Maricaulis*	0.021975205	0.025671756
Bacteria	Proteobacteria	*Marinobacter*	0.352335781	0.498898169
Bacteria	Proteobacteria	*Marinomonas*	0.065925614	0.098811702
Bacteria	Proteobacteria	*Mariprofundus*	0.010255096	0.018238567
Bacteria	Proteobacteria	*Maritimibacter*	0.078011976	0.048097107
Bacteria	Bacteroidetes	*Marivirga*	0.151262659	0.061729611
Bacteria	Firmicutes	*Megasphaera*	0.006226308	0.010877253
Bacteria	Deinococcus-Thermus	*Meiothermus*	0.082040764	0.066683074
Bacteria	Tenericutes	*Mesoplasma*	0.001465014	0.002928952
Bacteria	Proteobacteria	*Mesorhizobium*	0.071053162	0.067515639
Bacteria	Verrucomicrobia	*Methylacidiphilum*	0.006958815	0.007894394
Bacteria	Proteobacteria	*Methylibium*	0.020510191	0.047168707
Bacteria	Proteobacteria	*Methylobacillus*	0.021242698	0.046437966
Bacteria	Proteobacteria	*Methylobacter*	0.024905232	0.044671011
Bacteria	Proteobacteria	*Methylobacterium*	0.111341037	0.115343211
Bacteria	Proteobacteria	*Methylocella*	0.01501639	0.019388585
Bacteria	Proteobacteria	*Methylococcus*	0.072884429	0.175329826
Bacteria	Proteobacteria	*Methylophaga*	0.014650136	0.056632396

Bacteria	Proteobacteria	*Methylosinus*	0.007325068	0.010649646
Bacteria	Proteobacteria	*Methylotenera*	0.007325068	0.022371444
Bacteria	Proteobacteria	*Methylovorus*	0.012452616	0.023030308
Bacteria	Actinobacteria	*Micrococcus*	0.007691322	0.009954843
Bacteria	Cyanobacteria	*Microcoleus*	0.04724669	0.038369871
Bacteria	Cyanobacteria	*Microcystis*	0.028934019	0.022059981
Bacteria	Actinobacteria	*Micromonospora*	0.026003992	0.017136466
Bacteria	Bacteroidetes	*Microscilla*	0.09559214	0.046515832
Bacteria	Firmicutes	*Mitsuokella*	0.008790082	0.015866654
Bacteria	Actinobacteria	*Mobiluncus*	0.002930027	0.00722355
Bacteria	Firmicutes	*Moorella*	0.183126705	0.1416918
Bacteria	Proteobacteria	*Moraxella*	0.004395041	0.008505341
Bacteria	Proteobacteria	*Moritella*	0.004028788	0.013638495
Bacteria	Bacteroidetes	*Mucilaginibacter*	0.036259088	0.019017225
Bacteria	Actinobacteria	*Mycobacterium*	0.156390206	0.123261564
Bacteria	Tenericutes	*Mycoplasma*	0.027102752	0.031493722
Bacteria	Proteobacteria	*Myxococcus*	0.082773271	0.069719841
Bacteria	Actinobacteria	*Nakamurella*	0.01501639	0.015357532
Bacteria	Firmicutes	*Natranaerobius*	0.262969949	0.204086265
Bacteria	Proteobacteria	*Nautilia*	0.007325068	0.006564686
Bacteria	Proteobacteria	*Neisseria*	0.027102752	0.058495186
Bacteria	Proteobacteria	*Neorickettsia*	0	0.001587264
Bacteria	Proteobacteria	*Neptuniibacter*	0.025637739	0.038561541
Bacteria	Proteobacteria	*Nitratiruptor*	0.020510191	0.015076017
Bacteria	Proteobacteria	*Nitrobacter*	0.055670518	0.043718653
Bacteria	Proteobacteria	*Nitrococcus*	0.324500522	1.139050891
Bacteria	Proteobacteria	*Nitrosococcus*	0.213891992	0.428088207
Bacteria	Proteobacteria	*Nitrosomonas*	0.056403025	0.098925506
Bacteria	Proteobacteria	*Nitrosospira*	0.020876444	0.048145024
Bacteria	Nitrospirae	*Nitrospira*	0.018678924	0.018052887
Bacteria	Actinobacteria	*Nocardia*	0.018312671	0.017022662
Bacteria	Actinobacteria	*Nocardioides*	0.032962807	0.029475201
Bacteria	Actinobacteria	*Nocardiopsis*	0.026736499	0.017711475
Bacteria	Cyanobacteria	*Nodularia*	0.025637739	0.02040683
Bacteria	Cyanobacteria	*Nostoc*	0.104382222	0.080579125
Bacteria	Proteobacteria	*Novosphingobium*	0.012086363	0.020526623
Bacteria	Proteobacteria	*Oceanibulbus*	0.030399033	0.020688344
Bacteria	Proteobacteria	*Oceanicaulis*	0.014283883	0.021215436
Bacteria	Proteobacteria	*Oceanicola*	0.138810043	0.073667038
Bacteria	Deinococcus-Thermus	*Oceanithermus*	0.016847657	0.01742397
Bacteria	Firmicutes	*Oceanobacillus*	0.081308257	0.075224354
Bacteria	Proteobacteria	*Ochrobactrum*	0.03332906	0.037974552
Bacteria	Proteobacteria	*Octadecabacter*	0.052007984	0.046599687
Bacteria	Firmicutes	*Oenococcus*	0.00219752	0.004917525

Bacteria	Proteobacteria	*Oligotropha*	0.010621349	0.010224379
Bacteria	Actinobacteria	*Olsenella*	0.004028788	0.003222446
Bacteria	Verrucomicrobia	*Opitutus*	0.027102752	0.0283731
Bacteria	Firmicutes	*Oribacterium*	0.008790082	0.009301968
Bacteria	Proteobacteria	*Orientia*	0.00219752	0.00240785
Bacteria	Cyanobacteria	*Oscillatoria*	0.032596554	0.015279666
Bacteria	Chloroflexi	*Oscillochloris*	0.020510191	0.017837258
Bacteria	Proteobacteria	*Oxalobacter*	0.006226308	0.015111955
Bacteria	Firmicutes	*Paenibacillus*	0.189719267	0.158408988
Bacteria	Bacteroidetes	*Paludibacter*	0.029300273	0.013213228
Bacteria	Proteobacteria	*Pantoea*	0.024905232	0.029247593
Bacteria	Bacteroidetes	*Parabacteroides*	0.055670518	0.029828592
Bacteria	Chlamydiae	*Parachlamydia*	0.003296281	0.003767507
Bacteria	Proteobacteria	*Paracoccus*	0.133316242	0.082681502
Bacteria	Actinobacteria	*Parascardovia*	0.000366253	0.001593254
Bacteria	Proteobacteria	*Parvibaculum*	0.0322303	0.049037486
Bacteria	Proteobacteria	*Parvularcula*	0.005860055	0.016333849
Bacteria	Proteobacteria	*Pasteurella*	0.012086363	0.017741423
Bacteria	Proteobacteria	*Pectobacterium*	0.028567766	0.033290625
Bacteria	Firmicutes	*Pediococcus*	0.010621349	0.011643932
Bacteria	Bacteroidetes	*Pedobacter*	0.149431392	0.062286651
Bacteria	Proteobacteria	*Pelagibaca*	0.050176717	0.030475477
Bacteria	Proteobacteria	*Pelobacter*	0.130752468	0.130089795
Bacteria	Chlorobi	*Pelodictyon*	0.065193107	0.050367194
Bacteria	Firmicutes	*Pelotomaculum*	0.176534144	0.134222673
Bacteria	Firmicutes	*Peptoniphilus*	0.021975205	0.021407106
Bacteria	Firmicutes	*Peptostreptococcus*	0.012086363	0.015279666
Bacteria	Aquificae	*Persephonella*	0.016847657	0.013153331
Bacteria	Thermotogae	*Petrotoga*	0.098888421	0.100842202
Bacteria	Proteobacteria	*Phaeobacter*	0.01611515	0.014824451
Bacteria	Proteobacteria	*Phenylobacterium*	0.009888842	0.015531233
Bacteria	Proteobacteria	*Photobacterium*	0.046514183	0.076847556
Bacteria	Proteobacteria	*Photorhabdus*	0.017580164	0.027181154
Bacteria	Planctomycetes	*Pirellula*	0.016847657	0.016028376
Bacteria	Planctomycetes	*Planctomyces*	0.054938012	0.053613599
Bacteria	Proteobacteria	*Plesiocystis*	0.029300273	0.028594718
Bacteria	Bacteroidetes	*Polaribacter*	0.085703298	0.041927739
Bacteria	Proteobacteria	*Polaromonas*	0.063728093	0.07122325
Bacteria	Proteobacteria	*Polynucleobacter*	0.009888842	0.02132924
Bacteria	Bacteroidetes	*Porphyromonas*	0.052007984	0.034967735
Bacteria	Bacteroidetes	*Prevotella*	0.079843244	0.050211463
Bacteria	Cyanobacteria	*Prochlorococcus*	0.036625341	0.047132769
Bacteria	Actinobacteria	*Propionibacterium*	0.005493801	0.019747965
Bacteria	Chlorobi	*Prosthecochloris*	0.023440218	0.018406278

Bacteria	Proteobacteria	*Proteus*	0.021975205	0.02799575
Bacteria	Proteobacteria	*Providencia*	0.01501639	0.01817867
Bacteria	Proteobacteria	*Pseudoalteromonas*	0.089365832	0.123932407
Bacteria	Proteobacteria	*Pseudomonas*	0.528869925	0.72549963
Bacteria	Firmicutes	*Pseudoramibacter*	0.004761294	0.003821414
Bacteria	Proteobacteria	*Pseudovibrio*	0.014650136	0.01521378
Bacteria	Proteobacteria	*Psychrobacter*	0.032962807	0.059459524
Bacteria	Bacteroidetes	*Psychroflexus*	0.059333053	0.042778273
Bacteria	Proteobacteria	*Psychromonas*	0.038090355	0.04709683
Bacteria	Synergistetes	*Pyramidobacter*	0.006958815	0.007894394
Bacteria	Proteobacteria	*Ralstonia*	0.056769279	0.082316131
Bacteria	Cyanobacteria	*Raphidiopsis*	0.010987602	0.005833945
Bacteria	Proteobacteria	*Reinekea*	0.042119142	0.057716528
Bacteria	Actinobacteria	*Renibacterium*	0.002563774	0.003114632
Bacteria	Proteobacteria	*Rhizobium*	0.102550955	0.094127775
Bacteria	Proteobacteria	*Rhodobacter*	0.633252147	0.315434361
Bacteria	Actinobacteria	*Rhodococcus*	0.055304265	0.055392533
Bacteria	Proteobacteria	*Rhodomicrobium*	0.016481403	0.015872644
Bacteria	Planctomycetes	*Rhodopirellula*	0.047612943	0.050732564
Bacteria	Proteobacteria	*Rhodopseudomonas*	0.130019961	0.125537641
Bacteria	Proteobacteria	*Rhodospirillum*	0.07947699	0.109467337
Bacteria	Bacteroidetes	*Rhodothermus*	1.052246049	1.188693335
Bacteria	Proteobacteria	*Rickettsia*	0.015748897	0.017735434
Bacteria	Proteobacteria	*Rickettsiella*	0.001831267	0.008301692
Bacteria	Bacteroidetes	*Riemerella*	0.020143938	0.00816393
Bacteria	Bacteroidetes	*Robiginitalea*	0.067390628	0.048001272
Bacteria	Firmicutes	*Roseburia*	0.011720109	0.011949406
Bacteria	Proteobacteria	*Roseibium*	0.014650136	0.01779533
Bacteria	Chloroflexi	*Roseiflexus*	0.204369403	0.147939033
Bacteria	Proteobacteria	*Roseobacter*	0.39518743	0.258622275
Bacteria	Proteobacteria	*Roseomonas*	0.015748897	0.01577082
Bacteria	Proteobacteria	*Roseovarius*	0.193748054	0.131635132
Bacteria	Actinobacteria	*Rothia*	0.004761294	0.007355323
Bacteria	Actinobacteria	*Rubrobacter*	0.138077536	0.100177348
Bacteria	Proteobacteria	*Ruegeria*	0.395553684	0.255058417
Bacteria	Firmicutes	*Ruminococcus*	0.04504917	0.045389773
Bacteria	Actinobacteria	*Saccharomonospora*	0.010621349	0.012117117
Bacteria	Proteobacteria	*Saccharophagus*	0.04614793	0.070276881
Bacteria	Actinobacteria	*Saccharopolyspora*	0.040654129	0.041011319
Bacteria	Proteobacteria	*Sagittula*	0.035160327	0.025504045
Bacteria	Bacteroidetes	*Salinibacter*	3.163696962	4.10990674
Bacteria	Actinobacteria	*Salinispora*	0.028201513	0.030116096
Bacteria	Proteobacteria	*Salmonella*	0.041020382	0.041766018
Bacteria	Actinobacteria	*Sanguibacter*	0.008423828	0.009218113

Bacteria	Actinobacteria	*Scardovia*	0.001465014	0.001239863
Bacteria	Fusobacteria	*Sebaldella*	0.036991594	0.038262057
Bacteria	Actinobacteria	*Segniliparus*	0.004761294	0.003731569
Bacteria	Firmicutes	*Selenomonas*	0.040654129	0.042161337
Bacteria	Proteobacteria	*Serratia*	0.023440218	0.036129732
Bacteria	Proteobacteria	*Shewanella*	0.217554526	0.3089715
Bacteria	Proteobacteria	*Shigella*	0.007325068	0.013818185
Bacteria	Firmicutes	*Shuttleworthia*	0.003296281	0.003785476
Bacteria	Proteobacteria	*Sideroxydans*	0.010255096	0.021730548
Bacteria	Proteobacteria	*Simonsiella*	0.003296281	0.004432361
Bacteria	Proteobacteria	*Sinorhizobium*	0.115369824	0.088533416
Bacteria	Actinobacteria	*Slackia*	0.008790082	0.012512435
Bacteria	Proteobacteria	*Sodalis*	0.006226308	0.015291645
Bacteria	Proteobacteria	*Sorangium*	0.039921622	0.032434101
Bacteria	Chloroflexi	*Sphaerobacter*	0.097057154	0.065395294
Bacteria	Bacteroidetes	*Sphingobacterium*	0.043584156	0.023509482
Bacteria	Proteobacteria	*Sphingobium*	0.010255096	0.015710923
Bacteria	Proteobacteria	*Sphingomonas*	0.029300273	0.033997407
Bacteria	Proteobacteria	*Sphingopyxis*	0.018312671	0.015716913
Bacteria	Spirochaetes	*Spirochaeta*	0.491145824	0.135210969
Bacteria	Tenericutes	*Spiroplasma*	0	1.19794E-05
Bacteria	Bacteroidetes	*Spirosoma*	0.098888421	0.057087612
Bacteria	Actinobacteria	*Stackebrandtia*	0.019777684	0.017891165
Bacteria	Firmicutes	*Staphylococcus*	0.115003571	0.123537089
Bacteria	Proteobacteria	*Starkeya*	0.023440218	0.029726767
Bacteria	Proteobacteria	*Stenotrophomonas*	0.026736499	0.058908474
Bacteria	Proteobacteria	*Stigmatella*	0.03332906	0.027408762
Bacteria	Fusobacteria	*Streptobacillus*	0.008790082	0.007870436
Bacteria	Firmicutes	*Streptococcus*	0.07947699	0.091366533
Bacteria	Actinobacteria	*Streptomyces*	0.204735657	0.180031723
Bacteria	Actinobacteria	*Streptosporangium*	0.024172725	0.016226035
Bacteria	Firmicutes	*Subdoligranulum*	0.006592561	0.003857352
Bacteria	Proteobacteria	*Sulfitobacter*	0.045415423	0.042502748
Bacteria	Proteobacteria	*Sulfuricurvum*	0.004395041	0.005402689
Bacteria	Aquificae	*Sulfurihydrogenibium*	0.024172725	0.024599604
Bacteria	Proteobacteria	*Sulfurimonas*	0.01611515	0.013848133
Bacteria	Proteobacteria	*Sulfurospirillum*	0.005493801	0.006145409
Bacteria	Proteobacteria	*Sulfurovum*	0.019777684	0.015489305
Bacteria	Firmicutes	*Symbiobacterium*	0.11170729	0.090731628
Bacteria	Cyanobacteria	*Synechococcus*	0.253813614	0.238323259
Bacteria	Cyanobacteria	*Synechocystis*	0.068489388	0.053481826
Bacteria	Proteobacteria	*Syntrophobacter*	0.087168312	0.057722518
Bacteria	Firmicutes	*Syntrophomonas*	0.127089934	0.092989736
Bacteria	Firmicutes	*Syntrophothermus*	0.028201513	0.028834305

Bacteria	Proteobacteria	*Syntrophus*	0.07727947	0.063676257
Bacteria	Proteobacteria	*Teredinibacter*	0.020510191	0.034452622
Bacteria	Acidobacteria	*Terriglobus*	0.015382643	0.010583759
Bacteria	Firmicutes	*Tetragenococcus*	0	1.19794E-05
Bacteria	Proteobacteria	*Thalassobium*	0.018312671	0.022113888
Bacteria	Proteobacteria	*Thauera*	0.038822862	0.053715424
Bacteria	Firmicutes	*Thermaerobacter*	0.0322303	0.031883051
Bacteria	Synergistetes	*Thermanaerovibrio*	0.022707711	0.02114356
Bacteria	Firmicutes	*Thermincola*	0.116468585	0.088898786
Bacteria	Firmicutes	*Thermoanaerobacter*	0.183126705	0.138924569
Bacteria	Firmicutes	*Thermoanaerobacterium*	0.056403025	0.050355215
Bacteria	unclassified (derived from Bacteria)	*Thermobaculum*	0.049810464	0.035770351
Bacteria	Actinobacteria	*Thermobifida*	0.025637739	0.02206597
Bacteria	Actinobacteria	*Thermobispora*	0.014650136	0.014938255
Bacteria	Aquificae	*Thermocrinis*	0.010621349	0.008822794
Bacteria	Nitrospirae	*Thermodesulfovibrio*	0.025271485	0.020820117
Bacteria	Chloroflexi	*Thermomicrobium*	0.037724101	0.029139779
Bacteria	Actinobacteria	*Thermomonospora*	0.01611515	0.016807034
Bacteria	Firmicutes	*Thermosediminibacter*	0.054205505	0.04600072
Bacteria	Firmicutes	*Thermosinus*	0.085703298	0.058836597
Bacteria	Thermotogae	*Thermosipho*	0.065925614	0.060938974
Bacteria	Cyanobacteria	*Thermosynechococcus*	0.045781676	0.037872728
Bacteria	Thermotogae	*Thermotoga*	0.137711282	0.106993601
Bacteria	Deinococcus-Thermus	*Thermus*	0.07727947	0.08734147
Bacteria	Proteobacteria	*Thioalkalivibrio*	0.239529731	0.589156612
Bacteria	Proteobacteria	*Thiobacillus*	0.026736499	0.070013335
Bacteria	Proteobacteria	*Thiomicrospira*	0.024172725	0.06861175
Bacteria	Proteobacteria	*Thiomonas*	0.014283883	0.021197467
Bacteria	Proteobacteria	*Tolumonas*	0.01391763	0.024827211
Bacteria	Spirochaetes	*Treponema*	0.278352592	0.081465597
Bacteria	Cyanobacteria	*Trichodesmium*	0.070320655	0.051313563
Bacteria	Actinobacteria	*Tropheryma*	0.002563774	0.001425543
Bacteria	Deinococcus-Thermus	*Truepera*	0.093760873	0.087838614
Bacteria	Actinobacteria	*Tsukamurella*	0.006226308	0.005720142
Bacteria	Firmicutes	*Turicibacter*	0.051641731	0.017507826
Bacteria	Tenericutes	*Ureaplasma*	0.00109876	0.001940655
Bacteria	Proteobacteria	*Variovorax*	0.014283883	0.01762163
Bacteria	Firmicutes	*Veillonella*	0.024538979	0.033069007
Bacteria	Proteobacteria	*Verminephrobacter*	0.039555368	0.043065778
Bacteria	Verrucomicrobia	*Verrucomicrobium*	0.020143938	0.014812471
Bacteria	Proteobacteria	*Vibrio*	0.208398191	0.275998328
Bacteria	Lentisphaerae	*Victivallis*	0.011720109	0.009709266
Bacteria	Chlamydiae	*Waddlia*	0.006958815	0.005630296
Bacteria	Firmicutes	*Weissella*	0.004028788	0.002725303

Bacteria	Proteobacteria	*Wigglesworthia*	0.00109876	0.00167112
Bacteria	Proteobacteria	*Wolbachia*	0.006958815	0.00575009
Bacteria	Proteobacteria	*Wolinella*	0.019411431	0.016741147
Bacteria	Proteobacteria	*Xanthobacter*	0.04504917	0.040268599
Bacteria	Proteobacteria	*Xanthomonas*	0.094127127	0.206356353
Bacteria	Proteobacteria	*Xenorhabdus*	0.005127548	0.011110851
Bacteria	Actinobacteria	*Xylanimonas*	0.009888842	0.00870899
Bacteria	Proteobacteria	*Xylella*	0.016481403	0.078338986
Bacteria	Proteobacteria	*Yersinia*	0.037357848	0.062280662
Bacteria	Bacteroidetes	*Zunongwangia*	0.070686908	0.036519061
Bacteria	Proteobacteria	*Zymomonas*	0.009522589	0.011068923
Bacteria	Actinobacteria	Unclassified Actinobacteria	0.005127548	0.005678214
Bacteria	Firmicutes	Unclassified *Alicyclobacillaceae*	0.036625341	0.040448289
Bacteria	Proteobacteria	Unclassified Alphaproteobacteria	0.026370246	0.039471972
Bacteria	Proteobacteria	Unclassified *Alteromonadales*	0.011353856	0.012650198
Bacteria	unclassified (derived from Bacteria)	Unclassified Bacteria	0.005493801	0.002910983
Bacteria	Bacteroidetes	Unclassified Bacteroidetes	0.027835259	0.01316531
Bacteria	Proteobacteria	Unclassified Betaproteobacteria	0.000366253	0.004606062
Bacteria	Proteobacteria	Unclassified *Burkholderiales*	0.002563774	0.005672224
Bacteria	Proteobacteria	Unclassified *Campylobacterales*	0.010621349	0.005600348
Bacteria	Candidatus Poribacteria	Unclassified Candidatus Poribacteria	0.012086363	0.01391402
Bacteria	Cyanobacteria	Unclassified *Chroococcales*	0.00109876	0.001952635
Bacteria	Firmicutes	Unclassified *Clostridiales* Family XI. Incertae Sedis)	0.005860055	0.006355047
Bacteria	Firmicutes	Unclassified *Clostridiales*	0.010987602	0.017723454
Bacteria	Proteobacteria	Unclassified Deltaproteobacteria	0.097423407	0.071906073
Bacteria	Elusimicrobia	Unclassified Elusimicrobia	0.003662534	0.004833669
Bacteria	Firmicutes	Unclassified *Erysipelotrichaceae*	0.035892834	0.025174613
Bacteria	Bacteroidetes	Unclassified Flavobacteria	0.061896826	0.035440919
Bacteria	Bacteroidetes	Unclassified *Flavobacteriaceae*	0.019777684	0.011889509
Bacteria	Bacteroidetes	Unclassified *Flavobacteriales*	0.068855641	0.035339094
Bacteria	Proteobacteria	Unclassified Gammaproteobacteria	0.115369824	0.248092423
Bacteria	Firmicutes	Unclassified *Lachnospiraceae*	0.007325068	0.007864446
Bacteria	Proteobacteria	Unclassified *Methylophilales*	0.004028788	0.004929504
Bacteria	Verrucomicrobia	Unclassified *Opitutaceae*	0.008790082	0.009547545
Bacteria	Proteobacteria	Unclassified Proteobacteria	0.000732507	0.000161721
Bacteria	Proteobacteria	Unclassified *Rhodobacteraceae*	0.053472998	0.042394934
Bacteria	Proteobacteria	Unclassified *Rhodobacterales*	0.03003278	0.033266666
Bacteria	Proteobacteria	Unclassified *Rickettsiales*	0.00219752	0.00351594
Bacteria	Firmicutes	Unclassified *Ruminococcaceae*	0.005493801	0.004402413
Bacteria	Thermotogae	Unclassified *Thermotogales*	0.022341458	0.014477049
Bacteria	Verrucomicrobia	Unclassified Verrucomicrobia subdivision 3	0.013551376	0.01539946
Bacteria	Verrucomicrobia	Unclassified *Verrucomicrobiales*	0.027835259	0.022736814
Bacteria	Proteobacteria	Unclassified Vibrionaceae	0.013185123	0.016819013

Bacteria	Proteobacteria	*Unclassified Vibrionales*	0.009888842	0.007774601
Eukaryota	unclassified (derived from Eukaryota)	*Acanthamoeba*	0	6.58864E-05
Eukaryota	Chordata	*Acheilognathus*	0	5.98968E-06
Eukaryota	Arthropoda	*Acyrthosiphon*	0.005127548	0.001880759
Eukaryota	Streptophyta	*Adiantum*	0	5.39071E-05
Eukaryota	Arthropoda	*Aedes*	0.003296281	0.002755251
Eukaryota	Streptophyta	*Aethionema*	0	5.98968E-06
Eukaryota	Streptophyta	*Agrostis*	0	2.99484E-05
Eukaryota	Chordata	*Ailuropoda*	0.000732507	0.000149742
Eukaryota	Ascomycota	*Ajellomyces*	0.001465014	0.000952359
Eukaryota	Blastocladiomycota	*Allomyces*	0	1.19794E-05
Eukaryota	Streptophyta	*Alsophila*	0	5.98968E-06
Eukaryota	Streptophyta	*Amborella*	0	2.39587E-05
Eukaryota	Streptophyta	*Aneura*	0	0.000101825
Eukaryota	Streptophyta	*Angiopteris*	0	1.7969E-05
Eukaryota	Arthropoda	*Anopheles*	0.004028788	0.004468299
Eukaryota	Streptophyta	*Anthoceros*	0	0.000521102
Eukaryota	Arthropoda	*Apis*	0.00109876	0.001796903
Eukaryota	Streptophyta	*Arabidopsis*	0.080942004	0.034021366
Eukaryota	Ascomycota	*Arthroderma*	0.000732507	0.001132049
Eukaryota	Ascomycota	*Aspergillus*	0.007325068	0.006343068
Eukaryota	Cnidaria	*Astrangia*	0	5.98968E-06
Eukaryota	Streptophyta	*Atropa*	0	7.78658E-05
Eukaryota	unclassified (derived from Eukaryota)	*Aureococcus*	0	0.000383339
Eukaryota	Apicomplexa	*Babesia*	0.001465014	0.000796627
Eukaryota	Ascomycota	*Barssia*	0	1.19794E-05
Eukaryota	Streptophyta	*Beta*	0.000366253	5.98968E-06
Eukaryota	unclassified (derived from Eukaryota)	*Bigelowiella*	0	0.00037735
Eukaryota	Blastocladiomycota	*Blastocladiella*	0	5.98968E-06
Eukaryota	unclassified (derived from Eukaryota)	*Blastocystis*	0	1.19794E-05
Eukaryota	Chytridiomycota	*Boothiomyces*	0.000366253	0
Eukaryota	Chordata	*Bos*	0.004395041	0.003599796
Eukaryota	Ascomycota	*Botryotinia*	0.00219752	0.001161997
Eukaryota	Streptophyta	*Brachypodium*	0	5.98968E-06
Eukaryota	Chordata	*Branchiostoma*	0.012452616	0.005157112
Eukaryota	Streptophyta	*Brassica*	0	5.98968E-06
Eukaryota	Nematoda	*Brugia*	0.014650136	0.143530631
Eukaryota	Chlorophyta	*Bryopsis*	0	0.000545061
Eukaryota	Streptophyta	*Buxus*	0	0.000131773
Eukaryota	Nematoda	*Caenorhabditis*	0.009156335	0.008930608
Eukaryota	unclassified (derived from Eukaryota)	*Cafeteria*	0.00109876	0.000431257

Eukaryota	Chordata	*Callithrix*	0.000366253	0.00036537
Eukaryota	Ascomycota	*Candida*	0.002930027	0.002761241
Eukaryota	Chordata	*Canis*	0.001465014	0.001042204
Eukaryota	Streptophyta	*Carica*	0	3.59381E-05
Eukaryota	Streptophyta	*Ceratophyllum*	0	0.000359381
Eukaryota	Ascomycota	*Chaetomium*	0.00219752	0.000892462
Eukaryota	Streptophyta	*Chaetosphaeridium*	0.00109876	0.001820862
Eukaryota	Streptophyta	*Chara*	0.000366253	0.000760689
Eukaryota	unclassified (derived from Eukaryota)	*Chattonella*	0	2.39587E-05
Eukaryota	Streptophyta	*Cheilanthes*	0	5.98968E-06
Eukaryota	Chlorophyta	*Chlamydomonas*	0.179830425	0.105304512
Eukaryota	Chlorophyta	*Chlorella*	0.001465014	0.003282343
Eukaryota	Streptophyta	*Chlorokybus*	0	0.001084132
Eukaryota	unclassified (derived from Eukaryota)	*Chondrus*	0.000366253	0.000215628
Eukaryota	unclassified (derived from Eukaryota)	*Chrysodidymus*	0	8.38555E-05
Eukaryota	Chordata	*Ciona*	0.002930027	0.002455768
Eukaryota	Streptophyta	*Citrus*	0	4.19277E-05
Eukaryota	Ascomycota	*Clavispora*	0.001465014	0.000832565
Eukaryota	Ascomycota	*Coccidioides*	0.001831267	0.001461481
Eukaryota	Streptophyta	*Coffea*	0	1.19794E-05
Eukaryota	Basidiomycota	*Coprinopsis*	0.00219752	0.001629192
Eukaryota	Streptophyta	*Corynocarpus*	0	5.98968E-06
Eukaryota	Streptophyta	*Cryptomeria*	0	4.79174E-05
Eukaryota	unclassified (derived from Eukaryota)	*Cryptomonas*	0	3.59381E-05
Eukaryota	Apicomplexa	*Cryptosporidium*	0.002930027	0.002204201
Eukaryota	Streptophyta	*Cucurbita*	0	5.98968E-06
Eukaryota	Arthropoda	*Culex*	0.00109876	0.001126059
Eukaryota	Streptophyta	*Cuscuta*	0	5.39071E-05
Eukaryota	unclassified (derived from Eukaryota)	*Cyanidioschyzon*	0	0.000407298
Eukaryota	unclassified (derived from Eukaryota)	*Cyanidium*	0.000732507	0.000485164
Eukaryota	unclassified (derived from Eukaryota)	*Cyanophora*	0.000366253	0.000982307
Eukaryota	Streptophyta	*Cycas*	0	5.98968E-06
Eukaryota	Chordata	*Danio*	0.008790082	0.005630296
Eukaryota	Streptophyta	*Daucus*	0	5.98968E-06
Eukaryota	Ascomycota	*Debaryomyces*	0.00219752	0.001257832
Eukaryota	Phaeophyceae	*Desmarestia*	0	1.19794E-05
Eukaryota	unclassified (derived from Eukaryota)	*Dictyostelium*	0.0322303	0.018112783
Eukaryota	Phaeophyceae	*Dictyota*	0	1.19794E-05
Eukaryota	Streptophyta	*Dioscorea*	0	2.99484E-05
Eukaryota	Streptophyta	*Draba*	0	3.59381E-05

Eukaryota	Arthropoda	*Drosophila*	0.020143938	0.013225207
Eukaryota	Chlorophyta	*Dunaliella*	0.003662534	0.010104585
Eukaryota	unclassified (derived from Eukaryota)	*Durinskia*	0	6.58864E-05
Eukaryota	Phaeophyceae	*Ectocarpus*	0	4.79174E-05
Eukaryota	Apicomplexa	*Eimeria*	0	5.98968E-06
Eukaryota	Ascomycota	*Emericella*	0.00219752	0.002755251
Eukaryota	unclassified (derived from Eukaryota)	*Emiliania*	0	0.000485164
Eukaryota	Microsporidia	*Encephalitozoon*	0.001465014	0.001156008
Eukaryota	unclassified (derived from Eukaryota)	*Entamoeba*	0.005127548	0.002737282
Eukaryota	Microsporidia	*Enterocytozoon*	0	0.001030224
Eukaryota	Streptophyta	*Epifagus*	0	5.98968E-06
Eukaryota	Streptophyta	*Equisetum*	0	0.000119794
Eukaryota	Chordata	*Equus*	0.000732507	0.00055105
Eukaryota	Ascomycota	*Eremothecium*	0.002930027	0.002168263
Eukaryota	Streptophyta	*Erodium*	0	5.98968E-06
Eukaryota	Euglenida	*Euglena*	0	0.000622926
Eukaryota	Streptophyta	*Fagopyrum*	0	5.98968E-06
Eukaryota	Basidiomycota	*Filobasidiella*	0.007691322	0.003839383
Eukaryota	Chlorophyta	*Floydiella*	0	0.001719037
Eukaryota	Phaeophyceae	*Fucus*	0.000366253	3.59381E-05
Eukaryota	Chordata	*Gallus*	0.002930027	0.002192222
Eukaryota	Streptophyta	*Geranium*	0	5.98968E-06
Eukaryota	unclassified (derived from Eukaryota)	*Giardia*	0.00109876	0.00074272
Eukaryota	Ascomycota	*Gibberella*	0.006958815	0.006139419
Eukaryota	Glomeromycota	*Glomus*	0	1.19794E-05
Eukaryota	Streptophyta	*Gnetum*	0	1.19794E-05
Eukaryota	unclassified (derived from Eukaryota)	*Gracilaria*	0.000366253	0.00037735
Eukaryota	unclassified (derived from Eukaryota)	*Gracilariophila*	0	5.98968E-06
Eukaryota	unclassified (derived from Eukaryota)	*Guillardia*	0.000732507	0.000826575
Eukaryota	Streptophyta	*Guizotia*	0	5.98968E-06
Eukaryota	Apicomplexa	*Haemoproteus*	0	5.98968E-06
Eukaryota	Chytridiomycota	*Harpochytrium*	0	5.98968E-06
Eukaryota	unclassified (derived from Eukaryota)	*Hartmannella*	0	1.7969E-05
Eukaryota	unclassified (derived from Eukaryota)	*Hemiselmis*	0.00109876	0.00019167
Eukaryota	unclassified (derived from Eukaryota)	*Heterosigma*	0	2.99484E-05
Eukaryota	Chordata	*Homo*	0.006592561	0.005893842
Eukaryota	Streptophyta	*Huperzia*	0	2.99484E-05
Eukaryota	Cnidaria	*Hydra*	0.004761294	0.003797455

Eukaryota	Ascomycota	*Hypocrea*	0	2.39587E-05
Eukaryota	Streptophyta	*Illicium*	0	5.98968E-06
Eukaryota	Streptophyta	*Ipomoea*	0	1.19794E-05
Eukaryota	Streptophyta	*Isoetes*	0	5.39071E-05
Eukaryota	Arthropoda	*Ixodes*	0.000732507	0.001629192
Eukaryota	Streptophyta	*Jasminum*	0.000366253	1.7969E-05
Eukaryota	Ascomycota	*Kluyveromyces*	0.002930027	0.002000552
Eukaryota	unclassified (derived from Eukaryota)	*Kryptoperidinium*	0	1.19794E-05
Eukaryota	Basidiomycota	*Laccaria*	0.000732507	0.001581275
Eukaryota	Ascomycota	*Lachancea*	0.000366253	0.001227884
Eukaryota	Streptophyta	*Lactuca*	0	0.000215628
Eukaryota	Phaeophyceae	*Laminaria*	0	2.99484E-05
Eukaryota	unclassified (derived from Eukaryota)	*Leishmania*	0.006592561	0.016339839
Eukaryota	Ascomycota	*Leotia*	0	0.000940379
Eukaryota	Chlorophyta	*Leptosira*	0.000732507	0.002365922
Eukaryota	Nematoda	*Loa*	0.007325068	0.045599411
Eukaryota	Streptophyta	*Lobularia*	0	1.7969E-05
Eukaryota	Arthropoda	*Locusta*	0	5.98968E-06
Eukaryota	Ascomycota	*Lodderomyces*	0.001831267	0.000892462
Eukaryota	Mollusca	*Loripes*	0	5.98968E-06
Eukaryota	Streptophyta	*Lotus*	0	8.98452E-05
Eukaryota	Chordata	*Macaca*	0.000732507	0.000844544
Eukaryota	Ascomycota	*Magnaporthe*	0.002563774	0.002198211
Eukaryota	Basidiomycota	*Malassezia*	0.001465014	0.002294046
Eukaryota	unclassified (derived from Eukaryota)	*Malawimonas*	0	3.59381E-05
Eukaryota	Streptophyta	*Marchantia*	0.000366253	0.001245853
Eukaryota	Streptophyta	*Medicago*	0	5.98968E-06
Eukaryota	Streptophyta	*Megaleranthis*	0	0.000251566
Eukaryota	Streptophyta	*Mesostigma*	0.001465014	0.002641448
Eukaryota	Cnidaria	*Metridium*	0	1.19794E-05
Eukaryota	Ascomycota	*Meyerozyma*	0.001465014	0.000808606
Eukaryota	Chlorophyta	*Micromonas*	0.019777684	0.009954843
Eukaryota	Basidiomycota	*Moniliophthora*	0.001465014	0.000994286
Eukaryota	Chordata	*Monodelphis*	0.00219752	0.001365646
Eukaryota	Chlorophyta	*Monomastix*	0	0.000604957
Eukaryota	unclassified (derived from Eukaryota)	*Monosiga*	0.006958815	0.026228796
Eukaryota	Streptophyta	*Monsonia*	0	3.59381E-05
Eukaryota	unclassified (derived from Fungi)	*Mortierella*	0	0.000143752
Eukaryota	Streptophyta	*Morus*	0	2.99484E-05
Eukaryota	Chordata	*Mus*	0.007325068	0.005169091
Eukaryota	unclassified (derived from Eukaryota)	*Naegleria*	0.004761294	0.004372464

Eukaryota	Ascomycota	*Nakaseomyces*	0.001465014	0.001239863
Eukaryota	Streptophyta	*Nandina*	0	0.000113804
Eukaryota	Arthropoda	*Nasonia*	0.002563774	0.001551326
Eukaryota	Ascomycota	*Naumovia*	0	5.98968E-06
Eukaryota	Ascomycota	*Nectria*	0.000732507	0.001138039
Eukaryota	Cnidaria	*Nematostella*	0.012818869	0.007828508
Eukaryota	Ascomycota	*Neolecta*	0	0.000724751
Eukaryota	Ascomycota	*Neosartorya*	0.006958815	0.005169091
Eukaryota	Chlorophyta	*Nephroselmis*	0.000732507	0.002371912
Eukaryota	Ascomycota	*Neurospora*	0.003296281	0.003617765
Eukaryota	Streptophyta	*Nicotiana*	0	2.99484E-05
Eukaryota	Microsporidia	*Nosema*	0.000366253	0.000275525
Eukaryota	unclassified (derived from Eukaryota)	*Ochromonas*	0	2.39587E-05
Eukaryota	Bacillariophyta	*Odontella*	0	0.000275525
Eukaryota	Chlorophyta	*Oedogonium*	0.00109876	0.002312015
Eukaryota	Streptophyta	*Oenothera*	0	2.99484E-05
Eukaryota	Chordata	*Oikopleura*	0	5.98968E-06
Eukaryota	Chlorophyta	*Oltmannsiellopsis*	0	0.001191946
Eukaryota	Streptophyta	*Oncidium*	0	1.19794E-05
Eukaryota	Annelida	*Orbinia*	0	5.98968E-06
Eukaryota	Chordata	*Ornithorhynchus*	0.001831267	0.000784648
Eukaryota	Chordata	*Oryctolagus*	0.000366253	0.000293494
Eukaryota	Streptophyta	*Oryza*	0.01611515	0.007804549
Eukaryota	Chlorophyta	*Ostreococcus*	0.06116432	0.021077673
Eukaryota	Chordata	*Pan*	0.002930027	0.001772944
Eukaryota	Chlorophyta	*Parachlorella*	0	0.001251842
Eukaryota	Ascomycota	*Paracoccidioides*	0.000732507	0.000407298
Eukaryota	unclassified (derived from Eukaryota)	*Paramecium*	0.00219752	0.002216181
Eukaryota	unclassified (derived from Eukaryota)	*Paulinella*	0.000366253	0.00055105
Eukaryota	Arthropoda	*Pediculus*	0.001465014	0.001018245
Eukaryota	Streptophyta	*Pelargonium*	0	5.98968E-06
Eukaryota	Ascomycota	*Penicillium*	0.004028788	0.001874769
Eukaryota	unclassified (derived from Eukaryota)	*Perkinsus*	0.005860055	0.002887024
Eukaryota	Bacillariophyta	*Phaeodactylum*	0.004028788	0.003809435
Eukaryota	Ascomycota	*Phaeosphaeria*	0.001831267	0.002018521
Eukaryota	Streptophyta	*Phalaenopsis*	0.000366253	0
Eukaryota	Streptophyta	*Phaseolus*	0	0.000239587
Eukaryota	Streptophyta	*Physcomitrella*	0.021608951	0.013812195
Eukaryota	unclassified (derived from Eukaryota)	*Phytophthora*	0.007325068	0.004977422
Eukaryota	Streptophyta	*Picea*	0	1.7969E-05
Eukaryota	Ascomycota	*Pichia*	0.001831267	0.00093439

Eukaryota	Streptophyta	*Pinus*	0	0.000946369
Eukaryota	Streptophyta	*Piper*	0	8.38555E-05
Eukaryota	unclassified (derived from Fungi)	*Piptocephalis*	0	0.001940655
Eukaryota	Apicomplexa	*Plasmodium*	0.032962807	0.077248865
Eukaryota	Streptophyta	*Platanus*	0	0.00019167
Eukaryota	Streptophyta	*Pleurozia*	0	1.19794E-05
Eukaryota	unclassified (derived from Eukaryota)	*Plocamiocolax*	0	1.19794E-05
Eukaryota	Ascomycota	*Podospora*	0.001465014	0.001443512
Eukaryota	Chordata	*Pongo*	0.001465014	0.001209915
Eukaryota	Streptophyta	*Populus*	0.009156335	0.006331089
Eukaryota	unclassified (derived from Eukaryota)	*Porphyra*	0.000732507	0.002276077
Eukaryota	Basidiomycota	*Postia*	0.000366253	0.000269535
Eukaryota	Chlorophyta	*Prototheca*	0	0.000125783
Eukaryota	Streptophyta	*Prunus*	0	7.78658E-05
Eukaryota	Chlorophyta	*Pseudendoclonium*	0.000366253	0.001910707
Eukaryota	Streptophyta	*Psilotum*	0	0.000251566
Eukaryota	Streptophyta	*Pteridium*	0	5.98968E-06
Eukaryota	Chlorophyta	*Pycnococcus*	0	0.000856524
Eukaryota	Phaeophyceae	*Pylaiella*	0.000366253	0.000281515
Eukaryota	Chlorophyta	*Pyramimonas*	0	0.000874493
Eukaryota	Ascomycota	*Pyrenophora*	0.001831267	0.000796627
Eukaryota	unclassified (derived from Eukaryota)	*Pythium*	0	1.7969E-05
Eukaryota	Chordata	*Rattus*	0.012818869	0.016405725
Eukaryota	unclassified (derived from Eukaryota)	*Reclinomonas*	0.000366253	0.000245577
Eukaryota	unclassified (derived from Fungi)	*Rhizopus*	0	1.19794E-05
Eukaryota	unclassified (derived from Eukaryota)	*Rhodomonas*	0	0.000485164
Eukaryota	Streptophyta	*Ricinus*	0.009522589	0.00946369
Eukaryota	Ascomycota	*Saccharomyces*	0.005127548	0.003581827
Eukaryota	Hemichordata	*Saccoglossus*	0.001465014	0.001084132
Eukaryota	unclassified (derived from Eukaryota)	*Saprolegnia*	0	2.39587E-05
Eukaryota	Chlorophyta	*Scenedesmus*	0.005860055	0.018094814
Eukaryota	Ascomycota	*Scheffersomyces*	0.003662534	0.002845097
Eukaryota	Platyhelminthes	*Schistosoma*	0.000732507	0.000886472
Eukaryota	Basidiomycota	*Schizophyllum*	0.00109876	0.001701068
Eukaryota	Ascomycota	*Schizosaccharomyces*	0.006958815	0.00872097
Eukaryota	Ascomycota	*Sclerotinia*	0.004395041	0.00222816
Eukaryota	Streptophyta	*Selaginella*	0.011720109	0.003983135
Eukaryota	unclassified (derived from Fungi)	*Smittium*	0	1.19794E-05
Eukaryota	Streptophyta	*Sorghum*	0.007325068	0.020376881

Eukaryota	Streptophyta	*Staurastrum*	0	0.000652875
Eukaryota	Chlorophyta	*Stigeoclonium*	0.000732507	0.002060449
Eukaryota	Echinodermata	*Strongylocentrotus*	0.004028788	0.002731293
Eukaryota	Chordata	*Sus*	0.000366253	0.00055704
Eukaryota	Bacillariophyta	*Synedra*	0	5.98968E-06
Eukaryota	Streptophyta	*Syntrichia*	0.000366253	8.98452E-05
Eukaryota	Chordata	*Taeniopygia*	0.00109876	0.000844544
Eukaryota	Ascomycota	*Talaromyces*	0.00219752	0.000850534
Eukaryota	unclassified (derived from Eukaryota)	*Tetrahymena*	0.002930027	0.001814872
Eukaryota	Bacillariophyta	*Thalassiosira*	0.002930027	0.003557868
Eukaryota	Apicomplexa	*Theileria*	0	0.000766679
Eukaryota	Arthropoda	*Thrips*	0	5.98968E-06
Eukaryota	Apicomplexa	*Toxoplasma*	0.00219752	0.002120346
Eukaryota	Arthropoda	*Tribolium*	0.002563774	0.001156008
Eukaryota	unclassified (derived from Eukaryota)	*Trichomonas*	0.004028788	0.001713048
Eukaryota	Ascomycota	*Trichophyton*	0	0.00017969
Eukaryota	Placozoa	*Trichoplax*	0.003662534	0.002725303
Eukaryota	Streptophyta	*Trifolium*	0	0.000329432
Eukaryota	Chordata	*Tropidophis*	0	5.98968E-06
Eukaryota	unclassified (derived from Eukaryota)	*Trypanosoma*	0.004395041	0.002994839
Eukaryota	Ascomycota	*Tuber*	0.001465014	0.000497143
Eukaryota	Ascomycota	*Uncinocarpus*	0.000732507	0.000335422
Eukaryota	Basidiomycota	*Ustilago*	0.008423828	0.008840763
Eukaryota	Porifera	*Vaceletia*	0	1.19794E-05
Eukaryota	Ascomycota	*Vanderwaltozyma*	0.000732507	0.000892462
Eukaryota	Xanthophyceae	*Vaucheria*	0	0.000173701
Eukaryota	Ascomycota	*Verticillium*	0.001465014	0.000682823
Eukaryota	Streptophyta	*Vitis*	0.006226308	0.004989401
Eukaryota	Chlorophyta	*Volvox*	0.167744062	0.04859425
Eukaryota	Streptophyta	*Welwitschia*	0	0.000107814
Eukaryota	Chordata	*Xenopus*	0.007691322	0.00870899
Eukaryota	Ascomycota	*Yarrowia*	0.004028788	0.004779762
Eukaryota	Streptophyta	*Zea*	0	8.98452E-05
Eukaryota	Streptophyta	*Zygnema*	0.000366253	0.000335422
Eukaryota	Ascomycota	*Zygosaccharomyces*	0.001465014	0.00148544
Eukaryota	Chromerida	Unclassified Chromerida	0	1.7969E-05
Eukaryota	Cnidaria	Unclassified *Isididae*	0	5.98968E-06
Eukaryota	unclassified (derived from Eukaryota)	Unclassified *Pelagophyceae*	0	2.39587E-05

Supplementary Table S5. Abundance profile of the Subsystem annotated protein features identified in the current metagenomic datasets (raw and assembled)**.**

Subsystem Hierarchy Level-I	Relative Abundance (%) in assembled dataset	Relative Abundance (%) in raw reads dataset
Amino Acids and Derivatives	9.552264752	10.50694002
Carbohydrates	10.23674232	10.68152952
Cell Division and Cell Cycle	1.10820178	1.170300902
Cell Wall and Capsule	2.912832325	2.418073607
Clustering-based subsystems	11.08745016	11.04144521
Cofactors, Vitamins, Prosthetic Groups, Pigments	7.427124868	6.394979791
DNA Metabolism	7.127258504	7.268978079
Dormancy and Sporulation	0.302039309	0.188037026
Fatty Acids, Lipids, and Isoprenoids	2.209884725	2.359425305
Iron acquisition and metabolism	0.486739605	0.37225148
Membrane Transport	3.194228659	2.918541148
Metabolism of Aromatic Compounds	0.739887659	0.465851185
Miscellaneous	7.553155659	6.753936031
Motility and Chemotaxis	1.54605013	0.984502594
Nitrogen Metabolism	1.068002303	1.097748185
Nucleosides and Nucleotides	4.302430439	4.405582478
Phages, Prophages, Transposable elements, Plasmids	1.198378983	0.691748423
Phosphorus Metabolism	1.325496246	1.383131341
Photosynthesis	0.428070099	0.363266152
Potassium metabolism	0.818113667	0.773149403
Protein Metabolism	9.648960789	11.77685623
RNA Metabolism	6.768722634	6.429154496
Regulation and Cell signaling	0.784433024	0.64858839
Respiration	3.228995774	4.335253249
Secondary Metabolism	0.253148054	0.263620387
Stress Response	1.98389848	1.871004194
Sulfur Metabolism	0.496517856	0.401689851
Virulence, Disease and Defense	2.210971198	2.034415329

Supplementary Table S6. Metagenomic protein features associated with acid stress, Temperature stress, desiccation, and periplasmic stress- response physiology in Sambhar salt lake metagenome.

Sr No	Type of Stress		Identified protein features	Phylogenetic afflation (% abundance)
1	Acid stress		Glutamate transporter , Glutamate decarboxylase, Arginine decarboxylase, glutamate/gamma-amino butyrate antiporters	Proteobacteria (74%), Bacteroidetes (3%), Cyanobacteria (3%), CandidatusCloacimonetes (3%), Firmicutes (2%), and Spirochaetes (1%)
2	Temperatures stress	Heat Stress	Chaperone protein (DnaJ), (DnaK)<br>DNA replication initiation control protein (YabA); Heat shock protein (GrpE), Heat-inducible transcription repressor (HrcA), Transcriptional repressor of DnaK operon (HspR), RNA polymerase sigma factor (RpoH), Radical SAM family enzyme (RdgB), Ribonuclease PH, Ribosomal RNA small subunit methyltransferase E, Ribosomal protein L11 methyltransferase<br>Signal peptidase-like protein, Translation elongation factor (LepA), tmRNA-binding protein (SmpB)	Proteobacteria (28%), Firmicutes (20%), Euryarchaeota (19%), FCB group (6%), Bacteroidetes (6%), Balneolaeota (0.5%), CandidatusCloacimonetes (0.1%), CandidatusNanohaloarchaeota (1.31%), Ignavibacteriae (0.2%), Nitrospirae (0.2%), Planctomycetes (0.18%), %), Verrucomicrobia (0.17%)
		Cold shock.	Cold shock proteins CspA, CspB, CspC, CspD, CspE, CspF, and CspG.	Firmicutes (56%), Proteobacteria (16%), Bacteroidetes (8.80%), Balneolaeota (1%), Chloroflexi (3%), Actinobacteria (1%) and others (0.18%).
3	Periplasmic and Desiccation stress		Chaperone protein (HtrA), Intramembrane protease (RasP/YluC), Outer membrane protein H precursor, Outer membrane stress sensor protease DegQ&DegS, Sigma factor RpoE negative regulatory protein RseA&RseB, Survival protein (SurA) O-antigen capsule important for environmental persistence	Euryarchaeota (16%), Proteobacteria (39%), Firmicutes (9%), Bacteroidetes (8%), Actinobacteria (2%), Acidobacteria (0.3%), Balneolaeota (0.5%), Spirochaetes (0.8%), Fusobacteriales (0.4%), Cyanobacteria (0.4%) and 9% are unclassified.

Supplementary Table S7. Metagenomic features associated with energy harvesting, energy fixation, and utilization in Sambhar salt lake metagenome.

Identified protein features		Phylogenetic afflation (% abundance)
**Energy harvesting (Photosynthesis)**	**Electron transport and photophosphorylation**	Viridiplantae (15.2%), Proteobacteria (7.2%), Unclassified Archaea (6.9%), Bacteroidetes/Chlorobi group (4.9%), Unclassified bacteria (2.1%), Unclassified Eukaryota (1.5%), Cyanobacteria/Melainabacteria group (1.3%), Actinobacteria (0.7%), Firmicutes (0.5%), Deinococcus-Thermus (0.5%), Balneolaeota (0.3%), Chloroflexi (0.2%), Terrabacteria group (0.2%), Bacteria candidate phyla (0.1%), FCB group Candidatus Kryptonia (0.1%), Planctomycetes (0.1%), Stramenopiles (0.1%), Opisthokonta (0.1%), FCB group Candidatus Aegiribacteria (0.05%), Rhodophyta (0.03%), DPANN group (0.03%), Spirochaetes (0.02%), Verrucomicrobia (0.01%), Nitrospinae/Tectomicrobia group (0.01%), Calditrichaeota (0.01%), FCB group (0.01%), TACK group (0.01%), and others
	**Light-harvesting complexes**	Euryarchaeota (23.1%), Firmicutes (22.3%), Proteobacteria (19.2%), Unclassified bacteria (11%), Bacteroidetes/Chlorobi group (5.7%), unclassified Archaea (4.1%), Actinobacteria (1.5%), Cyanobacteria/Melainabacteria group (0.7%), Terrabacteria group (0.7%), Spirochaetes (0.7%), Balneolaeota (0.6%), DPANN group (0.5%), Bacteria candidate phyla (0.5%), Chloroflexi (0.4%), Acidobacteria (0.4%), Verrucomicrobia (0.3%), Viridiplantae (0.2%), Planctomycetes (0.2%), Opisthokonta (0.2%), TACK group (0.2%), Fusobacteria (0.15%), Thermotogae (0.1%), Deinococcus-Thermus (0.1%), Gemmatimonadetes (0.1%), Tenericutes (0.1%), unclassified Eukaryota (0.1%), Elusimicrobia (0.06%), Nitrospirae (0.05%), Opisthokonta (0.04%), FCB group (0.04%), FCB group Candidatus Kryptonia (0.04%), Armatimonadetes (0.03%), PVC group Candidatus Omnitrophica (0.03%), Alveolata (0.02%), Haptophyceae (0.02%), candidate division Zixibacteria (0.01%), and others
**Energy Fixation**	**Carbon fixation**	Euryarchaeota (39.3%), Proteobacteria (18.3%), Firmicutes (12.2%), Unclassified bacteria (6.7%), Unclassified organisms (5.8%), Unclassified Archaea (5.47%), Bacteroidetes/Chlorobi group (4.2%), Cellular organisms (1.7%), Actinobacteria (1.5%), Cyanobacteria/Melainabacteria group (0.7%), Viridiplantae (0.4%), Terrabacteria group (0.4%), Archaea DPANN group (0.3%), Bacteria candidate phyla (0.3%), Spirochaetes (0.3%), Balneolaeota (0.3%), Acidobacteria (0.2%), Verrucomicrobia (0.2%), Chloroflexi (0.2%), Opisthokonta (0.2%), Archaea TACK group (0.1%), Gemmatimonadetes (0.1%), Planctomycetes (0.1%), Fusobacteria (0.07%), Deinococcus-Thermus (0.07%), Unclassified Eukaryota (0.06%), Alveolata (0.06%), Thermotogae (0.05%), Tenericutes (0.05%), Chlamydiae (0.03%), Aquificae (0.02%), Elusimicrobia (0.02%), FCB group (0.02%), Nitrospirae (0.02%), Synergistetes (0.02%), Armatimonadetes (0.02%), Euglenozoa (0.02%), Archaea Asgard group (0.01%), Candidate division Zixibacteria (0.01%), Candidatus Kryptonia (0.01%), and Candidatus Omnitrophica (0.01%), Haptophyceae (0.01%).
**Energy utilization**	**Fermentation**	Euryarchaeota (41.6%), Proteobacteria (19%), Firmicutes (11.9%), Unclassified Archaea (7.1%), Unclassified bacteria (5.03%), Bacteroidetes/Chlorobi group (3.72%), Actinobacteria (1.03%), Balneolaeota (0.43%), Terrabacteria group (0.36%),

	Cyanobacteria/Melainabacteria group (0.31%), Spirochaetes (0.3%), Chloroflexi (0.25%), Verrucomicrobia (0.17%), Acidobacteria (0.13%), DPANN group (0.12%), Bacteria candidate phyla (0.12%), Opisthokonta (0.1%), Planctomycetes (0.09%), Tenericutes (0.08%), Thermotogae (0.07%), Fusobacteria (0.06%), Chlamydiae (0.03%), TACK group (0.03%), PVC group Candidatus Omnitrophica (0.03%), Synergistetes (0.03%), Elusimicrobia (0.03%), Nitrospirae (0.03%), Viridiplantae (0.03%), FCB group (0.02%), Stramenopiles (0.02%), Rhodophyta (0.02%), Aquificae (0.01%), Deinococcus-Thermus (0.01%), Armatimonadetes (0.01%), Deferribacteres (0.01%), Caldiserica (0.01%), Euglenozoa (0.01%), and others

Supplementary Table S8. Functional metabolic pathways in the salt lake microbiome. Metabolic pathways within the Sambhar lake microbiome explored after mapping of the metabolite features captured through positive & negative ESI mode of the LC-MS analysis.

**Metabolite features captured through Positive ESI mode**		**Metabolite features captured through Negative ESI mode**	
**Pathway**	**Overlapping features**	**Pathway**	**Overlapping features**
Protein ubiquitylation	43	Protein ubiquitylation	43
Phospholipases	43	phospholipases	43
tRNA charging	39	tRNA charging	39
mucin core 1 and core 2 O-glycosylation	29	3-phosphoinositide biosynthesis	29
3-phosphoinositide biosynthesis	29	mucin core 1 and core 2 O-glycosylation	29
stearate biosynthesis	29	stearate biosynthesis	29
Glutathione-mediated detoxification	28	glutathione-mediated detoxification	28
triacylglycerol biosynthesis	27	adenosine ribonucleotides de novo biosynthesis	27
D-myo-inositol (1,4,5)-trisphosphate biosynthesis	27	D-myo-inositol (1,4,5)-trisphosphate biosynthesis	27
adenosine ribonucleotides de novo biosynthesis	27	triacylglycerol biosynthesis	27
Fatty acid α-oxidation III	25	fatty acid α-oxidation	25
Fatty acid α-oxidation	25	fatty acid α-oxidation III	25
Fatty acid β-oxidation	24	glycolysis	24
Gluconeogenesis	24	gluconeogenesis	24
glycolysis	24	fatty acid β-oxidation	24
Fatty acid β-oxidation (peroxisome)	23	fatty acid β-oxidation (peroxisome)	23
CDP-diacylglycerol biosynthesis	22	CDP-diacylglycerol biosynthesis	22
heparan sulfate biosynthesis (late stages)	22	heparan sulfate biosynthesis (late stages)	22
D-myo-inositol-5-phosphate metabolism	20	D-myo-inositol-5-phosphate metabolism	20
3-phosphoinositide degradation	20	3-phosphoinositide degradation	20
Retinol biosynthesis	18	retinol biosynthesis	18
ethanol degradation II	17	pyrimidine deoxyribonucleotides biosynthesis from CTP	17
Pyrimidine deoxyribonucleotides biosynthesis from CTP	17	ethanol degradation II	17
Triacylglycerol degradation	16	triacylglycerol degradation	16
The visual cycle I (vertebrates)	16	the visual cycle I (vertebrates)	16
Fatty acid activation	15	nicotine degradation IV	15
D-myo-inositol (1,3,4)-trisphosphate biosynthesis	15	D-myo-inositol (1,3,4)-trisphosphate biosynthesis	15
1D-myo-inositol hexakisphosphate biosynthesis II (mammalian)	15	pyrimidine deoxyribonucleotides de novo biosynthesis	15
Pyrimidine deoxyribonucleotides de novo biosynthesis	15	fatty acid activation	15
nicotine degradation IV	15	1D-myo-inositol hexakisphosphate biosynthesis II (mammalian)	15
UDP-N-acetyl-D-galactosamine biosynthesis II	14	UDP-N-acetyl-D-galactosamine biosynthesis II	14
γ-linolenate biosynthesis	14	γ-linolenate biosynthesis	14
terminal O-glycans residues modification	13	bile acid biosynthesis, neutral pathway	13
Icosapentaenoate biosynthesis II (metazoa)	13	terminal O-glycans residues modification	13
D-myo-inositol (1,4,5)-trisphosphate degradation	13	D-myo-inositol (1,4,5)-trisphosphate degradation	13
Palmitate biosynthesis	13	icosapentaenoate biosynthesis II (metazoa)	13

Bile acid biosynthesis, neutral pathway	13	palmitate biosynthesis	13
UTP and CTP dephosphorylation I	12	androgen biosynthesis	12
oxidative ethanol degradation III	12	UTP and CTP dephosphorylation I	12
folate transformations I	12	TCA cycle	12
TCA cycle	12	folate transformations I	12
androgen biosynthesis	12	oxidative ethanol degradation III	12
ethanol degradation IV	11	sphingosine and sphingosine-1-phosphate metabolism	11
UTP and CTP de novo biosynthesis	11	guanosine ribonucleotides de novo biosynthesis	11
guanosine ribonucleotides de novo biosynthesis	11	UTP and CTP de novo biosynthesis	11
purine deoxyribonucleosides salvage	11	purine deoxyribonucleosides salvage	11
Sphingosine and sphingosine-1-phosphate metabolism	11	ethanol degradation IV	11
Noradrenaline and adrenaline degradation	11	noradrenaline and adrenaline degradation	11
docosahexaenoate biosynthesis III (mammals)	10	morphine biosynthesis	10
Mevalonate pathway	10	putrescine degradation III	10
Pyrimidine deoxyribonucleotide phosphorylation	10	docosahexaenoate biosynthesis III (mammals)	10
Sphingomyelin metabolism/ceramide salvage	10	pyrimidine deoxyribonucleotide phosphorylation	10
putrescine degradation III	10	sphingomyelin metabolism/ceramide salvage	10
melatonin degradation I	10	melatonin degradation I	10
Valine degradation	10	mevalonate pathway	10
Methylglyoxal degradation VI	9	valine degradation	10
Acetone degradation I (to methylglyoxal)	9	isoleucine degradation	9
CMP phosphorylation	9	glycogenolysis	9
guanosine deoxyribonucleotides de novo biosynthesis	9	guanosine deoxyribonucleotides de novo biosynthesis	9
adenosine deoxyribonucleotides de novo biosynthesis	9	thyroid hormone metabolism II (via conjugation and/or degradation)	9
Oleate biosynthesis	9	adenosine deoxyribonucleotides de novo biosynthesis	9
C20 prostanoid biosynthesis	9	urate biosynthesis/inosine 5-phosphate degradation	9
Serotonin degradation	9	adenosine nucleotides degradation	9
isoleucine degradation	9	C20 prostanoid biosynthesis	9
glycogenolysis	9	methylglyoxal degradation VI	9
thyroid hormone metabolism II (via conjugation and/or degradation)	9	chondroitin sulfate degradation (metazoa)	9
urate biosynthesis/inosine 5-phosphate degradation	9	acetone degradation I (to methylglyoxal)	9
Chondroitin sulfate degradation (metazoa)	9	oleate biosynthesis	9
adenosine nucleotides degradation	9	serotonin degradation	9
chondroitin sulfate biosynthesis (late stages)	8	CMP phosphorylation	9
Dolichyl-diphosphooligosaccharide biosynthesis	8	glycogen biosynthesis	8
glycogen biosynthesis	8	nicotine degradation III	8
4-hydroxy-2-nonenal detoxification	8	phytol degradation	8
Retinoate biosynthesis I	8	chondroitin sulfate biosynthesis (late stages)	8
Leucine degradation	8	leucine degradation	8
phytol degradation	8	sucrose degradation	8
Dermatan sulfate degradation (metazoa)	8	dermatan sulfate degradation (metazoa)	8
nicotine degradation III	8	retinoate biosynthesis I	8

Sucrose degradation	8	dolichyl-diphosphooligosaccharide biosynthesis	8
GDP-glucose biosynthesis II	7	4-hydroxy-2-nonenal detoxification	8
UDP-N-acetyl-D-glucosamine biosynthesis II	7	leukotriene biosynthesis	7
Selenocysteine biosynthesis	7	tryptophan degradation to 2-amino-3-carboxymuconate semialdehyde	7
glycoaminoglycan-protein linkage region biosynthesis	7	guanosine nucleotides degradation	7
ceramide de novo biosynthesis	7	ceramide de novo biosynthesis	7
Inositol pyrophosphates biosynthesis	7	glycoaminoglycan-protein linkage region biosynthesis	7
tryptophan degradation to 2-amino-3-carboxymuconate semialdehyde	7	inositol pyrophosphates biosynthesis	7
guanosine nucleotides degradation	7	UDP-N-acetyl-D-glucosamine biosynthesis II	7
leukotriene biosynthesis	7	selenocysteine biosynthesis	7
Glutaryl-CoA degradation	6	GDP-glucose biosynthesis II	7
Ketogenesis	6	D-galactose degradation V (Leloir pathway)	6
GDP-mannose biosynthesis	6	dopamine degradation	6
glutathione redox reactions I	6	GDP-mannose biosynthesis	6
Folate polyglutamylation	6	ubiquinol-10 biosynthesis	6
Phosphatidylethanolamine biosynthesis II	6	2-deoxy-α-D-ribose 1-phosphate degradation	6
phosphatidylcholine biosynthesis	6	trehalose degradation	6
dopamine degradation	6	glutathione redox reactions I	6
2-deoxy-α-D-ribose 1-phosphate degradation	6	ketogenesis	6
Spermine and spermidine degradation I	6	folate polyglutamylation	6
trehalose degradation	6	zymosterol biosynthesis	6
Allopregnanolone biosynthesis	6	allopregnanolone biosynthesis	6
coenzyme A biosynthesis	6	coenzyme A biosynthesis	6
lysine degradation II (pipecolate pathway)	6	lysine degradation II (pipecolate pathway)	6
D-galactose degradation V (Leloir pathway)	6	phosphatidylcholine biosynthesis	6
ubiquinol-10 biosynthesis	6	phosphatidylethanolamine biosynthesis II	6
Zymosterol biosynthesis	6	spermine and spermidine degradation I	6
Morphine biosynthesis	6	glutaryl-CoA degradation	6
tRNA splicing	5	estradiol biosynthesis I	5
tryptophan degradation via tryptamine	5	fructose 2,6-bisphosphate synthesis	5
Bupropion degradation	5	tyrosine degradation	5
NAD biosynthesis from 2-amino-3-carboxymuconate semialdehyde	5	tryptophan degradation via tryptamine	5
Glycerol degradation	5	protein citrullination	5
Mitochondrial L-carnitine shuttle	5	UTP and CTP dephosphorylation II	5
protein citrullination	5	tetrapyrrole biosynthesis	5
UTP and CTP dephosphorylation II	5	thioredoxin pathway	5
thioredoxin pathway	5	serotonin and melatonin biosynthesis	5
Pyridoxal 5-phosphate salvage	5	creatine-phosphate biosynthesis	5
Pyruvate decarboxylation to acetyl CoA	5	citrulline-nitric oxide cycle	5
histidine degradation	5	urea cycle	5
fructose 2,6-bisphosphate synthesis	5	tRNA splicing	5
Thyronamine and iodothyronamine metabolism	5	histidine degradation	5
Pyrimidine deoxyribonucleosides salvage	5	NAD biosynthesis from 2-amino-3-carboxymuconate semialdehyde	5

Citrulline-nitric oxide cycle	5	glycine betaine degradation	5
tetrapyrrole biosynthesis	5	glycerol degradation	5
Creatine-phosphate biosynthesis	5	mitochondrial L-carnitine shuttle	5
Glycine betaine degradation	5	pyridoxal 5-phosphate salvage	5
urea cycle	5	pyruvate decarboxylation to acetyl CoA	5
serotonin and melatonin biosynthesis	5	thyronamine and iodothyronamine metabolism	5
tyrosine degradation	5	bupropion degradation	5
estradiol biosynthesis I	5	pyrimidine deoxyribonucleosides salvage	5
Acyl-CoA hydrolysis	4	S-methyl-5-thio-α-D-ribose 1-phosphate degradation	4
mRNA capping	4	phenylethylamine degradation I	4
Pyrimidine ribonucleosides salvage I	4	S-methyl-5-thio-α-D-ribose 1-phosphate degradation	4
2-oxoisovalerate decarboxylation to isobutanoyl-CoA	4	CMP-N-acetylneuraminate biosynthesis I (eukaryotes)	4
dermatan sulfate biosynthesis (late stages)	4	thyroid hormone metabolism I (via deiodination)	4
Eumelanin biosynthesis	4	dermatan sulfate biosynthesis (late stages)	4
Molybdenum cofactor biosynthesis	4	catecholamine biosynthesis	4
fatty acid biosynthesis initiation	4	ketolysis	4
2-oxoglutarate decarboxylation to succinyl-CoA	4	pentose phosphate pathway (non-oxidative branch)	4
Heme degradation	4	malate-aspartate shuttle	4
NAD salvage	4	asparagine degradation	4
4-hydroxyproline degradation	4	acyl-CoA hydrolysis	4
Phenylalanine degradation/tyrosine biosynthesis	4	mRNA capping	4
Retinoate biosynthesis II	4	2-oxoglutarate decarboxylation to succinyl-CoA	4
Ketolysis	4	2-oxoisovalerate decarboxylation to isobutanoyl-CoA	4
Malate-aspartate shuttle	4	fatty acid biosynthesis initiation	4
Asparagine degradation	4	lysine degradation I (saccharopine pathway)	4
heme biosynthesis from uroporphyrinogen-III I	4	heme biosynthesis from uroporphyrinogen-III I	4
catecholamine biosynthesis	4	NAD salvage	4
Trans, trans-farnesyl diphosphate biosynthesis	4	4-hydroxyproline degradation	4
thyroid hormone metabolism I (via deiodination)	4	molybdenum cofactor biosynthesis	4
phenylethylamine degradation I	4	phenylalanine degradation/tyrosine biosynthesis	4
Serine biosynthesis (phosphorylated route)	4	serine biosynthesis (phosphorylated route)	4
Ornithine de novo biosynthesis	4	retinoate biosynthesis II	4
Pentose phosphate pathway (non-oxidative branch)	4	eumelanin biosynthesis	4
S-methyl-5-thio-α-D-ribose 1-phosphate degradation	4	ornithine de novo biosynthesis	4
CMP-N-acetylneuraminate biosynthesis I (eukaryotes)	4	heme degradation	4
Pyrimidine deoxyribonucleosides degradation	4	trans, trans-farnesyl diphosphate biosynthesis	4
lysine degradation I (saccharopine pathway)	4	pyrimidine deoxyribonucleosides degradation	4
S-methyl-5-thio-α-D-ribose 1-phosphate degradation	4	pyrimidine ribonucleosides salvage I	4
Fatty acid β-oxidation (unsaturated, odd number)	3	lipoxin biosynthesis	3
Lactate fermentation (reoxidation of cytosolic NADH)	3	glucocorticoid biosynthesis	3
Taurine biosynthesis	3	mineralocorticoid biosynthesis	3

Uracil degradation	3	L-carnitine biosynthesis	3
PRPP biosynthesis	3	inosine-5-phosphate biosynthesis	3
N-acetylglucosamine degradation I	3	glycine cleavage	3
Methylglyoxal degradation I	3	UMP biosynthesis	3
cardiolipin biosynthesis	3	threonine degradation	3
dTMP de novo biosynthesis (mitochondrial)	3	dTMP de novo biosynthesis (mitochondrial)	3
glycine cleavage	3	adenine and adenosine salvage III	3
UMP biosynthesis	3	5-aminoimidazole ribonucleotide biosynthesis	3
NADH repair	3	tetrahydrobiopterin de novo biosynthesis	3
Proline biosynthesis	3	purine ribonucleosides degradation to ribose-1-phosphate	3
Acetate conversion to acetyl-CoA	3	pentose phosphate pathway (oxidative branch)	3
Propionyl-CoA degradation	3	propionyl-CoA degradation	3
Methylglyoxal degradation III	3	methylglyoxal degradation III	3
D-myo-inositol (1,4,5,6)-tetrakisphosphate biosynthesis	3	methylglyoxal degradation I	3
Biotin-carboxyl carrier protein assembly	3	methionine salvage	3
1D-myo-inositol hexakisphosphate biosynthesis V (from Ins(1,3,4)P3)	3	lactose degradation III	3
threonine degradation	3	D-myo-inositol (1,4,5,6)-tetrakisphosphate biosynthesis	3
Arsenate detoxification I (glutaredoxin)	3	dolichol and dolichyl phosphate biosynthesis	3
7-(3-amino-3-carboxypropyl)-wyosine biosynthesis	3	biotin-carboxyl carrier protein assembly	3
tetrahydrobiopterin de novo biosynthesis	3	7-(3-amino-3-carboxypropyl)-wyosine biosynthesis	3
Pyrimidine ribonucleosides degradation	3	cardiolipin biosynthesis	3
Myo-inositol de novo biosynthesis	3	pyrimidine ribonucleosides degradation	3
Pentose phosphate pathway (oxidative branch)	3	myo-inositol de novo biosynthesis	3
5-aminoimidazole ribonucleotide biosynthesis	3	lactate fermentation (reoxidation of cytosolic NADH)	3
Thymine degradation	3	sorbitol degradation I	3
Sorbitol degradation I	3	NADH repair	3
Methionine salvage	3	taurine biosynthesis	3
Lactose degradation III	3	proline biosynthesis	3
Dolichol and dolichyl phosphate biosynthesis	3	uracil degradation	3
Methionine degradation	3	PRPP biosynthesis	3
lipoxin biosynthesis	3	1D-myo-inositol hexakisphosphate biosynthesis V (from Ins(1,3,4)P3)	3
glucocorticoid biosynthesis	3	N-acetylglucosamine degradation I	3
inosine-5-phosphate biosynthesis	3	acetate conversion to acetyl-CoA	3
adenine and adenosine salvage III	3	thymine degradation	3
purine ribonucleosides degradation to ribose-1-phosphate	3	methionine degradation	3
mineralocorticoid biosynthesis	3	fatty acid β-oxidation (unsaturated, odd number)	3
L-carnitine biosynthesis	3	arsenate detoxification I (glutaredoxin)	3
BMP Signalling Pathway	2	aspirin triggered resolvin E biosynthesis	2
arachidonate biosynthesis IV (8-detaturase)	2	glutamate biosynthesis/degradation	2
D-glucuronate degradation	2	glutamine degradation/glutamate biosynthesis	2
NAD phosphorylation and dephosphorylation	2	aspirin-triggered lipoxin biosynthesis	2
MAP kinase cascade	2	aspirin triggered resolvin D biosynthesis	2

Proline degradation	2	resolvin D biosynthesis	2
2-amino-3-carboxymuconate semialdehyde degradation to Glutaryl-CoA	2	Rapoport-Luebering glycolytic shunt	2
Glycerol-3-phosphate shuttle	2	arachidonate biosynthesis IV (8-detaturase)	2
Hypusine biosynthesis	2	glycine/serine biosynthesis	2
L-glutamine tRNA biosynthesis	2	glycine biosynthesis	2
Tetrahydrofolate salvage from 5,10-methenyltetrahydrofolate	2	putrescine biosynthesis II	2
formaldehyde oxidation	2	ascorbate recycling (cytosolic)	2
ascorbate recycling (cytosolic)	2	spermidine biosynthesis	2
GDP-L-fucose biosynthesis I (from GDP-D-mannose)	2	histamine degradation	2
GDP-L-fucose biosynthesis II (from L-fucose)	2	4-aminobutyrate degradation	2
cysteine biosynthesis/homocysteine degradation (trans-sulfuration)	2	GDP-L-fucose biosynthesis I (from GDP-D-mannose)	2
chondroitin biosynthesis	2	GDP-L-fucose biosynthesis II (from L-fucose)	2
glutamine degradation/glutamate biosynthesis	2	protein O-[N-acetyl]-glucosylation	2
glycine/serine biosynthesis	2	cysteine biosynthesis/homocysteine degradation (trans-sulfuration)	2
glycine biosynthesis	2	adenine and adenosine salvage I	2
Hydrogen sulfide biosynthesis (trans-sulfuration)	2	guanine and guanosine salvage	2
glutathione biosynthesis	2	alanine biosynthesis/degradation	2
Rapoport-Luebering glycolytic shunt	2	lipoate biosynthesis and incorporation	2
Diphthamide biosynthesis	2	glutathione biosynthesis	2
fatty acid elongation -- saturated	2	purine deoxyribonucleosides degradation	2
thyroid hormone biosynthesis	2	chondroitin biosynthesis	2
D-myo-inositol (3,4,5,6)-tetrakisphosphate biosynthesis	2	vitamin D3 biosynthesis	2
Flavin biosynthesis	2	thyroid hormone biosynthesis	2
β-alanine degradation	2	progesterone biosynthesis	2
Aspartate biosynthesis	2	estradiol biosynthesis II	2
L-cysteine degradation II	2	glycerol-3-phosphate shuttle	2
L-cysteine degradation I	2	L-cysteine degradation II	2
Spermine biosynthesis	2	L-cysteine degradation I	2
Asparagine biosynthesis	2	spermine biosynthesis	2
Choline degradation	2	creatine biosynthesis	2
spermidine biosynthesis	2	asparagine biosynthesis	2
histamine degradation	2	diphthamide biosynthesis	2
protein O-[N-acetyl]-glucosylation	2	hypusine biosynthesis	2
alanine biosynthesis/degradation	2	choline degradation	2
glutamate biosynthesis/degradation	2	L-glutamine tRNA biosynthesis	2
aspirin-triggered lipoxin biosynthesis	2	tetrahydrofolate salvage from 5,10-methenyltetrahydrofolate	2
S-adenosyl-L-methionine biosynthesis	2	D-myo-inositol (3,4,5,6)-tetrakisphosphate biosynthesis	2
Sulfate activation for sulfonation	2	epoxysqualene biosynthesis	2
L-serine degradation	2	fatty acid elongation -- saturated	2
L-dopa degradation	2	formaldehyde oxidation	2
adenine and adenosine salvage I	2	hydrogen sulfide biosynthesis (trans-sulfuration)	2
Creatine biosynthesis	2	flavin biosynthesis	2

guanine and guanosine salvage	2	L-serine degradation	2
lipoate biosynthesis and incorporation	2	sulfate activation for sulfonation	2
aspirin triggered resolvin E biosynthesis	2	MAP kinase cascade	2
putrescine biosynthesis II	2	BMP Signalling Pathway	2
Epoxysqualene biosynthesis	2	S-adenosyl-L-methionine biosynthesis	2
purine deoxyribonucleosides degradation	2	anandamide degradation	2
vitamin D3 biosynthesis	2	proline degradation	2
progesterone biosynthesis	2	L-dopa degradation	2
Anandamide degradation	2	β-alanine degradation	2
4-aminobutyrate degradation	2	aspartate biosynthesis	2
estradiol biosynthesis II	2	2-amino-3-carboxymuconate semialdehyde degradation to glutaryl-CoA	2
aspirin triggered resolvin D biosynthesis	2	D-glucuronate degradation	2
resolvin D biosynthesis	2	NAD phosphorylation and dephosphorylation	2
Acyl carrier protein metabolism	1	α-tocopherol degradation	1
S-methyl-5 -thioadenosine degradation	1	pregnenolone biosynthesis	1
Oxidized GTP and dGTP detoxification	1	carnosine biosynthesis	1
UDP-N-acetyl-D-galactosamine biosynthesis I	1	homocarnosine biosynthesis	1
Thio-molybdenum cofactor biosynthesis	1	putrescine biosynthesis I	1
[2Fe-2S] Iron-sulfur cluster biosynthesis	1	phosphatidylserine biosynthesis I	1
L-dopachrome biosynthesis	1	phosphatidylserine biosynthesis II	1
Histamine biosynthesis	1	geranylgeranyldiphosphate biosynthesis	1
Glutamate removal from folates	1	4-hydroxybenzoate biosynthesis	1
glutamine biosynthesis	1	adenine and adenosine salvage II	1
melatonin degradation II	1	thiosulfate disproportionation III (rhodanese)	1
thiosulfate disproportionation III (rhodanese)	1	D-mannose degradation	1
D-mannose degradation	1	lipoate salvage	1
thiamin salvage III	1	thiamin salvage III	1
glutathione redox reactions II	1	glutathione redox reactions II	1
carnosine biosynthesis	1	histamine biosynthesis	1
lipoate salvage	1	glutamate removal from folates	1
melatonin degradation III	1	glutamine biosynthesis	1
Acetyl-CoA biosynthesis from citrate	1	lanosterol biosynthesis	1
Sulfite oxidation	1	melatonin degradation II	1
geranylgeranyldiphosphate biosynthesis	1	melatonin degradation III	1
adenine and adenosine salvage II	1	UDP-N-acetyl-D-galactosamine biosynthesis I	1
homocarnosine biosynthesis	1	(S)-reticuline biosynthesis	1
putrescine biosynthesis I	1	acetyl-CoA biosynthesis from citrate	1
phosphatidylserine biosynthesis II	1	sulfite oxidation	1
phosphatidylserine biosynthesis I	1	thio-molybdenum cofactor biosynthesis	1
Lanosterol biosynthesis	1	acyl carrier protein metabolism	1
4-hydroxybenzoate biosynthesis	1	[2Fe-2S] iron-sulfur cluster biosynthesis	1
α-tocopherol degradation	1	S-methyl-5-thioadenosine degradation	1
(S)-reticuline biosynthesis	1	L-dopachrome biosynthesis	1
pregnenolone biosynthesis	1	oxidized GTP and dGTP detoxification	1

Supplementary Table S9. Metabolic pathways predominantly represented in the captured metabolomics dataset. Metabolic pathways within the Sambhar lake microbiome explored after mapping of the metabolite features captured through positive and negative ESI mode of the LC-MS analysis.

Pathway	Total Pathway	Pathways represented by captured Metabolite profile
Purine metabolism	47	25
Cysteine and methionine metabolism	36	23
Pyrimidine metabolism	29	14
Galactose metabolism	26	9
Amino sugar and nucleotide sugar metabolism	25	9
Pentose phosphate pathway	24	15
Folate biosynthesis	24	12
Methane metabolism	23	12
Phenylalanine, tyrosine and tryptophan biosynthesis	22	17
Glycine, serine and threonine metabolism	22	14
Valine, leucine and isoleucine biosynthesis	22	19
Fructose and mannose metabolism	21	12
Pentose and glucuronate interconversions	20	13
Aminoacyl-tRNA biosynthesis	20	14
Glycolysis / Gluconeogenesis	19	10
Pantothenate and CoA biosynthesis	19	9
Thiamine metabolism	18	9
Starch and sucrose metabolism	15	2
Arginine biosynthesis	15	8
Lysine biosynthesis	14	9
Histidine metabolism	14	13
Alanine, aspartate and glutamate metabolism	13	6
Inositol phosphate metabolism	13	9
Glyoxylate and dicarboxylate metabolism	13	4
Nicotinate and nicotinamide metabolism	13	7
Cyanoamino acid metabolism	11	8
Pyruvate metabolism	10	4
One carbon pool by folate	9	3
Streptomycin biosynthesis	7	3
Monobactam biosynthesis	6	4
C5-Branched dibasic acid metabolism	6	4
Glycerolipid metabolism	5	3
Lipoic acid metabolism	4	3
Ubiquinone and other terpenoid-quinone biosynthesis	3	1
Biotin metabolism	3	3

Supplementary Table S10. Bonferroni-corrected p-values obtained after performing PERMANOVA test analysis to assess variations among various metagenomes (Freshwater ecosystem (n=131), alkaline ecosystem (n=14), and saline ecosystem (n=67)) based on the phylogenetic affiliated rRNA features at the Phylum level of the taxonomic hierarchy.

Bonferroni-corrected p-values	Alkaline	Freshwater	Saline water
**Alkaline**		0.0084	0.0024
**Freshwater**	0.0084		0.0003
**Saline water**	0.0024	0.0003	

Supplementary Table S11. Bonferroni-corrected p-values obtained after performing PERMANOVA test analysis to assess variations among various metagenomes (Freshwater ecosystem (n=131), alkaline ecosystem (n=14), and saline ecosystem (n=67)) based on the phylogenetic affiliated rRNA features at the class level of the taxonomic hierarchy.

Bonferroni-corrected p-values	Alkaline	Freshwater	Saline water
Alkaline		0.0015	0.0024
Freshwater biome	0.0015		0.0003
Saline water	0.0024	0.0003	

Supplementary Table S12. Bonferroni-corrected p-values obtained after performing PERMANOVA test analysis to assess variations among various metagenomes (Freshwater ecosystem (n=131), alkaline ecosystem (n=14), and saline ecosystem (n=67)) based on the phylogenetic affiliated protein features at the class level (A) and phylum level (b) of the taxonomic hierarchy.

A. Phylogenetic affiliated protein features at the class level of the taxonomic hierarchy.

Bonferroni-corrected p-values	Alkaline	Freshwater	Saline water
Alkaline		0.0003	0.0003
Freshwater	0.0003		0.0003
Saline water	0.0003	0.0003	

B. Phylogenetic affiliated protein features at the Phylum level of the taxonomic hierarchy.

Bonferroni-corrected p-values	Alkaline	Freshwater	Saline water
Alkaline		0.009	0.0003
Freshwater	0.009		0.258
Saline water	0.0003	0.258	

Supplementary Table S13. Bonferroni-corrected p-values obtained after performing PERMANOVA test analysis to assess variations among various metagenomes (Freshwater ecosystem (n=131), alkaline ecosystem (n=14), and saline ecosystem (n=67)) based on the Subsystem annotated protein features at hierarchy level 1.

Bonferroni-corrected p-values	Alkaline	Freshwater	Saline water
Alkaline		0.3666	0.4869
Freshwater	0.3666		0.0006
Saline water	0.4869	0.0006	

Supplementary Table S14. Bonferroni-corrected p-values obtained after performing PERMANOVA test analysis to assess variations among various metagenomes (Freshwater ecosystem (n=131), alkaline ecosystem (n=14), and saline ecosystem (n=67)) based on the Subsystem annotated protein features at hierarchy level 2.

Bonferroni-corrected p-values	Alkaline environment	Freshwater	Saline water
Alkaline environment		1	0.2928
Freshwater	1		0.048
Saline water	0.2928	0.048	

Supplementary Table S15. Kruskal-Wallis test for equal medians was performed with Mann-Whitney pair-wise comparisons test to assess variations among metagenome (Freshwater ecosystem (n=131), alkaline ecosystem (n=14), and saline ecosystem (n=67)) based on the subsystem annotated protein features associated with Dormancy and Sporulation (A), Motility and Chemotaxis (B), Metabolism of aromatic compounds (C), and Photosynthesis (D).

(A) Dormancy and Sporulation

**Bonferroni-corrected p-values**	**Alkaline environment**	**Freshwater**	**Saline water**
Alkaline environment		0.000001622	0.0000005487
Freshwater	0.000001622		0.000000000003728
Saline water	0.0000005487	0.000000000003728	

(B) Motility and Chemotaxis

**Bonferroni-corrected p-values**	**Alkaline environment**	**Freshwater**	**Saline water**
Alkaline environment		0.02049	0.000003023
Freshwater	0.02049		0.000000000005139
Saline water	0.000003023	0.000000000005139	

(C) **Metabolism of Aromatic compounds**

**Bonferroni-corrected p-values**	**Alkaline environment**	**Freshwater**	**Saline water**
Alkaline environment		0.000004484	0.3093
Freshwater	0.000004484		0.0000000000001856
Saline water	0.3093	0.0000000000001856	

(D) **Photosynthesis**

**Bonferroni-corrected p-values**	**Alkaline environment**	**Freshwater**	**Saline water**
Alkaline environment		0.1102	0.001321
Freshwater	0.1102		0.00000007149
Saline water	0.001321	0.00000007149	

Supplementary Figures (C)

Supplementary Figure S1: Rarefaction curve analysis based on the identified rRNA features (A) and protein features in the present metagenomic dataset. Statistical values obtained after processing of metagenomic dataset for identification of the rRNA and proteins features were plotted with Origin Pro8.

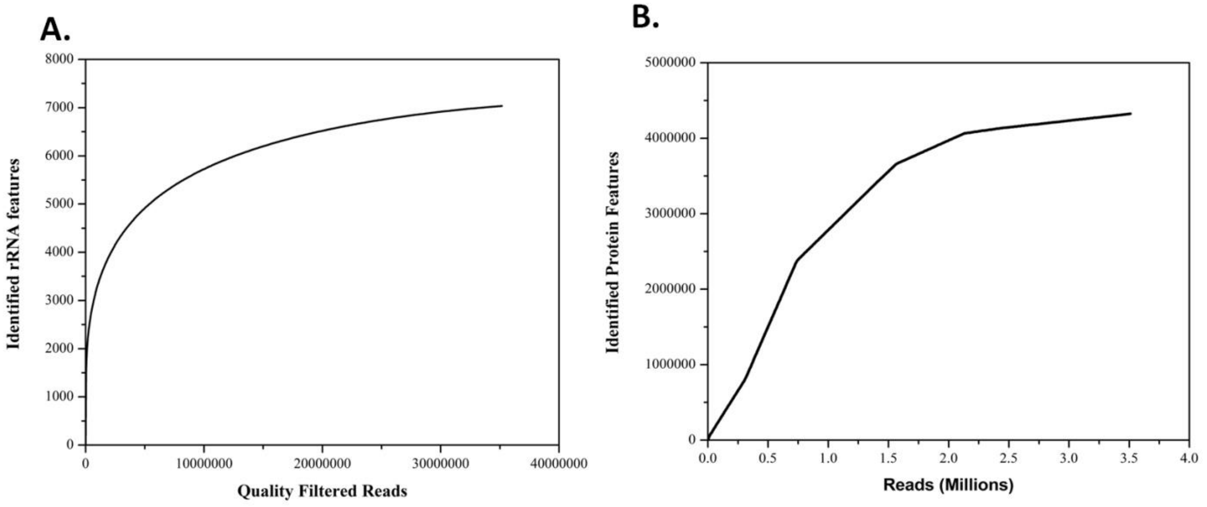

Supplementary Figure S2. The abundance of various osmotic stress response protein features identified within Salt Lake Metagenome. Here a relative abundance of protein features associated with A1.1: Betaine biosynthesis from glycine, A1.2: Choline and Betaine Uptake and Betaine Biosynthesis: A1.3: Ectoine & Hydroxyectoine Biosynthesis, A.1.4: Biosynthesis of osmoregulator periplasmic glucans (OPGs), A.1.5: ABC Transporters, and A.1.6: Osmotic stress cluster proteins are shown.

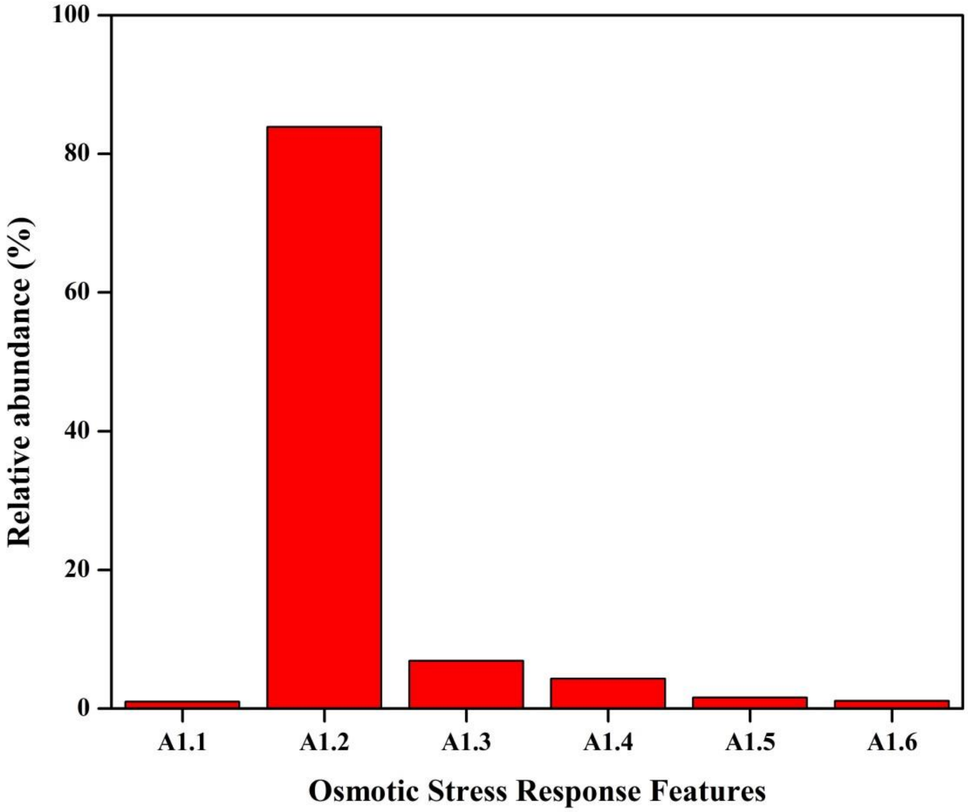

Supplementary Figure S3. Ternary plot indicating the distribution of phylogenetic affiliated protein features at phylum level of the taxonomic hierarchy among various ecosystem datasets.

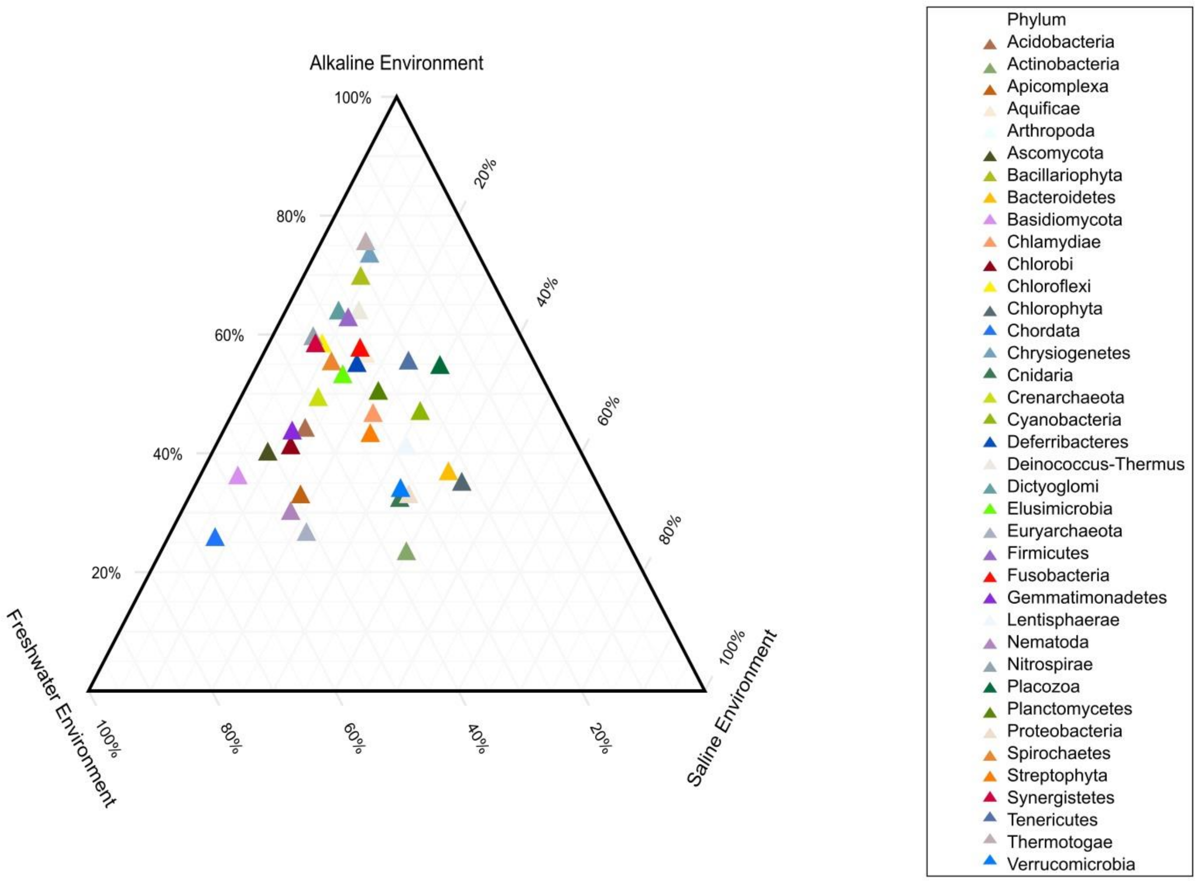

Supplementary Figure S4. Ternary plot indicating the abundance of phylogenetic affiliated protein features at class level of the taxonomic hierarchy.

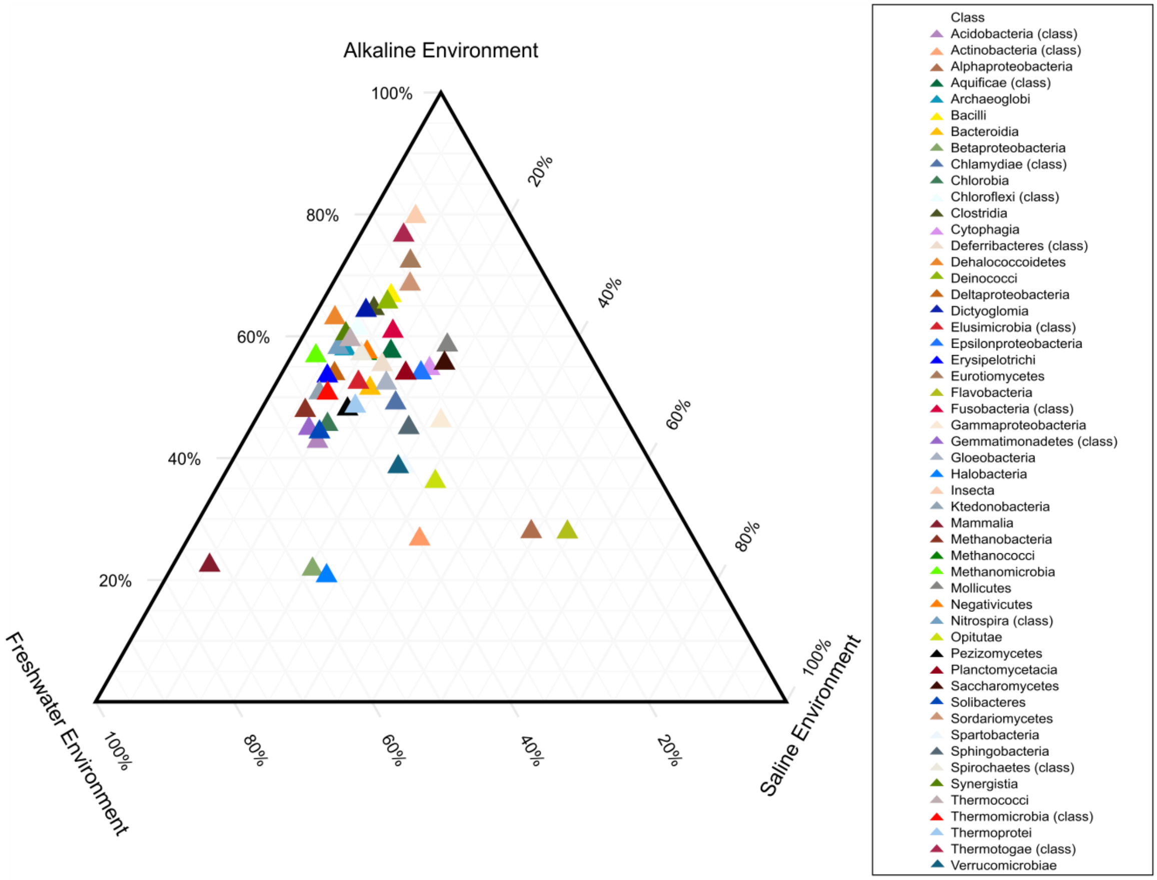

Supplementary Figure S5. Ternary plot indicating the distribution of Osmotic Stress, Oxidative stress, and Resistance to antibiotics and toxic compounds among various ecosystem datasets.

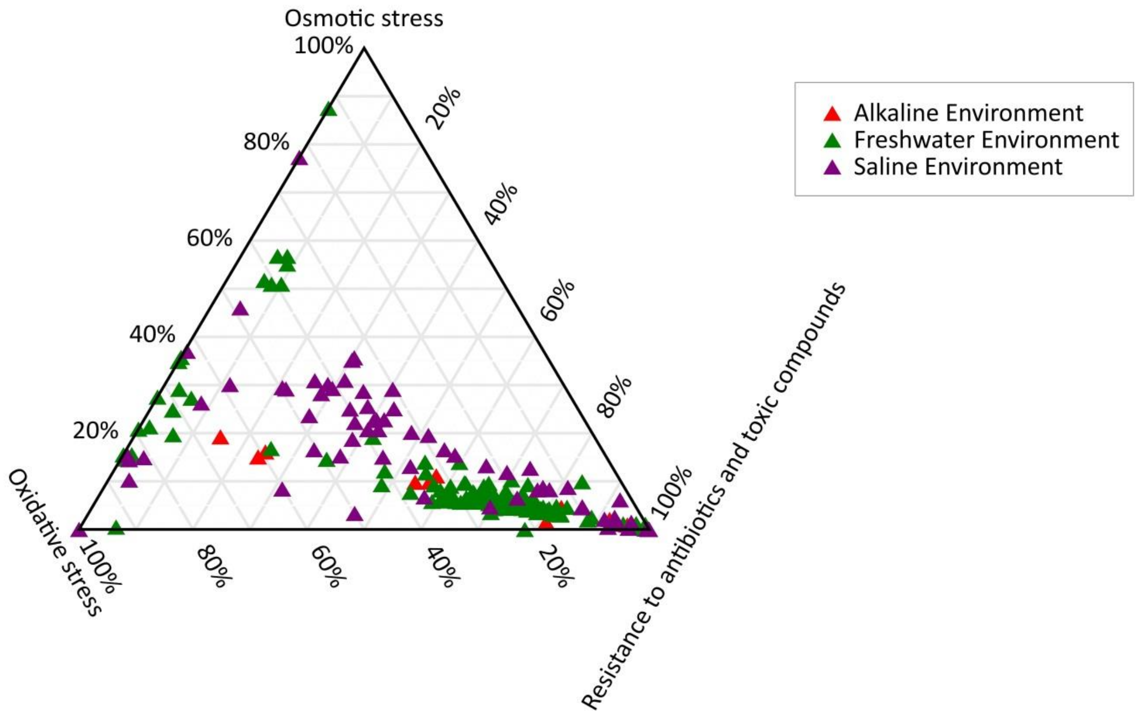

Supplementary Figure S6. Ternary plot indicating the distribution of Dormancy and Sporulation, Metabolism of aromatic compounds, and Photosynthesis among various ecosystem datasets.

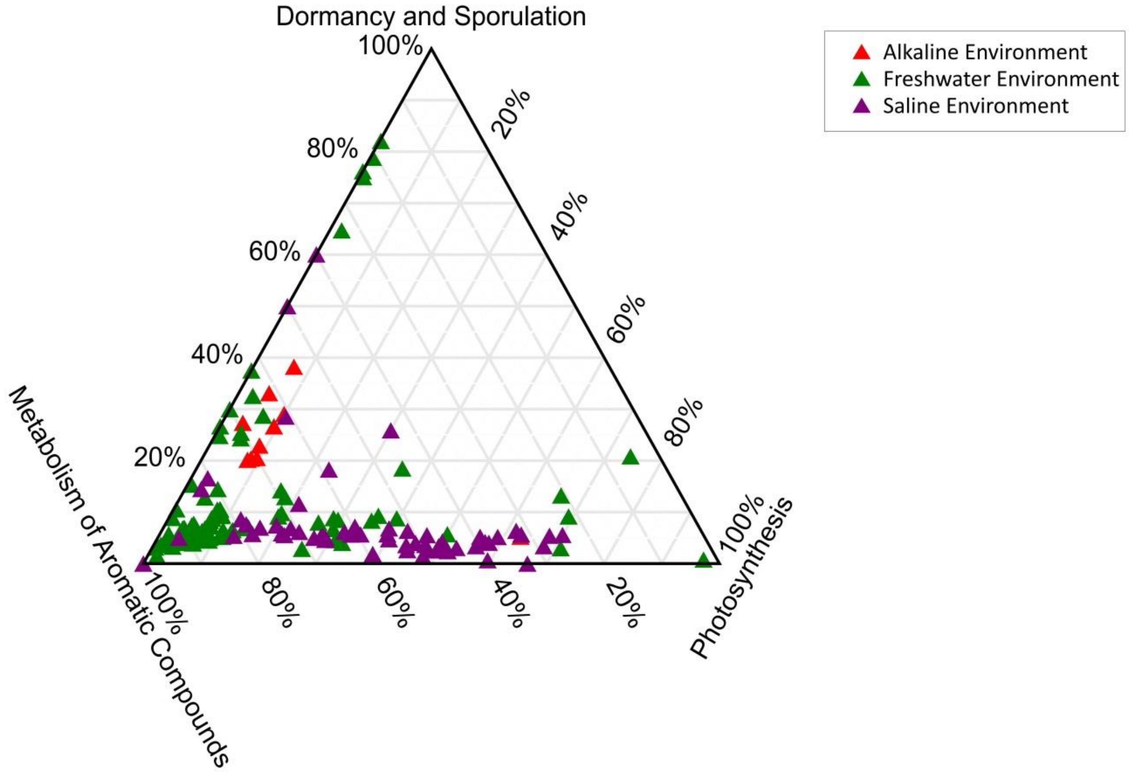

Supplement: Supplementary file 1 [file Data_Sheet_1.pdf]
